# Long-term high-grain diet alters ruminal pH, fermentation, and epithelial transcriptomes, leading to restored mitochondrial oxidative phosphorylation in Japanese Black cattle

**DOI:** 10.1038/s41598-020-63471-0

**Published:** 2020-04-14

**Authors:** Toru Ogata, Hiroki Makino, Naoki Ishizuka, Eiji Iwamoto, Tatsunori Masaki, Keiichiro Kizaki, Yo-Han Kim, Shigeru Sato

**Affiliations:** 1 United Graduate School of Veterinary Sciences, Gifu University, Gifu, 501-1193 Japan; 2 Cooperative Department of Veterinary Medicine, Faculty of Agriculture, Iwate University, Morioka, Iwate, 020-8550 Japan; 3 Hyogo Prefectural Technology Center of Agriculture, Forestry and Fisheries, Hyogo, 679-0198 Japan

**Keywords:** Agricultural genetics, Transcriptomics

## Abstract

To increase intramuscular fat accumulation, Japanese Black beef cattle are commonly fed a high-grain diet from 10 to 30 months of age. Castrated and fistulated cattle (*n* = 9) were fed a high-concentrate diets during the early, middle, and late stages consecutively (10–14, 15–22, 23–30 months of age, respectively). Ruminal pH was measured continuously, and rumen epithelium and fluid samples were collected on each stage. The 24-h mean ruminal pH during the late stage was significantly lower than that during the early stage. Total volatile fatty acid (VFA) and lactic acid levels during the late stage were significantly lower and higher, respectively, than those during the early and middle stages. *In silico* analysis of differentially expressed genes showed that “Oxidative Phosphorylation” was the pathway inhibited most between the middle and early stages in tandem with an inhibited upstream regulator (PPARGC1A, also called PGC-1α) but the most activated pathway between the late and middle stages. These results suggest that mitochondrial dysfunction and thereby impaired cell viability due to acidic irritation under the higher VFA concentration restored stable mitochondrial oxidative phosphorylation and cell viability by higher lactic acid levels used as cellular oxidative fuel under a different underlying mechanism in subacute ruminal acidosis.

## Introduction

A high-grain diet promotes the growth, productivity, and quality of meat or milk production in beef and dairy cattle. On a high-grain diet, organic acids such as volatile fatty acids (VFAs) and lactic acid accumulate in the rumen^1,2^. Ruminal pH is critical in the maintenance of normal, stable fermentation, microbial populations, and absorptive function^2–4^, and is determined by the balance between acid production by microbes and acid removal by absorption, neutralization, and clearance^1,5,6^, in a host–microbiome interaction^7^. The occurrence of subacute ruminal acidosis (SARA) defined by a rumen pH <5.6 is due to the non-physiological accumulation of VFAs, while ruminal acidosis defined by a rumen pH <5.0 is associated with accumulated lactic acid in the rumen^2^.

Several short- (days) or mid-term (weeks) studies have shown that SARA or rumen acidosis challenges affected the rumen epithelial structure, gene expression, and transcriptomes^8–10^. For example, in one study, 3 weeks of a high-grain diet (65% grain) compromised the structural integrity and led to the appearance of undifferentiated cells near the stratum corneum of the rumen papillae in non-lactating dairy cattle^8^. In another, the rumen papillae in dairy cattle fed a total mixed ration had increased epithelial desquamation and sloughing scores during early lactation, as well as upregulation of genes encoding desmosome assembly (despoglein1 and corneodesmosin), epidermal growth factor (EGF) signaling (epiregulin), transforming growth factor β (TGFB) signaling (connective tissue growth factor and TGFB1), and the insulin-like growth factor (IGF)-axis (IGF binding protein 2), and downregulation of genes encoding (EGF) signaling (EGF receptor) and IGF-axis (IGF binding protein 3) compared to those before lactation^9^. In addition, Kim *et al*.^10^ reported that the higher acidity caused by feeding only calf starter damages the rumen mucosal barrier and stimulates the proliferation of rumen epithelial cells regulated by growth factor (TGFB1) and signaling pathways (EGF and IGF-binding protein) during the weaning transition.

Japanese Black cattle can accumulate large amounts of intramuscular fat^11^. The fattening of Japanese Black cattle typically begins at about 10 months of age and is completed by about 30 months. During the fattening period, the cattle are generally fed a high-grain diets containing low levels of vitamin A, to induce greater intramuscular fat deposition, leading to highly marbled meat, although hypovitaminosis A may be related to the occurrence of hepatic disorders^12^. Therefore, Japanese Black cattle are continuously fed a high-grain diet (concentrate ratio 70–90%) with a low proportion of forage diet for about 20 months in contrast to dairy cattle fed mid-grain diets to high-grain diets (concentrate ratio 40–70%) or total mixed rations for about 10 months at most after calving^9,13^. However, little is known about the effects of the rumen environment on Japanese Black cattle fattening, in terms of the ruminal pH, fermentation, and epithelial transcriptomes.

Therefore, we explored the effects of the ruminal pH, fermentation, and epithelial transcriptomic dynamics on the fattening of 10-month-old Japanese Black beef cattle fed a long-term (20-month) high-grain diet. The findings increase our understanding of the ruminal pH, fermentation, and epithelial transcriptomes as an adaptation to a long-term high-grain diet, contributing to understand their interactions (i.e., host-microbiome interactions) in Japanese Black beef cattle.

## Results

### Daily dietary intake, ruminal pH, total VFA, LPS, and LBP

In this study, we re-analyzed our previously published data regarding rumen fermentation parameters^14^ to evaluate the relationships between ruminal pH and candidate gene expression in the RE.

No adverse health condition throughout the study period and effects of ruminal cannulation after surgery were observed for any of the cattle. The daily intake and rumen fermentations were described previously^14^. Briefly, the organic matter basis concentrate intake was significantly (*P* < 0.05) higher during the Middle stage than during the early and late stages (7.6 vs. 6.0 and 6.1 kg/day, respectively), and the forage diet intake was significantly (*P* < 0.05) higher during the early stage than the middle and late stages (2.1 vs. 1.1 and 1.0 kg/day, respectively). Nutrient adequacy rates of dry matter (DM) and total digestible nutrients (TDN) were calculated based on the total consumption amounts of concentrate diet and forage.

**Figure 1:**
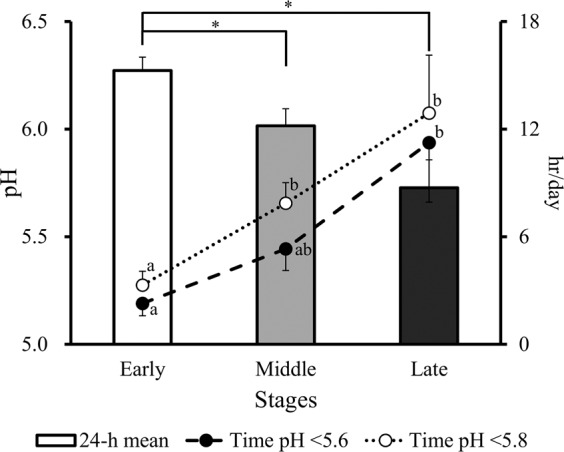
The 24 h mean ruminal pH (bar graph) and duration of time where pH was <5.6 (dotted line) and <5.8 (dashed line) in Japanese Black beef cattle during the early, middle, and late fattening stages. Values are the mean ± SE. *Significantly (*P* < 0.05) different in the bar graph. ^a,b^Different superscripts are significantly (*P* < 0.05) different in the line graph.

No change in pH was observed during calibration. The 24 h mean ruminal pH was significantly (*P* < 0.05) lower during the middle and late stages than during the early stage (Fig. 1). Accordingly, the duration of pH <5.6 was significantly (*P* < 0.05) longer during the late stage than during the early stage, and the durations of pH <5.8 were significantly (*P* < 0.05) longer during the middle and late stages than during the early stage. The 1 h mean ruminal pH was significantly (*P* < 0.05) lower during the late stage than during the early and middle stages, and was significantly (*P* < 0.05) lower during the middle stage than during the early stage (Fig. S1).

**Figure 2:**
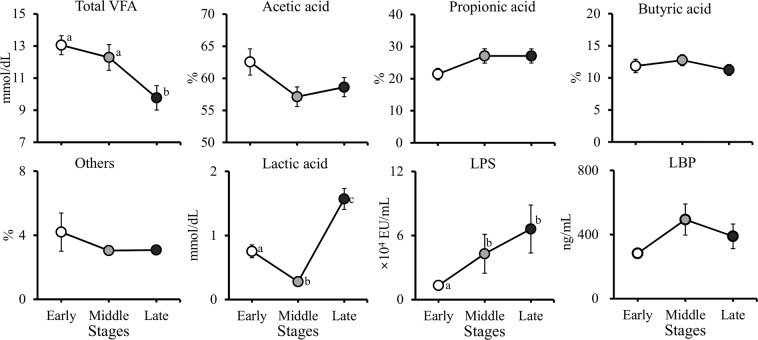
Changes in rumen total volatile fatty acid (VFA), lactic acid, and lipopolysaccharide binding protein (LBP) concentrations, and lipopolysaccharide (LPS) activity in Japanese Black beef cattle during the early, middle, and late fattening stages. ^a,b,c^Different superscripts are significantly different (*P* < 0.05). Values are the mean ± SE.

The total VFA concentration was significantly (*P* < 0.05) lower during the late stage than during the early and middle stages (Fig. 2). The lactic acid concentration was significantly (*P* < 0.05) higher during the late stage than during the early and middle stages, and was significantly (*P* < 0.05) lower during the middle stage than during the early stage. The rumen lipopolysaccharide (LPS) activities were significantly (*P* < 0.05) higher during the middle and late stages than during the early stage. However, the proportions of individual VFAs and peripheral blood LPS-binding protein (LBP) concentration did not differ (*P* > 0.10) among the fattening stages (Fig. 2).

### Microarray and pathway analyses of the rumen epithelium

**Figure 3:**
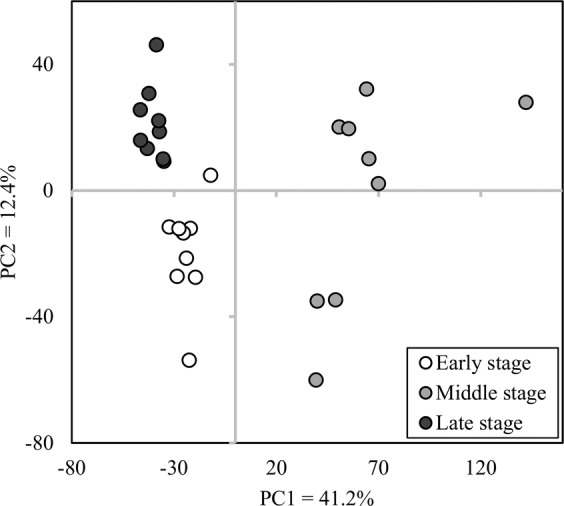
Principal component analyses plots of Japanese Black beef cattle during the early (white), middle (gray), and late (black) fattening stages. PC1 and PC2 are principal components 1 and 2, respectively.

In all, 3,570, 3,856, and 2,477 DEGs (FDR corrected *P* < 0.05) were identified when comparing the middle and early (first period), late and middle (second period), and late and early (total period) stages, respectively. There were 873 DEGs (315 upregulated) during the first period, 1,216 DEGs (656 upregulated) during the second period, and 115 DEGs (34 upregulated) during the total period, and they were mapped to molecular functions (FDR corrected *P* < 0.05, fold change ≥2; Table S3). Twenty-two candidate genes were common to all stages (FDR corrected *P* < 0.05, fold change ≥2), and all of the upregulated or downregulated DEGs during the first period were reversely downregulated or upregulated during the second and total periods (Table S4). Transporter gene expression of the rumen epithelium (RE) included 22 different soluble carrier (SLC) family members, and they were significantly changed during the first (2 upregulated, 11 downregulated), second (12 upregulated, 7 downregulated), and total (1 upregulated, 2 downregulated) periods (Table 1). The principal component analyses (PCA) plots of the fattening stages were similar within and distinct between stages, particularly in the middle versus late and early stages (Fig. 3).

**Table 1:** Differentially expressed genes (FDR corrected P < 0.05, fold change ≥ 2) encoding rumen epithelial transporters in the comparisons of the middle and early, late and middle, and late and early stages in Japanese Black beef cattle.

Gene Symbol	Fold Change			*P*-value			Gene Name
	Middle vs. Early	Late vs. Middle	Late vs. Early	Middle vs. Early	Late vs. Middle	Late vs. Early	
SLC5A12	2.24	−2.54		1.84.E-03	1.18.E-04		solute carrier family 5 (sodium/glucose cotransporter), member 12
SLC6A8	−2.01			1.28.E-03			solute carrier family 6 (neurotransmitter transporter, creatine), member 8
SLC7A6OS		−2.05			3.23.E-03		solute carrier family 7, member 6 opposite strand
SLC9A6	−4.08	4.08		7.87.E-04	1.64.E-05		solute carrier family 9 (sodium/hydrogen exchanger), member 6
SLC10A5	2.72			2.05.E-04			solute carrier family 10, member 5
SLC22A5		−3.68			1.89.E-08		solute carrier family 22 (organic cation/carnitine transporter), member 5
SLC22A8	−3.50	6.75		2.73.E-03	5.26.E-07		solute carrier family 22 (organic anion transporter), member 8
SLC22A18	−2.49	2.20		2.05.E-04	8.00.E-06		solute carrier family 22, member 18
SLC23A3		−2.32			1.46.E-04		solute carrier family 23 (nucleobase transporters), member 3
SLC25A5	−3.30	2.99		4.76.E-04	2.17.E-04		solute carrier family 25 (mitochondrial carrier; adenine nucleotide translocator), member 5
SLC25A6	−5.59	4.80		5.10.E-04	5.40.E-05		solute carrier family 25 (mitochondrial carrier; adenine nucleotide translocator), member 6
SLC25A11	−2.48	2.38		6.02.E-04	3.30.E-05		solute carrier family 25 (mitochondrial carrier; oxoglutarate carrier), member 11
SLC25A17		2.10			1.19.E-04		solute carrier family 25 (mitochondrial carrier; peroxisomal membrane protein, 34 kDa), member 17
SLC26A3	−6.38	10.80		3.45.E-03	6.37.E-05		solute carrier family 26, member 3
SLC28A3	−6.71	10.20		1.35.E-03	9.92.E-06		solute carrier family 28 (sodium-coupled nucleoside transporter), member 3
SLC29A4		2.45	2.32		7.62.E-07	1.60.E-03	solute carrier family 29 (nucleoside transporters), member 4
SLC35A5	−2.73	2.52		8.02.E-03	3.88.E-03		solute carrier family 35, member A5
SLC35C1		−2.09			2.44.E-06		solute carrier family 35, member C1
SLC35F5	−2.63	3.83		6.84.E-04	5.55.E-06		solute carrier family 35, member F5
SLC39A7		−2.46			2.03.E-08		solute carrier family 39 (zinc transporter), member 7
SLC39A11		−2.17	−2.92		7.98.E-06	9.93.E-05	solute carrier family 39 (metal ion transporter), member 11
SLCO2A1			−2.08			4.90.E-04	solute carrier organic anion transporter family, member 2A1

**Figure 4:**
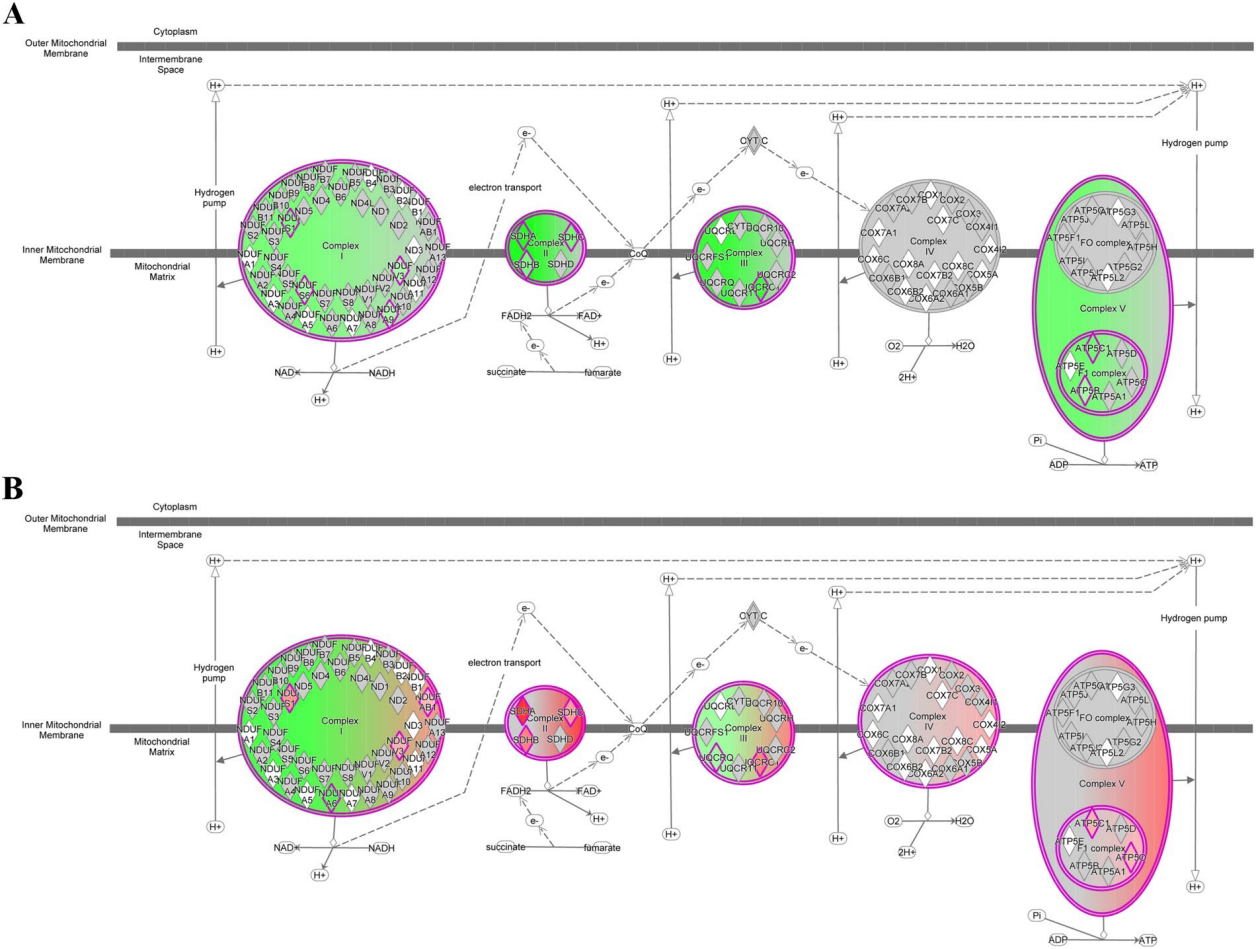
Oxidative phosphorylation in the canonical pathway analyses of Japanese Black beef cattle using IPA software (QIAGEN Inc., https://www.qiagenbioinformatics.com/products/ingenuitypathway-analysis) The IPA knowledge base was used to analyze DEGs with raw estimated fold changes ≥2 by comparing the (**A**) middle and early (z-score = −3.32, *P* = 3.72 × 10^−3^), and (**B**) late and middle (z-score = 2.50, *P* = 6.61 × 10^−3^) stages. Green represents the downregulation of integrated genes and red represents upregulation of genes.

Canonical pathways of DEGs were identified (*P* < 0.05, z-score ≥ 2 or ≤ −2) using IPA software. There was no activated canonical pathway during the first period, whereas “Oxidative Phosphorylation” was the most inhibited (z-score = –3.32, *P* = 3.72 × 10^−3^) pathway during the same period (Table 2, Fig. 4A). During the second period, “Oxidative Phosphorylation” was activated the most (z-score = 2.50, *P* = 6.61 × 10^−3^; Fig. 4B), and no inhibited pathway was identified (Table 2). Table 3 summarizes the genes integrating oxidative phosphorylation pathway during the first (11 downregulated) and second periods (11 upregulated, 2 downregulated).

**Table 2:** Canonical pathways activated (z-score ≥2) and inhibited (z-score ≤ −2) generated by Ingenuity Pathway Analysis (IPA) in comparisons of the early and middle stages and late and middle stages.

Canonical Pathways	-log(p-value)	zScore^a^	Ratio	Molecules
Middle *vs*. Early stages				
iNOS Signaling	2.10	−2.00	0.133	FOS, IFNGR1, IFNGR2, CALML5, JAK1, LBP
CCR5 Signaling in Macrophages	1.93	−2.00	0.096	GNAI1, FOS, GNG5, CCL5, CD3G, GNG7, CALML5, GNB1, CACNG1
IL-22 Signaling	1.87	−2.00	0.167	IL10RB, AKT1, IL22RA1, JAK1
NRF2-mediated Oxidative Stress Response	3.39	−2.11	0.094	SQSTM1, ABCC4, AKT1, UBE2E3, NQO2, HSPB8, MAP2K1, PPIB, FOS, AKR7A2, SOD2, CCT7, ACTB, PTPN11, CAT, EPHX1, AKR1A1, VCP, HERPUD1
Insulin Receptor Signaling	1.51	−2.11	0.075	AKT1, PTPN11, PPP1R12A, PPP1CA, PPP1CB, PRKAR1B, JAK1, SCNN1A, MAP2K1, SYNJ1, PPP1R11
Oxidative Phosphorylation	2.43	−3.32	0.101	SDHB, UQCRC1, NDUFA9, SDHA, NDUFV3, NDUFS6, ATPAF1, ATP5F1C, ATP5F1B, SDHC, NDUFS1
Late *vs*. Middle stages				
Oxidative Phosphorylation	2.18	2.50	0.119	COX11, UQCRC1, SDHA, NDUFV3, NDUFAB1, NDUFS1, SDHB, UQCRQ, ATP5PO, NDUFA6, ATPAF1, ATP5F1C, SDHC
Role of p14/p19ARF in Tumor Suppression	1.46	2.45	0.133	SENP3, FGFR1, UBTF, PTPN11, MDM2, TTF1
Endocannabinoid Cancer Inhibition Pathway	1.57	2.32	0.095	MAP2K5, GNAI1, FGFR1, SMPD2, MAP2K1, RHOA, PTPN11, CREB3, SPTLC1, TWIST2, PRKAR1B, DDIT3, VEGFB, CASP8, PRKAR2A

^a^Values indicate a statistically significant match between up- and down-regulation patterns.

**Table 3:** Genes (raw estimated fold change ≥2, FDR corrected *P* < 0.05) integrating the oxidative phosphorylation canonical pathway generated by Ingenuity Pathway Analysis (IPA) in comparisons of the early and middle stages and late and middle stages.

Gene Symbol	Fold change		*P*-value		Gene Name	Location	Type
	Middle *vs*. Early	Late *vs*. Middle	Middle *vs*. Early	Late *vs*. Middle			
ATP5F1C	−3.39	2.16	2.38.E-04	3.86.E-05	ATP synthase F1 subunit gamma	Cytoplasm	Transporter
ATP5F1B	−2.16		7.60.E-04		ATP synthase F1 subunit beta	Cytoplasm	Transporter
ATP5PO		2.05		4.86.E-05	ATP synthase peripheral stalk subunit OSCP	Cytoplasm	Transporter
ATPAF1	−3.40	3.94	4.75.E-04	6.83.E-06	ATP synthase mitochondrial F1 complex assembly factor 1	Cytoplasm	Other
COX11		2.33		2.69.E-04	cytochrome c oxidase copper chaperone COX11	Cytoplasm	Enzyme
NDUFA6		−4.22		2.33.E-08	NADH:ubiquinone oxidoreductase subunit A6	Cytoplasm	Enzyme
NDUFA9	−2.06		4.26.E-04		NADH:ubiquinone oxidoreductase subunit A9	Cytoplasm	Enzyme
NDUFAB1		2.02		3.42.E-04	NADH:ubiquinone oxidoreductase subunit AB1	Cytoplasm	Enzyme
NDUFS1	−3.20	3.07	2.89.E-05	1.95.E-08	NADH:ubiquinone oxidoreductase core subunit S1	Cytoplasm	Enzyme
NDUFS6	−2.22		2.40.E-03		NADH:ubiquinone oxidoreductase subunit S6	Cytoplasm	Enzyme
NDUFV3	−2.06	2.18	7.68.E-03	3.07.E-03	NADH:ubiquinone oxidoreductase subunit V3	Cytoplasm	Enzyme
SDHA	−7.32	7.18	2.73.E-06	2.14.E-10	succinate dehydrogenase complex flavoprotein subunit A	Cytoplasm	Enzyme
SDHB	−3.18	3.57	4.64.E-04	9.90.E-06	succinate dehydrogenase complex flavoprotein subunit B	Cytoplasm	Enzyme
SDHC	−3.42	2.78	3.90.E-05	1.27.E-07	succinate dehydrogenase complex flavoprotein subunit C	Cytoplasm	Enzyme
UQCRC1	−4.71	3.57	1.78.E-04	1.42.E-05	ubiquinol-cytochrome c reductase core protein 1	Cytoplasm	Enzyme
UQCRQ		−2.25		1.86.E-05	ubiquinol-cytochrome c reductase complex III subunit VII	Cytoplasm	Enzyme

**Figure 5:**
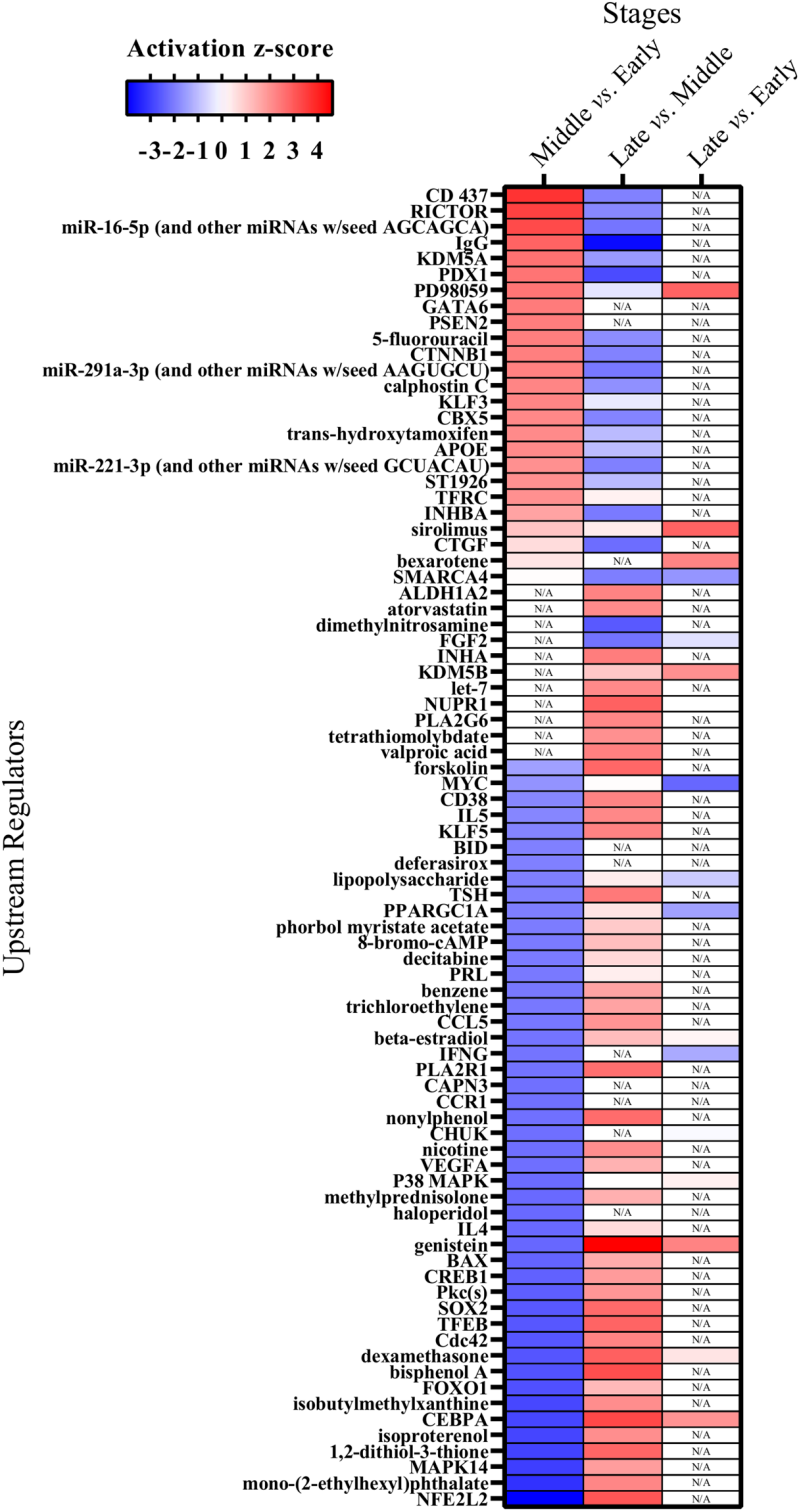
Heatmap of upstream regulators (*P* < 0.05) generated by IPA software (QIAGEN Inc., https://www.qiagenbioinformatics.com/products/ingenuitypathway-analysis) in comparisons of the early, middle, and late stages in Japanese Black beef cattle. Cell colors are based on the activation z-score. Blue represents a negative z-score and red represents a positive z-score. N/A, not applicable.

Upstream regulator analyses based on the DEGs revealed significant activation or inhibition (*P* < 0.05, z-score ≥ 2 or ≤ −2, respectively) within each comparison (Fig. 5). During the first period, 6-[3-(1-adamantyl)-4-hydroxyphenyl]-2-naphthalene carboxylic acid (CD437; z-score = 3.67, *P* = 2.69 × 10^−6^) was the upstream regulator activated the most and nuclear factor, erythroid 2 like 2 (NFE2L2; z-score = −3.99, *P* = 8.71 × 10^−6^) inhibited the most. During the second period, the most activated and inhibited upstream regulators were genistein (z-score = 4.61, *P* = 6.93 × 10^−5^) and immunoglobulin G (IgG; z-score = −3.84, *P* = 2.64 × 10^−4^), respectively, and those during the overall period were 2-(2′-Amino-3′-methoxyphenyl)-oxanaphthalen-4-one (PD98059; z-score = 2.82, *P* = 2.78 × 10^−3^) and MYC proto-oncogene, bHLH transcription factor (MYC; z-score = −2.39, P = 2.97 × 10^−2^). Most of the significantly activated or inhibited upstream regulators during the first period were subsequently inhibited or activated, respectively, during the second period (Fig. 5).

### Validation of DEGs by qPCR

Fig. S2 shows the fold changes in the expression of nine genes of interest determined by microarray analyses and quantitative real-time polymerase chain reaction (qPCR). For all nine genes, the direction of the estimated fold changes was consistent in the microarray and qPCR analyses.

## Discussion

During the fattening period, Japanese Black beef cattle are raised on a high-grain diet between 10 and 30 months of age to increase intramuscular fat accumulation. In this study, samples were collected throughout the entire fattening period to explore the long-term changes in ruminal pH and fermentation as described previously^14^, and their consequences for rumen epithelial transcriptomic dynamics. In cattle, a ruminal pH below the physiological range of 5.8–6.5^6^ can induce inflammatory responses^15^, and structural or transcriptomic changes in the RE^8–10^. Consistent with a previous study^14^, the 24 h mean ruminal pH decreased gradually as the lengths of time at pH <5.6 and <5.8 increased during the middle and late stages of fattening as a result of a long-term high-grain diet. These observations are sufficient to diagnose SARA during the middle and late stages (ruminal pH <5.6 or <5.8 for more than 3 or 5 h per day, respectively^16,17^;). However, lactic acid is 10 times less protonated than VFA in the rumen (pKa 4.9 vs. 3.9; 2), and its concentration peaked during the late stage. Therefore, it is reasonable to postulate that different mechanisms underlie the low ruminal pH in the late (lactic acid accumulation) versus early and middle stages (total VFA accumulation) as an adaptation of rumen bacterial community to and endurance of a long-term, harsh ruminal environment in Japanese Black beef cattle^14^. However, lower ruminal pH during the late stage did not disrupt the gastrointestinal barrier or induce higher LBP levels in peripheral blood, despite inducing significantly higher LPS levels in the rumen^14^.

Transporter genes (SLC family) are considered to be responsible for pH regulation by rumen epithelial absorption^10^. A low ruminal pH or high-grain diet enhances rumen epithelial absorption via enhanced transporter gene expression, such as monocarboxylate transporter (MCT1; 5), sodium hydrogen exchanger isoform 3 (NHE3^18^;), and downregulated in adenoma (DRA^19^;). In the present study, we observed the opposite, i.e., most of the 22 different SLC family genes identified were downregulated (11/13) during the first period and then upregulated (12/19) during the second period. In detail, the expression of SLC9A6 (NHE6) and SLC26A3 (DRA) were downregulated (fold change = −4.08 and −6.71, respectively) during the first period, while they were upregulated (fold change = 4.08 and 10.80, respectively) during the second period. These results suggest that expression of genes encoding rumen epithelial absorption was suppressed by acidotic insult during the first period, whereas it was restored during the second, enhancing VFA absorption or transporter gene expression in the rumen.

Interestingly, different types of SARA in the earlier and later stages had completely opposite consequences on the candidate gene expression (DEGs) and its analysis results (canonical pathway and upstream regulators). Furthermore, PCA plots also suggested that the middle stage was the stage most affected by long-term high-grain diet. LaMonte *et al*.^20^ suggested that acidosis alters cellular metabolism, such as the pentose phosphate pathway and glutaminolysis, to mitigate increased stress due to reactive oxygen species (ROS). A high-starch diet also leads to increased oxidative stress and changes in oxidative phosphorylation to alleviate the toxic effects of ROS^21^. Moreover, VFAs, particularly butyric acid, are catabolized within rumen epithelial cells via oxidative pathways, which generate ROS in the RE^22^. However, mitochondrial superoxide dismutase 2 (SOD2; fold change = −2.29, *P* = 2.16 × 10^−4^) was significantly downregulated during the first period, and then it was upregulated (fold change = 2.32, *P* = 5.24 × 10^−5^) again simultaneously with SOD1 (fold change = 1.52, *P* = 1.77 × 10^−3^) during the second period. These results were supported by the fact that the top upregulated candidate gene during the first period, NADH dehydrogenase subunit 6 (ND6), was associated with the significant canonical pathway “Mitochondrial Dysfunction” (*P* = 2.69 × 10^−7^; data not shown). Although excess oxidative stress is inferred from the literatures^22–24^, the responses of rumen epithelial cells to oxidative stress might be depressed during the first period in contrast to a short-term (17 days) SARA challenge in dairy cows^21^. These results also imply mitochondrial metabolic dysfunction (i.e., handling oxidative stress), suggesting greater rumen epithelial cell vulnerability to cellular damage by acidotic insult^25^.

Mitochondrial proficiency and ROS detoxification are critical for cancer cell viability^26^, and proliferating cancer cells must preserve intracellular ATP and NADPH levels through mitochondrial oxidative phosphorylation and the prevention of excessive ROS accumulation^27^. *In silico* analyses of the DEGs also suggested that the results were in line with mitochondrial dysfunction during the first period. For example, “Oxidative Phosphorylation”, in response to peroxisome proliferator-activated receptor γ coactivator 1α (PGC-1α; 29), was the most inhibited canonical pathway with coordinated inhibition of the upstream regulator PPARGC1A (also called PGC-1α) during the first period. In addition, reduced expression of PGC-1^26,28^ and decreased oxidative phosphorylation by coordinated downregulation of PGC-1α-responsive genes^29^ are responsible for greater insulin resistance, which might be consistent with the inhibited “Insulin Receptor Signaling” canonical pathway in the present study. Therefore, long-term high-grain diet induced excessive oxidative stress in the RE due to the higher acidity and VFA metabolism, resulting in mitochondrial oxidative phosphorylation dysfunction via the downregulation of ubiquinone oxidoreductase subunits, which are cell survival factors^30^. Activation of upstream regulators such as CD437 (induction of cellular apoptosis and cell cycle arrest in cancer cells^31^;) and inhibition such as NFE2L2 (regulator for normal mitochondrial biogenesis^32^;) may indicate regulation of cell proliferation, apoptosis, and mitochondrial biogenesis in the RE. Collectively, the *in silico* analyses suggest impaired cellular viability and vulnerability to cellular damage due to acidotic irritation during the first period.

During the second period, however, the rumen epithelial transcriptomes restored cellular functions with changes in the underlying mechanism of SARA. The significantly higher lactic acid concentration during the late stage seemed to affect the responses of rumen epithelial cells to acidic irritation differently compared to the early and middle stages. Riemann *et al*.^33^ reported that extracellular acidosis results in a rapid, sustained decrease in intracellular pH (pH_i_) and tissue hypoxia, and induces increased transcription factor phosphorylation and tumor cells transcriptional activity^33^. The involvement of lactic acid in cancer has attracted attention only recently, and lactic acid directly contributes to tumor growth and progression (i.e., the lactate anion and protons) as an oxidative fuel and support for angiogenesis and metastasis^34,35^. For example, extracellular lactic acid acidosis causes a lower pH_i_ and higher intracellular lactate concentration, and rescues 4T1 (murine breast cancer) cells from glucose deprivation^36^. In addition, lactic acidosis induces a higher oxidative phosphorylation rate in ATP production in nine different cell lines compared to without lactic acidosis^37^, consistent with the activation of “Oxidative Phosphorylation” during the second period. Although our hypothesis may be preliminary and it is necessary to clarify the positive correlation between the ruminal and intracellular lactic acid concentrations, we postulate that the rumen epithelial transcriptomes were influenced, at least in part, by the significantly increased level of lactic acid in the rumen, resulting in greater cell viability by restoring the mitochondrial oxidative phosphorylation pathway. Our hypothesis may also be supported by the most activated (genistein, tyrosine kinase inhibition that enhances apoptosis and cell cycle arrest in cancer cells^38,39^;) and inhibited (IgG, malignant factor for colorectal cancer cells^40^;) upstream regulators during the second period.

Finally, activation of upstream regulators such as PD98059 (a mitogen-activated protein kinases kinase inhibitor^41^;), sirolimus (immunosuppressant that inhibits cell cycle progression^42^;), bexarotene (anti-cancer action by inducing T-cell lymphoma cell apoptosis^43^;), and KDM5B (critical regulator of genome stability and DNA repair ^44^;) and inhibition such as MYC (transcription regulator suppressed by intracellular butyrate^45^;) throughout the overall study period implies moderate restoration of cellular functions in response to long-term high-grain diet in Japanese Black cattle during the fattening period. However, the effects of gene expression on SARA occurrence or long-term high-grain diet feeding remain unclear, and further studies are required to provide host-side elucidation of host-microbiome interactions.

## Conclusions

In Japanese Black beef cattle, long-term feeding of a high-grain diet (at 10–30 months of age) induced SARA due to the total VFA production during the early and middle stages and due to lactic acid production during the late stage, suggestive of different mechanisms underlying SARA. Total VFA induced a low ruminal pH, which inhibited the oxidative phosphorylation pathway, and was associated with mitochondrial dysfunction and thereby impaired cell viability of the RE during the earlier fattening stage. By contrast, higher lactic acid levels used as cellular oxidative fuel restored the oxidative phosphorylation pathway and cellular viability during the latter fattening stages. Therefore, the specialized fattening technique applied to Japanese Black beef cattle results in unique changes in rumen fermentation characteristics, influencing epithelial transcriptomes through the interaction with altered rumen fermentation as a response to a long-term high-grain diet.

## Methods

The experimental protocol was approved by the Iwate University Laboratory Animal Care and Use Committee (A201720; Morioka, Japan). All animal experiments adhered to the animal experiment policy of Hyogo Prefectural Technology Center for Agriculture, Forestry and Fisheries (Hyogo Prefecture, Japan).

### Animals and experimental design

The experimental animals and designs were described previously^14^. Briefly, nine castrated (at age 5–6 months) and subsequently fistulated (at age 12 months) Japanese Black beef bull cattle were housed with free access to food and water throughout the study (10–30 months of age). The fattening period was subdivided into the early, middle, and late stages (10–14, 15–22, and 23–30 months of age, respectively) according to general agreement in Japan^12,14^. Cattle were fed concentrate and forage (rice straw) diets during all three stages, and the amount of concentrate diet was increased gradually throughout the experimental period. The forage-to-concentrate ratio was 26:74, 13:87, and 14:86 during the early, middle, and late stages, respectively. The concentrate diet was composed of barely, steam-flaked corn, wheat bran, and soybean meal and contains 71.2% total digestible nutrient (TDN) and 15.7% crude protein (CP), 72.2% TDN and 13.9% CP, and 72.8% TDN and 12.0% CP during the early, middle, and late stage, respectively. The mean ± SE body weight of the cattle was 335 ± 4.4, 439 ± 7.6, 562 ± 11.6, and 712 ± 18.5 kg on prior to the experiment (10 months of age), and early (14 months of age), middle (21 months of age), and late (29 months of age) fattening stage sampling days, respectively. The forage diet was supplied daily at 0930 and 1530 h in two equal portions, and the concentrate was supplied 1 h after the rice straw to maximize forage diet intake and to prevent excessive consumption of concentrate diet during the early stage. Abnormalities of body condition (body temperature, appetite, hydration, and defecation) were observed daily throughout the study period. The intakes of concentrate and rice straw diets were recorded daily during the final 7 days of the early, middle, and late fattening stages. Table S1 shows the body weight, organic matter intake amount, and chemical composition of the fattening stage diets. Chemical composition of the diets was analyzed according to the official method analysis of the Association of Official Analytical Chemists (AOAC) that registered in the Official Method Feed Analysis of Japan^46^. The adequacy rate of diet was calculated based on the nutrient requirement of Japanese Feeding Standard for Beef cattle^47^.

### Sampling and measurements

The sample collections and measurements were described previously^14^. Briefly, ruminal pH was measured continuously every 10 min during the final 7 days of the early, middle, and late fattening stages using a radio transmission system (YCOW-S; DKK-TOA, Yamagata, Japan), as described previously^48^. A pH sensor was placed in the ventral sac of the rumen through the rumen fistula and calibrated at standard pH values of 4–7, before and after obtaining data in each fattening stage. Rumen fluid from the ventral sac of the rumen (adjacent to the pH sensor) and blood samples from the jugular vein were collected on day 4 of the pH measurements during the three stages, for analyses of total VFA, individual VFAs, lactic acid concentration, LPS activity, and LBP concentration. The fluid samples were immediately filtered through two layers of cheesecloth and stored at −80 °C until use. Blood samples were immediately centrifuged (1,500 × g, 15 min, 4 °C) to separate the plasma and then preserved at −80 °C until analyses.

For the VFA analyses, 1 mL 25% HO_3_P in 3 N H_2_SO_4_ was added to 5 mL rumen fluid. Total VFA and individual VFAs (acetic, propionic, and butyric acids) were separated and quantified by gas chromatography (GC-2014; Shimadzu, Kyoto, Japan) using a packed glass column (Thermon-3000; 3%) with a Shimalite TPA 60–80 mesh support (Shinwa Chemical Industries, Kyoto, Japan). For lactic acid analyses, fluid samples were centrifuged at 2,000×g for 15 min at 4 °C, and the concentration in the supernatant was determined using a commercial F-kit (D-lactate/L-lactate) (J.K. International, Tokyo, Japan). To measure rumen LPS activity, rumen fluid samples were centrifuged at 11,000×g for 15 min at 4 °C and assayed using a kinetic Limulus amebocyte lysate assay (Pyrochrome with Glucashield; Seikagaku, Tokyo, Japan) as described previously^49^. The plasma LBP concentration was measured using a commercial kit (HK503; HyCult Biotech, Uden, The Netherlands) as described elsewhere^50^.

### Transcriptome analyses of the rumen epithelium

The RE was biopsied from the ventral sac of the rumen at a site adjacent to the pH sensor simultaneously with rumen fluid sampling. A sterile, disposable biopsy punch 8 mm in diameter (Kai Industries, Tokyo, Japan) was used to scratch off the RE. Then the RE samples were washed three times in ice-cold PBS and immediately stored at −80 °C until use. Total RNA was extracted from the RE using TRIzol reagent (Invitrogen) as described previously^10^. The purity of the extracted RNA was increased using an RNeasy RNA Clean-up Kit (QIAGEN, Valencia, CA). Total RNA was quantified using a NanoDrop ND-1000 spectrophotometer (NanoDrop Technologies, Thermo Fisher Scientific, Waltham, MA), and its quality was assessed using a 2100 Bioanalyzer and RNA 6000 Nano LabChip Kits (Agilent Technologies, Palo Alto, CA); the mean RNA integrity value was 7.4 ± 0.4 (mean ± SE).

### Microarray and Ingenuity pathway Analyses approaches

A customized bovine oligonucleotide microarray comprising 15,268 genes (Agilent Technologies) was used for one-color microarray analyses to detect genes expressed in the RE^51^. Fluorescently labeled (cyanine 3) complementary cRNA probes were hybridized to the samples and washed using the Gene Expression Hybridization Kit and Gene Expression Wash Pack Kit (Agilent Technologies). The arrays were scanned using an Agilent Microarray Scanner (Agilent Technologies). Feature Extraction ver. 9.5 (Agilent Technologies) was used to process microarray images, align spots, and create raw numerical total spot intensity data. The microarray data from each sample were imported into GeneSpring 12.0 (Agilent Technologies) for use in the software’s normalization algorithm and for detection of candidate genes. Normalization was performed by dividing each measurement of each array by the median of all measurements in that array (per-chip normalization). The entire microarray data set has been deposited at the Gene Expression Omnibus (GEO) database. The GEO accession numbers are as follows: Platform, GPL22091; samples, GSM 3901089 to GSM 3901115; series, GSE133152.

Pathway and network analyses of DEGs were performed using Ingenuity Pathway Analyses (IPA) software (Ingenuity Systems, Redwood City, CA^52^;). Lists of DEGs identified by GeneSpring 12.0 (Agilent Technologies) that corresponded to raw estimated fold changes ≥2 were uploaded into the software application. The IPA knowledge base was used for the DEG enrichment analyses, and the canonical pathways, top upstream regulators, and statistical calculation were analyzed.

### Quantitative real-time PCR

Nine genes (both upregulated and downregulated) representing a range of fold changes in the microarray analyses were selected for validation by qPCR. Total RNA samples were treated with TURBO DNase (Applied Biosystems, Foster City, CA, USA) to remove contaminating DNA. Total RNA (800 ng) was converted into first-strand cDNA using the High-Capacity cDNA Reverse Transcription Kit (Applied Biosystems). The qPCR was performed using iQ SYBR Green Supermix (Bio-Rad, Hercules, CA) and the StepOne™ Plus Real-Time PCR system (Applied Biosystems) as described elsewhere^10^. Primers (Table S2) were designed using Primer Express software 3.0 (Applied Biosystems). Each sample contained 2 μl cDNA, 2× SYBR green, and 0.6 μM each primer in a final volume of 20 μl. Amplification conditions were as follows: 95 °C for 10 min followed by 40 cycles of 15 s at 95 °C and 1 min at 60 °C, with collection of fluorescence signal at the end of each cycle. For melting curve data, the temperature was increased from 65 to 95 °C in 0.5 °C increments^10^. The results were recorded as relative changes in gene expression normalized to glyceraldehyde-3-phosphate dehydrogenase (*GAPDH*), ribosomal protein L27 (*RPL27*), and β-actin (*ACTB*) by the 2^−△△Ct^ method^53^. We examined *GAPDH*, *RPL27*, and *ACTB* to evaluate their use as reference genes for qPCR and there were no significant differences among them. All qPCR experiments were performed in accordance to the guidelines for minimum information for publication of quantitative real-time PCR experiments^53,54^.

### Statistical analyses

The pH data were summarized 1 day before the sample collection to minimize the influence of opening the cannula stopper on the ruminal pH. The normality of the distributions of variables was assessed using the Shapiro–Wilk test. Significant differences in ruminal pH, duration of time where pH was <5.6 and 5.8, VFA components, lactic acid concentration, LPS activity, and peripheral LBP concentration among the early, middle, and late stages were evaluated using paired *t*-tests for normally distributed variables and the Wilcoxon rank-sum test for non-normal variables (Prism ver. 8.10; GraphPad Software, La Jolla, CA, USA). The microarray data were analyzed using paired Student’s *t*-tests with the Benjamini–Hochberg FDR multiple testing correction (FDR corrected *P* < 0.05) and summarized using GeneSpring 12.0 (Agilent Technologies). Fold changes were calculated by comparing the middle and early (first period), late and middle (second period), and late and early (total period) stages. The PCA plot coordinates were calculated using the devtools package with microarray data in R ver. 3.3.2 (http://www.r-project.org; R Foundation for Statistical Computing, Vienna, Austria). Significant differences were determined with a threshold of *P* < 0.05, while trends that suggested possible significance were identified at 0.05 < *P* < 0.10.

## Supplementary information

Supplementary Information.

## Data Availability

The entire microarray data set has been deposited at the Gene Expression Omnibus (GEO) database. The GEO accession numbers are as follows: Platform, GPL22092; samples, GSM 3901089 to GSM 3901115; series, GSE133152.
