# Supplementary material for: Long-term high-grain diet alters ruminal pH, fermentation, and epithelial transcriptomes, leading to restored mitochondrial oxidative phosphorylation in Japanese Black cattle
Source: Sci Rep. 2020 Apr 14;10:6381. doi: 10.1038/s41598-020-63471-0 (PMC7156705; doi:10.1038/s41598-020-63471-0)

Long-term high-grain diet alters ruminal pH, fermentation, and epithelial transcriptomes, leading to restored mitochondrial oxidative phosphorylation in Japanese Black cattle

Toru OGATA[1,2], Hiroki MAKINO[2], Naoki ISHIZUKA[2], Eiji IWAMOTO[3], Tatsunori MASAKI[3], Keiichiro KIZAKI[1,2], Yo-Han KIM[2*], and Shigeru SATO[1,2*]

[1]United Graduate School of Veterinary Sciences, Gifu University, Gifu, 501-1193, Japan

[2]Cooperative Department of Veterinary Medicine, Faculty of Agriculture, Iwate University, Morioka, Iwate 020-8550, Japan

[3]Hyogo Prefectural Technology Center of Agriculture, Forestry and Fisheries, Hyogo 679-0198, Japan

[*]Corresponding authors: haneey@iwate-u.ac.jp and sshigeru@iwate-u.ac.jp

Supplementary information

Supplementary Table S1. Body weight, dietary composition, and chemical analyses of the diet of Japanese Black beef cattle during the early, middle, and late fattening stages.

Supplementary Table S2. Gene symbols and sequences of the primers used for quantitative real-time PCR.

Supplementary Table S3. Differentially expressed genes (fold change ≥ 2, $P < 0.05$) uploaded into Ingenuity Pathway Analyses software in the comparisons of the middle and early, late and middle, and late and early stages.

Supplementary Table S4. Differentially expressed genes (fold change ≥ 2, $P < 0.05$) identified in comparisons of the middle and early, late and middle, and late and early stages.

Supplementary Figure S1. Diurnal changes in the 1 h mean rumen pH in Japanese Black beef cattle during the early, middle, and late fattening stages. ^{a,b,c}Different superscripts indicate significant ($P < 0.05$) differences between the middle and early, late and middle, and late and early stages. Values are the mean ± SE.

Supplementary Figure S2. Validation of gene expression levels in Japanese Black beef cattle during the early, middle, and late fattening stages by quantitative real-time PCR (qPCR). Fold changes are the differences in mean transcript abundance between the early (n = 9) and middle (n = 9) or late (n = 9) stages.

Supplementary Table S1. Body weight, dietary composition, and chemical analysis of diets in Japanese Black beef cattle during the Early, Middle, and Late fattening stages

	Stage[1]			
Items	Early	Middle	Late	SEM
Body weight (kg)	439.1[a]	561.8[b]	712.4[c]	12.6
Daily intake amount[2] (kg)				
Concentrate[3]	6.0[a]	7.6[b]	6.1[a]	0.32
Rice straw	2.1[a]	1.1[b]	1.0[b]	0.13
Nutrient adequacy rate[4] (%)				
DM[5]	88.7[a]	96.1[a]	75.4[b]	3.48
TDN[6]	91.2[a]	102.4[a]	74.2[b]	3.85
NDF[7]	43.9[a]	36.8[b]	31.5[c]	0.54

[a,b,c]Different superscripts are significantly ($P < 0.05$) different.

[1]The age of cattle in the Early, Middle, and Late stages were 14, 21, and 29 months, respectively.

[2]Organic matter basis

[3]The concentrate diet composed of barely, steam-flaked corn, wheat bran, and soybean meal and contains 71.2% total digestible nutrient (TDN) and 15.7% crude protein (CP), 72.2% TDN and 13.9% CP, and 72.8% TDN and 12.0% CP during the Early, Middle, and Late stage, respectively.

[4]Nutrient adequacy rate was based on the nutrient requirement of Japanese Feeding Standard for Beef Cattle (NARO, 2009).

[5]DM = dry matter

[6]TDN = total digestible nutrients

[7]NDF = neutral detergent fiber.

Supplementary Table S2. Gene symbols and sequences of the primers used for quantitative real-time PCR.

Gene Symbol	Gene Name		Primer Sequences (5' - 3')	Accession No.
LAMP3	lysosomal-associated membrane protein 3	F	GATCCCTGCAGTGGTGATGTC	NM_001102135
		R	TGCTTGCATGTGACTGTTGTAATG	
ENPP3	ectonucleotide pyrophosphatase/phosphodiesterase 3	F	GACTGCTGCTGGGACTTTGAA	NM_001075923
		R	GTCTCCCCACAGCGAAATTTAT	
BNIPL	BCL2/adenovirus E1B 19kD interacting protein like	F	TCAAGGTGACAGGAAGACTTGTGA	NM_001079621
		R	CTCCTTGATGCCGAGATCCA	
DHTKD1	dehydrogenase E1 and transketolase domain containing 1	F	GTACCGGCTGTGGGAATCG	NM_001205838
		R	GGACGTCATGGGTTGAAGGT	
SLC26A3	solute carrier family 26, member 3	F	AAAAGTTGTGCCGCTGTTCTG	NM_001083676
		R	TGCTGGCAACCAAGATGCTA	
ZNF33B	zinc finger protein 33B	F	AGATGACACCACCCTTATGACAGA	NM_001075810
		R	CCAACTTTTTCACTCTTCTTTCACTTT	
RPS19	ribosomal protein S19	F	ATGTCAACCAGCAGGAGTTCGT	NM_001037467.2
		R	CACCCATTCAGGGACTTTCAG	
POLR2K	polymerase (RNA) II (DNA directed) polypeptide K, 7.0kDa	F	TTTGGAGAGGCCTAGAGATTTCTC	NM_001037623.3
		R	TCATTGGCTGCTGCTTTGG	
RPL21	ribosomal protein L21	F	GCACACTTTGTGAGGACCAATG	NM_001191412.1
		R	TACACCCATCAGGCCATGAA	
GAPDH	glyceraldehyde-3-phosphate dehydrogenase	F	GCCGATGCCCCCATGT	NM_001034034
		R	CAGGAGGATTGCTGACAAATC	
ACTB	actin, beta	F	GGCCGAGCGGAAATCG	NM_173979
		R	GCCATCTCCTGCTCGAAGTC	

RPL27	ribosomal protein L27a	F	GCCCGACGAGAGGCAAA	NM_001034051
		R	AACCGCAGCTTCTGGAAGAA	

F, forward; R, reverse.

Supplementary Table S3. Differentially expressed genes (fold change ≥ 2, $P < 0.05$) uploaded into Ingenuity Pathway Analyses software in the comparisons of the Early and Middle, Middle and Late, and Early and Late stages.

		Fold change			FDR corrected *P*-value		
Gene Symbol	Gene Name	M vs. E[1]	L vs. M	L vs. E	M vs. E	L vs. M	L vs. E
AADAT	aminoadipate aminotransferase	N/A	2.42	N/A	N/A	8.6.E-06	N/A
ABCB10	ATP-binding cassette, sub-family B (MDR/TAP), member 10	N/A	-2.42	N/A	N/A	3.8.E-05	N/A
ABCB6	ATP-binding cassette, sub-family B (MDR/TAP), member 6	-3.02	2.40	N/A	8.5.E-04	4.7.E-04	N/A
ABCC3	ATP-binding cassette, sub-family C (CFTR/MRP), member 3	-2.06	N/A	N/A	2.1.E-04	N/A	N/A
ABCC4	ATP-binding cassette, sub-family C (CFTR/MRP), member 4	4.12	-7.68	N/A	5.9.E-04	5.8.E-06	N/A
ABCD3	ATP-binding cassette, sub-family D (ALD), member 3	-2.88	3.85	N/A	3.1.E-03	1.3.E-04	N/A
ABHD10	abhydrolase domain containing 10	-2.26	2.02	N/A	5.6.E-03	3.6.E-03	N/A
ABHD12	abhydrolase domain containing 12	-2.89	2.88	N/A	1.6.E-04	4.6.E-08	N/A
ABHD15	abhydrolase domain containing 15	N/A	3.05	3.14	N/A	6.5.E-08	3.7.E-06
ABHD16B	abhydrolase domain containing 16B	2.08	-2.55	N/A	2.7.E-03	3.0.E-05	N/A
ABHD3	abhydrolase domain containing 3	N/A	2.05	N/A	N/A	3.2.E-04	N/A
ABHD6	abhydrolase domain containing 6	-4.53	4.58	N/A	1.0.E-03	2.4.E-04	N/A
ACAD10	acyl-CoA dehydrogenase family, member 10	2.29	N/A	N/A	1.1.E-02	N/A	N/A
ACAD8	acyl-CoA dehydrogenase family, member 8	-2.97	2.87	N/A	4.5.E-04	3.3.E-05	N/A
ACADM	acyl-CoA dehydrogenase, C-4 to C-12 straight chain	2.01	-2.17	N/A	9.1.E-04	1.3.E-04	N/A
ACIN1	apoptotic chromatin condensation inducer 1	N/A	-2.25	N/A	N/A	6.3.E-06	N/A
ACOT11	acyl-CoA thioesterase 11	N/A	-2.79	N/A	N/A	3.4.E-06	N/A

ACOT2	acyl-CoA thioesterase 2	-2.92	3.19	N/A	5.6.E-04	1.3.E-04	N/A
ACP5	acid phosphatase 5, tartrate resistant	-2.66	2.99	N/A	7.7.E-04	5.7.E-05	N/A
ACPP	acid phosphatase, prostate	2.17	-3.24	N/A	2.0.E-03	5.9.E-06	N/A
ACSF2	acyl-CoA synthetase family member 2	-3.11	2.49	N/A	5.7.E-04	1.2.E-05	N/A
ACSS2	acyl-CoA synthetase short-chain family member 2	-2.19	2.68	N/A	4.1.E-03	2.7.E-04	N/A
ACTA2	actin, alpha 2, smooth muscle, aorta	N/A	-2.22	N/A	N/A	1.0.E-02	N/A
ACTB	actin, beta	-2.11	N/A	N/A	5.3.E-04	N/A	N/A
ACTL6A	actin-like 6A	N/A	2.18	N/A	N/A	7.6.E-05	N/A
ACTR10	actin-related protein 10 homolog (S. cerevisiae)	-2.03	N/A	N/A	4.5.E-03	N/A	N/A
ACTR1A	ARP1 actin-related protein 1 homolog A, centractin alpha (yeast)	-2.63	2.14	N/A	3.2.E-03	8.3.E-03	N/A
ADAMTS1	ADAM metallopeptidase with thrombospondin type 1 motif, 1	2.20	N/A	N/A	3.3.E-03	N/A	N/A
ADAMTS2	ADAM metallopeptidase with thrombospondin type 1 motif, 2	N/A	-2.45	-2.01	N/A	2.6.E-03	3.3.E-03
ADAMTSL2	ADAMTS-like 2	N/A	-2.06	N/A	N/A	6.4.E-04	N/A
ADCK5	aarF domain containing kinase 5	N/A	-2.25	N/A	N/A	5.5.E-06	N/A
ADIPOR2	adiponectin receptor 2	-3.32	2.67	N/A	4.1.E-03	3.5.E-03	N/A
ADPRHL2	ADP-ribosylhydrolase like 2	-3.57	3.12	N/A	1.4.E-04	2.0.E-06	N/A
AES	amino-terminal enhancer of split	-2.47	2.18	N/A	2.8.E-04	8.4.E-05	N/A
AFF4	AF4/FMR2 family, member 4	2.41	-2.62	N/A	9.2.E-04	3.9.E-05	N/A
AFTPH	aftiphilin	-2.68	2.38	N/A	1.0.E-03	2.9.E-05	N/A
AGAP3	ArfGAP with GTPase domain, ankyrin repeat and PH domain 3	N/A	N/A	-2.11	N/A	N/A	3.2.E-04
AGGF1	angiogenic factor with G patch and FHA domains 1	2.66	-3.21	N/A	6.1.E-04	9.2.E-05	N/A
AGMO	alkylglycerol monooxygenase	2.37	-2.46	N/A	4.4.E-04	1.0.E-05	N/A

AGPS	alkylglycerone phosphate synthase	N/A	-2.45	N/A	N/A	9.1.E-06	N/A
AGTRAP	angiotensin II receptor-associated protein	-3.36	4.58	N/A	5.3.E-04	1.8.E-05	N/A
AHCTF1	AT hook containing transcription factor 1	3.72	-6.30	N/A	6.0.E-04	2.5.E-06	N/A
AHCY	adenosylhomocysteinase	N/A	-3.11	-2.32	N/A	2.1.E-07	8.0.E-06
AHCYL2	adenosylhomocysteinase-like 2	N/A	2.07	N/A	N/A	4.5.E-04	N/A
AHNAK	AHNAK nucleoprotein	N/A	-3.28	N/A	N/A	3.2.E-05	N/A
AJUBA	jub, ajuba homolog (Xenopus laevis)	N/A	-3.47	N/A	N/A	6.5.E-07	N/A
AK3	adenylate kinase 3	N/A	2.35	N/A	N/A	8.3.E-05	N/A
AKAP11	A kinase (PRKA) anchor protein 11	2.57	-2.89	N/A	2.8.E-04	4.3.E-06	N/A
AKR1A1	aldo-keto reductase family 1, member A1 (aldehyde reductase)	-4.21	4.95	N/A	1.2.E-04	2.2.E-07	N/A
AKR7A2	aldo-keto reductase family 7, member A2 (aflatoxin aldehyde reductase)	-3.38	4.42	N/A	2.6.E-04	3.9.E-07	N/A
AKT1	v-akt murine thymoma viral oncogene homolog 1	-2.05	N/A	N/A	4.8.E-04	N/A	N/A
ALAD	aminolevulinate dehydratase	N/A	2.07	N/A	N/A	1.1.E-03	N/A
ALCAM	activated leukocyte cell adhesion molecule	2.36	-2.77	N/A	2.1.E-04	5.7.E-06	N/A
ALDH1A1	aldehyde dehydrogenase 1 family, member A1	-3.01	3.14	N/A	3.0.E-03	1.2.E-03	N/A
ALDH8A1	aldehyde dehydrogenase 8 family, member A1	2.82	-3.41	N/A	2.1.E-04	1.9.E-06	N/A
ALG3	asparagine-linked glycosylation 3, alpha-1,3-mannosyltransferase homolog (S. cerevisiae)	N/A	-2.22	N/A	N/A	5.4.E-05	N/A
AMFR	autocrine motility factor receptor	-2.15	2.72	N/A	2.6.E-03	1.5.E-04	N/A
AMH	anti-Mullerian hormone	N/A	-2.86	N/A	N/A	1.7.E-07	N/A
AMZ2	archaelysin family metallopeptidase 2	-2.63	3.08	N/A	7.6.E-05	8.9.E-08	N/A
ANAPC16	anaphase promoting complex subunit 16	-2.35	N/A	N/A	4.6.E-04	N/A	N/A
ANKRD17	ankyrin repeat domain 17	2.33	-3.26	N/A	2.4.E-03	3.8.E-05	N/A
ANKRD22	ankyrin repeat domain 22	-2.15	N/A	N/A	6.6.E-03	N/A	N/A

ANKRD37	ankyrin repeat domain 37	N/A	2.59	N/A	N/A	3.8.E-05	N/A
ANKRD46	ankyrin repeat domain 46	-2.08	2.19	N/A	3.0.E-04	7.0.E-06	N/A
ANKS3	ankyrin repeat and sterile alpha motif domain containing 3	2.07	-3.04	N/A	1.3.E-03	5.0.E-05	N/A
ANO10	anoctamin 10	-2.35	2.22	N/A	1.2.E-03	1.3.E-04	N/A
ANP32B	acidic (leucine-rich) nuclear phosphoprotein 32 family, member B	2.35	-3.87	N/A	1.1.E-03	3.1.E-06	N/A
ANPEP	alanyl (membrane) aminopeptidase	N/A	-2.11	N/A	N/A	1.1.E-03	N/A
ANXA2	annexin A2	-2.20	N/A	N/A	5.8.E-04	N/A	N/A
AP1S1	adaptor-related protein complex 1, sigma 1 subunit	-2.67	2.34	N/A	7.1.E-04	4.9.E-04	N/A
APBB2	amyloid beta (A4) precursor protein-binding, family B, member 2	2.71	-2.24	N/A	1.3.E-03	3.4.E-04	N/A
APEH	N-acylaminoacyl-peptide hydrolase	-2.66	2.50	N/A	1.3.E-03	3.4.E-04	N/A
APOE	apolipoprotein E	2.02	N/A	N/A	1.0.E-03	N/A	N/A
AQP2	aquaporin 2 (collecting duct)	-3.47	2.71	N/A	3.9.E-04	7.5.E-04	N/A
AQP3	aquaporin 3 (Gill blood group)	-4.92	2.99	N/A	1.2.E-04	3.5.E-05	N/A
AQP8	aquaporin 8	N/A	-2.46	-3.21	N/A	1.2.E-04	3.1.E-05
ARFGEF1	ADP-ribosylation factor guanine nucleotide-exchange factor 1 (brefeldin A-inhibited)	-2.82	2.95	N/A	5.3.E-04	2.3.E-05	N/A
ARFIP1	ADP-ribosylation factor interacting protein 1	-3.39	3.54	N/A	4.6.E-04	1.1.E-06	N/A
ARG1	arginase, liver	-2.52	4.93	N/A	1.1.E-02	8.5.E-05	N/A
ARGLU1	arginine and glutamate rich 1	2.88	-3.39	N/A	6.3.E-04	3.0.E-05	N/A
ARHGAP17	Rho GTPase activating protein 17	N/A	-2.41	N/A	N/A	8.4.E-07	N/A
ARHGAP29	Rho GTPase activating protein 29	2.61	-4.52	N/A	9.6.E-04	1.5.E-05	N/A
ARHGDIB	Rho GDP dissociation inhibitor (GDI) beta	-2.83	N/A	N/A	1.4.E-02	N/A	N/A
ARHGEF16	Rho guanine nucleotide exchange factor (GEF) 16	N/A	-2.03	N/A	N/A	4.0.E-04	N/A
ARHGEF40	Rho guanine nucleotide exchange factor (GEF) 40	N/A	-2.39	N/A	N/A	1.2.E-04	N/A

ARID4A	AT rich interactive domain 4A (RBP1-like)	N/A	-2.23	N/A	N/A	4.7.E-04	N/A
ARID5A	AT rich interactive domain 5A (MRF1-like)	N/A	-2.42	N/A	N/A	2.6.E-05	N/A
ARID5B	AT rich interactive domain 5B (MRF1-like)	3.41	-3.01	N/A	1.3.E-03	1.9.E-04	N/A
ARL4D	ADP-ribosylation factor-like 4D	N/A	N/A	2.22	N/A	N/A	8.3.E-04
ARL5A	ADP-ribosylation factor-like 5A	N/A	2.00	N/A	N/A	1.8.E-03	N/A
ARL6IP1	ADP-ribosylation factor-like 6 interacting protein 1	-2.50	2.70	N/A	2.5.E-03	8.4.E-05	N/A
ARMC5	armadillo repeat containing 5	2.14	-2.09	N/A	1.4.E-03	5.4.E-04	N/A
ARMC7	armadillo repeat containing 7	2.03	-2.84	N/A	5.4.E-04	1.3.E-06	N/A
ARNTL	aryl hydrocarbon receptor nuclear translocator-like	-2.96	2.77	N/A	1.1.E-03	2.1.E-04	N/A
ARPC1B	actin related protein 2/3 complex, subunit 1B, 41kDa	-2.71	N/A	N/A	1.0.E-03	N/A	N/A
ARPC3	actin related protein 2/3 complex, subunit 3, 21kDa	N/A	-2.16	N/A	N/A	1.7.E-06	N/A
ARPC4	actin related protein 2/3 complex, subunit 4, 20kDa	-2.60	2.54	N/A	2.2.E-03	6.5.E-04	N/A
ARPP19	cAMP-regulated phosphoprotein, 19kDa	-2.23	2.26	N/A	4.3.E-03	8.0.E-04	N/A
ARSB	arylsulfatase B	N/A	2.44	N/A	N/A	1.7.E-04	N/A
ART1	ADP-ribosyltransferase 1	N/A	-6.68	-3.90	N/A	1.8.E-06	3.9.E-06
ASCC3	activating signal cointegrator 1 complex subunit 3	N/A	2.05	N/A	N/A	9.7.E-04	N/A
ASIP	agouti signaling protein	3.13	-2.45	N/A	1.1.E-03	5.1.E-04	N/A
ATAD3A	ATPase family, AAA domain containing 3A	-2.09	N/A	N/A	3.9.E-03	N/A	N/A
ATF6B	activating transcription factor 6 beta	2.16	-2.97	N/A	5.8.E-04	5.5.E-08	N/A
ATF7IP	activating transcription factor 7 interacting protein	2.91	-3.13	N/A	3.3.E-04	1.1.E-05	N/A
ATG12	ATG12 autophagy related 12 homolog (S. cerevisiae)	N/A	2.19	N/A	N/A	2.2.E-02	N/A
ATG4D	ATG4 autophagy related 4 homolog D (S. cerevisiae)	-3.13	3.85	N/A	2.9.E-04	5.9.E-06	N/A
ATG9A	ATG9 autophagy related 9 homolog A (S. cerevisiae)	-2.24	3.00	N/A	1.9.E-02	7.9.E-05	N/A
ATN1	atrophin 1	N/A	-3.06	N/A	N/A	1.6.E-03	N/A
ATP11B	ATPase, class VI, type 11B	-2.37	2.02	N/A	4.5.E-04	2.7.E-04	N/A

ATP13A3	ATPase type 13A3	2.04	N/A	N/A	1.0.E-03	N/A	N/A
ATP5B	ATP synthase, H+ transporting, mitochondrial F1 complex, beta polypeptide	-2.15	N/A	N/A	7.6.E-04	N/A	N/A
ATP5C1	ATP synthase, H+ transporting, mitochondrial F1 complex, gamma polypeptide 1	-3.39	2.16	N/A	2.4.E-04	3.9.E-05	N/A
ATP5O	ATP synthase, H+ transporting, mitochondrial F1 complex, O subunit	N/A	2.05	N/A	N/A	4.9.E-05	N/A
ATP6V0B	ATPase, H+ transporting, lysosomal 21kDa, V0 subunit b	-4.10	4.43	N/A	4.8.E-04	5.8.E-06	N/A
ATP6V0D1	ATPase, H+ transporting, lysosomal 38kDa, V0 subunit d1	-3.69	4.30	N/A	1.2.E-03	1.3.E-04	N/A
ATP6V1A	ATPase, H+ transporting, lysosomal 70kDa, V1 subunit A	-4.57	5.53	N/A	2.1.E-04	3.3.E-07	N/A
ATP6V1C2	ATPase, H+ transporting, lysosomal 42kDa, V1 subunit C2	-2.31	4.24	N/A	2.4.E-02	1.3.E-04	N/A
ATP6V1H	ATPase, H+ transporting, lysosomal 50/57kDa, V1 subunit H	-2.97	3.56	N/A	5.6.E-04	1.0.E-06	N/A
ATPAF1	ATP synthase mitochondrial F1 complex assembly factor 1	-3.40	3.94	N/A	4.7.E-04	6.8.E-06	N/A
AUP1	ancient ubiquitous protein 1	-5.15	5.76	N/A	7.6.E-05	1.1.E-06	N/A
AURKA	aurora kinase A	N/A	-2.10	N/A	N/A	4.8.E-03	N/A
AVPI1	arginine vasopressin-induced 1	-2.09	N/A	N/A	5.4.E-04	N/A	N/A
AXIN1	axin 1	-2.25	N/A	N/A	2.2.E-04	N/A	N/A
AZI1	5-azacytidine induced 1	2.38	-2.88	N/A	2.1.E-04	8.8.E-07	N/A
B3GAT3	beta-1,3-glucuronyltransferase 3 (glucuronosyltransferase I)	N/A	-2.90	N/A	N/A	1.5.E-05	N/A
BACE2	beta-site APP-cleaving enzyme 2	N/A	2.44	N/A	N/A	3.7.E-06	N/A
BAIAP2L2	BAI1-associated protein 2-like 2	N/A	2.94	N/A	N/A	1.5.E-04	N/A
BANF1	barrier to autointegration factor 1	-2.20	N/A	N/A	1.9.E-03	N/A	N/A
BARD1	BRCA1 associated RING domain 1	2.42	-2.67	N/A	2.6.E-03	1.3.E-03	N/A
BASP1	brain abundant, membrane attached signal protein 1	2.11	-3.52	N/A	6.3.E-03	1.7.E-04	N/A
BAZ2A	bromodomain adjacent to zinc finger domain, 2A	7.35	-10.24	N/A	2.6.E-04	2.6.E-06	N/A
BCAP31	B-cell receptor-associated protein 31	N/A	-2.11	N/A	N/A	2.4.E-05	N/A

BCO2	beta-carotene oxygenase 2	N/A	2.46	N/A	N/A	1.4.E-04	N/A
BIN1	bridging integrator 1	N/A	-2.70	N/A	N/A	2.1.E-05	N/A
BIN3	bridging integrator 3	-2.50	2.11	N/A	8.5.E-04	2.9.E-04	N/A
BIRC5	baculoviral IAP repeat containing 5	-2.52	N/A	N/A	1.0.E-02	N/A	N/A
BLNK	B-cell linker	-2.28	3.29	N/A	1.3.E-03	7.4.E-06	N/A
BLOC1S6	pallidin homolog (mouse)	2.22	-2.38	N/A	7.4.E-04	4.4.E-06	N/A
BNIP3L	BCL2/adenovirus E1B 19kDa interacting protein 3-like	-2.04	2.56	N/A	1.4.E-02	5.8.E-04	N/A
BNIPL	BCL2/adenovirus E1B 19kD interacting protein like	-7.41	11.16	N/A	2.0.E-04	2.5.E-07	N/A
BOC	Boc homolog (mouse)	2.32	N/A	N/A	3.8.E-04	N/A	N/A
BPNT1	3'(2'), 5'-bisphosphate nucleotidase 1	N/A	2.03	N/A	N/A	1.7.E-03	N/A
BRK1	BRICK1, SCAR/WAVE actin-nucleating complex subunit	N/A	2.14	N/A	N/A	3.0.E-05	N/A
BRP44L	brain protein 44-like	-2.11	N/A	N/A	6.4.E-04	N/A	N/A
BSG	basigin (Ok blood group)	-2.15	2.12	N/A	1.7.E-03	4.9.E-04	N/A
BTN2A1	butyrophilin, subfamily 2, member A1	2.98	-2.76	N/A	3.5.E-04	7.9.E-05	N/A
CA12	carbonic anhydrase XII	-2.11	N/A	N/A	4.4.E-04	N/A	N/A
CABIN1	calcineurin binding protein 1	N/A	-2.29	N/A	N/A	2.5.E-06	N/A
CABP2	calcium binding protein 2	N/A	-2.44	N/A	N/A	4.6.E-04	N/A
CACFD1	calcium channel flower domain containing 1	N/A	-3.51	N/A	N/A	1.8.E-07	N/A
CACNG1	calcium channel, voltage-dependent, gamma subunit 1	2.59	-2.15	N/A	2.5.E-03	3.9.E-03	N/A
CADM3	cell adhesion molecule 3	2.27	N/A	N/A	2.0.E-04	N/A	N/A
CALCOCO2	calcium binding and coiled-coil domain 2	N/A	-2.25	N/A	N/A	1.0.E-05	N/A
CALD1	caldesmon 1	3.02	-3.42	N/A	4.9.E-04	1.5.E-04	N/A
CALML5	calmodulin-like 5	-3.34	3.61	N/A	1.6.E-03	1.8.E-04	N/A
CALY	calcyon neuron-specific vesicular protein	N/A	2.07	N/A	N/A	1.1.E-04	N/A
CAMK1G	calcium/calmodulin-dependent protein kinase IG	2.06	-2.29	N/A	3.0.E-03	3.8.E-05	N/A

CAPNS1	calpain, small subunit 1	-2.55	2.75	N/A	2.1.E-04	1.6.E-06	N/A
CARD9	caspase recruitment domain family, member 9	N/A	-2.04	N/A	N/A	2.7.E-05	N/A
CASP8	caspase 8, apoptosis-related cysteine peptidase	2.93	-3.01	N/A	3.9.E-04	3.0.E-05	N/A
CASP8AP2	caspase 8 associated protein 2	-2.10	N/A	N/A	4.9.E-03	N/A	N/A
CAT	catalase	-4.75	4.70	N/A	7.8.E-05	2.4.E-07	N/A
CAV2	caveolin 2	N/A	-2.14	N/A	N/A	1.6.E-04	N/A
CAV3	caveolin 3	-2.55	N/A	-2.04	7.6.E-03	N/A	5.9.E-03
CBX1	chromobox homolog 1	N/A	-2.58	N/A	N/A	1.4.E-04	N/A
CBX5	chromobox homolog 5	4.09	-4.57	N/A	5.0.E-04	3.1.E-05	N/A
CCAR1	cell division cycle and apoptosis regulator 1	2.45	-3.33	N/A	1.6.E-03	6.2.E-05	N/A
CCBL1	cysteine conjugate-beta lyase, cytoplasmic	-2.90	4.01	N/A	1.8.E-04	5.8.E-08	N/A
CCDC130	coiled-coil domain containing 130	2.48	-2.67	N/A	1.3.E-03	2.1.E-04	N/A
CCDC137	coiled-coil domain containing 137	N/A	-2.05	N/A	N/A	8.4.E-07	N/A
CCDC28A	coiled-coil domain containing 28A	-2.22	2.77	N/A	3.1.E-03	1.0.E-04	N/A
CCDC84	coiled-coil domain containing 84	2.08	N/A	N/A	2.9.E-05	N/A	N/A
CCDC85B	coiled-coil domain containing 85B	N/A	-2.59	N/A	N/A	1.6.E-07	N/A
CCDC91	coiled-coil domain containing 91	N/A	2.01	N/A	N/A	2.0.E-04	N/A
CCHCR1	coiled-coil alpha-helical rod protein 1	N/A	-2.01	N/A	N/A	1.7.E-03	N/A
CCL5	chemokine (C-C motif) ligand 5	-3.15	N/A	-2.43	1.0.E-02	N/A	3.6.E-02
CCNB1	cyclin B1	N/A	-2.02	N/A	N/A	8.0.E-03	N/A
CCND1	cyclin D1	2.14	N/A	N/A	3.1.E-04	N/A	N/A
CCNF	cyclin F	N/A	-2.80	N/A	N/A	6.5.E-04	N/A
CCNI	cyclin I	-2.29	2.29	N/A	2.2.E-03	7.1.E-05	N/A
CCT7	chaperonin containing TCP1, subunit 7 (eta)	-3.11	2.99	N/A	1.2.E-04	3.1.E-05	N/A
CD3EAP	CD3e molecule, epsilon associated protein	2.30	-2.96	N/A	7.3.E-04	5.2.E-05	N/A

CD3G	CD3g molecule, gamma (CD3-TCR complex)	-2.41	3.73	N/A	8.5.E-04	9.0.E-04	N/A
CD48	CD48 molecule	N/A	2.59	N/A	N/A	4.5.E-03	N/A
CD52	CD52 molecule	N/A	-3.17	-2.16	N/A	8.1.E-06	2.6.E-03
CD55	CD55 molecule, decay accelerating factor for complement (Cromer blood group)	N/A	2.32	N/A	N/A	1.0.E-02	N/A
CD9	CD9 molecule	-2.03	2.39	N/A	2.9.E-03	1.2.E-04	N/A
CDA	cytidine deaminase	-3.19	3.11	N/A	2.6.E-04	5.4.E-05	N/A
CDC42EP4	CDC42 effector protein (Rho GTPase binding) 4	N/A	-3.63	-2.66	N/A	1.0.E-05	5.4.E-05
CDC45	cell division cycle 45 homolog (S. cerevisiae)	N/A	-2.10	N/A	N/A	1.1.E-04	N/A
CDCA3	cell division cycle associated 3	N/A	-2.27	N/A	N/A	2.8.E-03	N/A
CDIPT	CDP-diacylglycerol--inositol 3-phosphatidyltransferase	-2.29	2.46	N/A	1.1.E-04	2.9.E-06	N/A
CDK2AP2	cyclin-dependent kinase 2 associated protein 2	-2.59	2.14	N/A	1.8.E-04	2.4.E-05	N/A
CDK5	cyclin-dependent kinase 5	-3.18	3.64	N/A	2.1.E-04	1.9.E-06	N/A
CDK9	cyclin-dependent kinase 9	-2.34	2.01	N/A	7.6.E-05	3.4.E-05	N/A
CDKN2B	cyclin-dependent kinase inhibitor 2B (p15, inhibits CDK4)	N/A	2.31	N/A	N/A	3.7.E-03	N/A
CEACAM1	carcinoembryonic antigen-related cell adhesion molecule 1 (biliary glycoprotein)	N/A	3.11	2.63	N/A	2.8.E-05	1.7.E-03
CEACAM19	carcinoembryonic antigen-related cell adhesion molecule 19	N/A	2.40	N/A	N/A	3.9.E-08	N/A
CEBPZ	CCAAT/enhancer binding protein (C/EBP), zeta	N/A	-2.23	N/A	N/A	3.3.E-05	N/A
CELF4	CUGBP, Elav-like family member 4	N/A	2.15	N/A	N/A	2.3.E-03	N/A
CELSR2	cadherin, EGF LAG seven-pass G-type receptor 2 (flamingo homolog, Drosophila)	3.09	-3.28	N/A	2.6.E-03	2.1.E-04	N/A
CENPW	centromere protein W	N/A	-3.57	-2.15	N/A	1.2.E-04	6.0.E-03
CEP70	centrosomal protein 70kDa	3.41	-4.67	N/A	2.2.E-04	5.8.E-06	N/A
CEPT1	choline/ethanolamine phosphotransferase 1	-2.11	2.23	N/A	5.6.E-04	2.9.E-05	N/A
CERCAM	cerebral endothelial cell adhesion molecule	N/A	-3.48	N/A	N/A	2.2.E-05	N/A

CERS5	ceramide synthase 5	-4.06	4.21	N/A	5.9.E-03	1.4.E-03	N/A
CETN3	centrin, EF-hand protein, 3	2.71	-2.61	N/A	8.7.E-04	1.8.E-04	N/A
CGN	cingulin	2.17	-3.82	N/A	4.1.E-03	1.1.E-05	N/A
CGRRF1	cell growth regulator with ring finger domain 1	N/A	2.79	N/A	N/A	7.9.E-05	N/A
CHCHD3	coiled-coil-helix-coiled-coil-helix domain containing 3	-2.88	2.35	N/A	4.2.E-04	8.6.E-05	N/A
CHCHD4	coiled-coil-helix-coiled-coil-helix domain containing 4	N/A	-2.73	N/A	N/A	4.8.E-08	N/A
CHCHD6	coiled-coil-helix-coiled-coil-helix domain containing 6	N/A	N/A	2.06	N/A	N/A	8.8.E-05
CHD1L	chromodomain helicase DNA binding protein 1-like	-2.13	2.45	N/A	4.1.E-03	4.4.E-04	N/A
CHD7	chromodomain helicase DNA binding protein 7	2.19	N/A	N/A	8.3.E-04	N/A	N/A
CHIC2	cysteine-rich hydrophobic domain 2	-2.06	2.35	N/A	2.6.E-03	1.3.E-04	N/A
CHMP1B	charged multivesicular body protein 1B	-3.28	2.25	N/A	3.9.E-04	4.0.E-04	N/A
CHMP2B	charged multivesicular body protein 2B	-2.21	3.22	N/A	3.0.E-03	2.8.E-04	N/A
CHMP4B	charged multivesicular body protein 4B	-2.09	2.43	N/A	2.4.E-02	2.9.E-04	N/A
CHMP4C	charged multivesicular body protein 4C	N/A	2.33	N/A	N/A	2.0.E-05	N/A
CHMP5	charged multivesicular body protein 5	N/A	2.22	N/A	N/A	6.4.E-04	N/A
CHP1	calcium binding protein P22	N/A	2.08	N/A	N/A	5.7.E-04	N/A
CHP2	calcineurin B homologous protein 2	N/A	-2.01	N/A	N/A	5.3.E-06	N/A
CHRM3	cholinergic receptor, muscarinic 3	2.19	-5.32	-2.42	6.8.E-04	6.5.E-07	1.5.E-04
CHST4	carbohydrate (N-acetylglucosamine 6-O) sulfotransferase 4	N/A	3.29	N/A	N/A	2.9.E-05	N/A
CIAO1	cytosolic iron-sulfur protein assembly 1	N/A	2.06	N/A	N/A	2.4.E-04	N/A
CIDEA	cell death-inducing DFFA-like effector a	-5.70	5.85	N/A	2.6.E-04	2.0.E-05	N/A
CISD2	CDGSH iron sulfur domain 2	-3.65	2.64	N/A	9.0.E-03	1.1.E-02	N/A
CKAP2L	cytoskeleton associated protein 2-like	N/A	-2.38	N/A	N/A	2.3.E-03	N/A
CKMT1B	creatine kinase, mitochondrial 1B	-3.55	2.86	N/A	2.6.E-04	6.4.E-05	N/A
CLCA2	chloride channel accessory 2	N/A	N/A	-2.15	N/A	N/A	1.9.E-02

CLDN3	claudin 3	-4.09	2.74	N/A	1.2.E-03	1.4.E-03	N/A
CLDN4	claudin 4	-2.55	N/A	N/A	8.1.E-04	N/A	N/A
CLDND1	claudin domain containing 1	-2.93	3.54	N/A	3.6.E-03	1.8.E-04	N/A
CLEC14A	C-type lectin domain family 14, member A	2.35	-2.23	N/A	9.2.E-04	2.1.E-04	N/A
CLIC1	chloride intracellular channel 1	-2.02	N/A	N/A	1.6.E-03	N/A	N/A
CLIC3	chloride intracellular channel 3	-2.05	2.75	N/A	1.0.E-03	8.7.E-06	N/A
CLK1	CDC-like kinase 1	N/A	-2.26	N/A	N/A	6.9.E-03	N/A
CLN5	ceroid-lipofuscinosis, neuronal 5	N/A	2.55	N/A	N/A	4.5.E-05	N/A
CLPTM1	cleft lip and palate associated transmembrane protein 1	-4.51	5.71	N/A	3.1.E-04	7.0.E-06	N/A
CLTC	clathrin, heavy chain (Hc)	-2.91	3.01	N/A	2.6.E-03	3.1.E-04	N/A
CNGB1	cyclic nucleotide gated channel beta 1	2.71	-3.80	N/A	5.8.E-04	3.1.E-06	N/A
CNIH1	cornichon homolog (Drosophila)	-2.01	2.77	N/A	3.7.E-04	7.8.E-07	N/A
CNTFR	ciliary neurotrophic factor receptor	2.38	N/A	N/A	2.1.E-03	N/A	N/A
COA3	coiled-coil domain containing 56	N/A	2.30	N/A	N/A	4.7.E-04	N/A
COL17A1	collagen, type XVII, alpha 1	2.58	-2.51	N/A	1.0.E-03	4.9.E-05	N/A
COL1A1	collagen, type I, alpha 1	N/A	-2.13	N/A	N/A	4.9.E-05	N/A
COL27A1	collagen, type XXVII, alpha 1	2.42	N/A	N/A	2.2.E-04	N/A	N/A
COL3A1	collagen, type III, alpha 1	N/A	-2.06	N/A	N/A	4.2.E-03	N/A
COL4A2	collagen, type IV, alpha 2	3.06	-3.17	N/A	7.7.E-04	3.5.E-05	N/A
COL8A1	collagen, type VIII, alpha 1	2.51	-2.27	N/A	1.6.E-02	4.7.E-03	N/A
COMMD10	COMM domain containing 10	N/A	2.45	N/A	N/A	7.6.E-04	N/A
COMMD6	COMM domain containing 6	N/A	2.08	N/A	N/A	3.0.E-04	N/A
COQ10B	coenzyme Q10 homolog B (S. cerevisiae)	2.43	-3.60	N/A	5.3.E-04	3.1.E-06	N/A
COQ9	coenzyme Q9 homolog (S. cerevisiae)	N/A	-2.29	N/A	N/A	1.0.E-03	N/A
CORO2B	coronin, actin binding protein, 2B	2.13	N/A	N/A	5.2.E-03	N/A	N/A

COTL1	coactosin-like 1 (Dictyostelium)	N/A	-2.05	N/A	N/A	1.4.E-03	N/A
COX11	COX11 cytochrome c oxidase assembly homolog (yeast)	N/A	2.33	N/A	N/A	2.7.E-04	N/A
CPNE2	copine II	N/A	N/A	2.03	N/A	N/A	1.9.E-04
CPNE7	copine VII	N/A	-2.37	N/A	N/A	2.3.E-06	N/A
CPNE8	copine VIII	2.02	-2.27	N/A	4.6.E-03	4.3.E-04	N/A
CPSF4	cleavage and polyadenylation specific factor 4, 30kDa	-2.57	N/A	N/A	1.1.E-02	N/A	N/A
CPT1A	carnitine palmitoyltransferase 1A (liver)	2.19	-2.10	N/A	8.6.E-03	5.5.E-03	N/A
CRAT	carnitine O-acetyltransferase	-2.74	2.50	N/A	1.1.E-03	8.0.E-04	N/A
CREB3	cAMP responsive element binding protein 3	N/A	-9.69	-4.89	N/A	1.1.E-08	2.4.E-06
CROCC	ciliary rootlet coiled-coil, rootletin	N/A	-2.06	N/A	N/A	1.1.E-04	N/A
CRYL1	crystallin, lambda 1	N/A	3.12	N/A	N/A	7.3.E-06	N/A
CS	citrate synthase	N/A	-2.06	N/A	N/A	1.2.E-04	N/A
CSNK2A1	casein kinase 2, alpha 1 polypeptide	-2.53	2.79	N/A	2.8.E-04	5.5.E-06	N/A
CSNK2B	casein kinase 2, beta polypeptide	-3.37	3.73	N/A	6.0.E-04	2.5.E-05	N/A
CSPG4	chondroitin sulfate proteoglycan 4	N/A	-2.10	N/A	N/A	4.5.E-05	N/A
CSRP1	cysteine and glycine-rich protein 1	N/A	-2.35	N/A	N/A	4.6.E-05	N/A
CST3	cystatin C	-2.23	2.38	N/A	9.1.E-04	1.5.E-04	N/A
CTDSPL	CTD (carboxy-terminal domain, RNA polymerase II, polypeptide A) small phosphatase-like	-3.95	3.52	N/A	7.9.E-04	1.2.E-04	N/A
CTH	cystathionase (cystathionine gamma-lyase)	-2.24	2.02	N/A	2.1.E-04	1.5.E-03	N/A
CTSB	cathepsin B	-3.78	3.42	N/A	2.2.E-04	1.1.E-05	N/A
CTSD	cathepsin D	-4.07	3.87	N/A	2.9.E-05	6.6.E-07	N/A
CTSH	cathepsin H	-2.02	N/A	N/A	7.7.E-03	N/A	N/A
CTSS	cathepsin S	-2.27	N/A	N/A	2.1.E-02	N/A	N/A
CTSV	cathepsin L2	-3.59	3.51	N/A	2.2.E-04	4.7.E-06	N/A
CTSZ	cathepsin Z	-2.40	2.81	N/A	3.9.E-04	3.7.E-05	N/A

CTTN	cortactin	3.16	-4.74	N/A	5.5.E-04	1.2.E-06	N/A
CUX1	cut-like homeobox 1	-2.67	3.52	N/A	7.6.E-05	1.6.E-07	N/A
CWC22	CWC22 spliceosome-associated protein homolog (S. cerevisiae)	2.50	-3.01	N/A	7.2.E-04	8.0.E-05	N/A
CWC25	CWC25 spliceosome-associated protein homolog (S. cerevisiae)	2.10	-3.33	N/A	3.0.E-03	4.7.E-05	N/A
CWC27	CWC27 spliceosome-associated protein homolog (S. cerevisiae)	3.57	-3.74	N/A	7.5.E-04	5.3.E-05	N/A
CWH43	cell wall biogenesis 43 C-terminal homolog (S. cerevisiae)	N/A	2.25	N/A	N/A	9.4.E-05	N/A
CXADR	coxsackie virus and adenovirus receptor	N/A	-2.85	N/A	N/A	3.0.E-06	N/A
CXCL10	chemokine (C-X-C motif) ligand 10	N/A	3.91	2.22	N/A	6.8.E-05	3.4.E-02
CXCL6	chemokine (C-X-C motif) ligand 6 (granulocyte chemotactic protein 2)	N/A	-2.68	N/A	N/A	4.5.E-02	N/A
CYB5R1	cytochrome b5 reductase 1	-2.10	2.13	N/A	8.2.E-03	1.0.E-03	N/A
CYCS	cytochrome c, somatic	N/A	N/A	-2.44	N/A	N/A	1.3.E-04
CYFIP1	cytoplasmic FMR1 interacting protein 1	-3.18	2.73	N/A	1.0.E-03	3.0.E-04	N/A
CYP1A1	cytochrome P450, subfamily I (aromatic compound-inducible), polypeptide 1	-6.73	N/A	-4.96	1.6.E-03	N/A	2.9.E-03
CYP26A1	cytochrome P450, family 26, subfamily A, polypeptide 1	-2.04	N/A	N/A	2.1.E-02	N/A	N/A
CYP2B6	cytochrome P450 subfamily 2B	-3.22	2.87	N/A	2.7.E-03	8.8.E-03	N/A
CYP2C18	cytochrome P450, family 2, subfamily C, polypeptide 18	N/A	2.03	N/A	N/A	1.8.E-02	N/A
CYP46A1	cytochrome P450, family 46, subfamily A, polypeptide 1	-2.21	8.92	4.03	2.7.E-02	6.9.E-07	6.0.E-04
CYP51A1	cytochrome P450, family 51, subfamily A, polypeptide 1	N/A	2.14	N/A	N/A	5.4.E-04	N/A
DCTN2	dynactin 2 (p50)	-5.25	3.68	N/A	2.0.E-05	6.7.E-07	N/A
DCTN6	dynactin 6	-2.14	2.28	N/A	1.4.E-03	1.8.E-04	N/A
DDAH2	dimethylarginine dimethylaminohydrolase 2	N/A	-3.94	-2.68	N/A	4.0.E-04	9.1.E-04
DDIT3	DNA-damage-inducible transcript 3	2.54	-2.41	N/A	4.1.E-03	6.7.E-04	N/A

DDX27	DEAD (Asp-Glu-Ala-Asp) box polypeptide 27	2.65	-3.10	N/A	9.4.E-04	8.5.E-05	N/A
DDX3X	DEAD (Asp-Glu-Ala-Asp) box polypeptide 3, X-linked	N/A	-4.43	-2.57	N/A	7.9.E-07	1.1.E-04
DDX42	DEAD (Asp-Glu-Ala-Asp) box polypeptide 42	N/A	-2.48	N/A	N/A	1.3.E-03	N/A
DEGS2	degenerative spermatocyte homolog 2, lipid desaturase (Drosophila)	N/A	-2.33	N/A	N/A	3.6.E-07	N/A
DGCR14	DiGeorge syndrome critical region gene 14	2.21	N/A	N/A	5.9.E-03	N/A	N/A
DGKZ	diacylglycerol kinase, zeta	2.46	-2.67	N/A	2.8.E-04	8.3.E-05	N/A
DHPS	deoxyhypusine synthase	-2.89	3.55	N/A	2.8.E-04	8.1.E-07	N/A
DHRS1	dehydrogenase/reductase (SDR family) member 1	-4.85	4.80	N/A	4.5.E-04	9.6.E-06	N/A
DHTKD1	dehydrogenase E1 and transketolase domain containing 1	3.68	-15.76	-4.29	7.6.E-04	7.3.E-08	1.3.E-05
DLG3	discs, large homolog 3 (Drosophila)	2.27	-2.14	N/A	2.0.E-03	7.1.E-04	N/A
DLST	dihydrolipoamide S-succinyltransferase (E2 component of 2-oxo-glutarate complex)	-4.28	4.81	N/A	3.1.E-04	4.1.E-06	N/A
DMAP1	DNA methyltransferase 1 associated protein 1	N/A	-2.07	N/A	N/A	7.1.E-05	N/A
DMRTA2	DMRT-like family A2	N/A	2.05	N/A	N/A	8.1.E-04	N/A
DNAAF2	dynein, axonemal, assembly factor 2	-2.05	N/A	N/A	1.7.E-03	N/A	N/A
DNASE1L3	deoxyribonuclease I-like 3	-4.24	7.49	N/A	1.5.E-03	7.4.E-06	N/A
DNMT3A	DNA (cytosine-5-)-methyltransferase 3 alpha	4.72	-5.24	N/A	5.8.E-04	7.5.E-06	N/A
DOPEY1	dopey family member 1	2.35	-2.29	N/A	9.4.E-04	1.5.E-04	N/A
DPAGT1	dolichyl-phosphate (UDP-N-acetylglucosamine) N-acetylglucosaminephosphotransferase 1 (GlcNAc-1-P transferase)	-4.33	5.96	N/A	3.0.E-04	3.4.E-06	N/A
DPCD	deleted in primary ciliary dyskinesia homolog (mouse)	-2.65	3.69	N/A	2.1.E-03	9.4.E-05	N/A
DPEP3	dipeptidase 3	N/A	-4.99	-6.09	N/A	2.4.E-06	9.4.E-06
DSC2	desmocollin 2	N/A	2.47	N/A	N/A	9.7.E-04	N/A
DTNB	dystrobrevin, beta	2.12	N/A	N/A	2.8.E-04	N/A	N/A

DTX2	deltex homolog 2 (Drosophila)	N/A	2.21	N/A	N/A	7.3.E-06	N/A
DUOX2	dual oxidase 2	N/A	2.03	N/A	N/A	9.2.E-04	N/A
DUOXA2	dual oxidase maturation factor 2	-8.08	5.14	N/A	1.3.E-03	1.9.E-06	N/A
DUSP1	dual specificity phosphatase 1	-2.48	3.25	N/A	1.0.E-03	8.9.E-05	N/A
DUSP12	dual specificity phosphatase 12	-2.02	2.05	N/A	2.4.E-03	5.7.E-04	N/A
DUT	deoxyuridine triphosphatase	N/A	-2.41	N/A	N/A	3.8.E-03	N/A
DYM	dymeclin	N/A	2.48	N/A	N/A	9.8.E-06	N/A
DYNC1I2	dynein, cytoplasmic 1, intermediate chain 2	-2.06	N/A	N/A	6.7.E-04	N/A	N/A
DYNLT1	dynein, light chain, Tctex-type 1	-3.24	3.32	N/A	2.6.E-04	1.8.E-06	N/A
DYRK1B	dual-specificity tyrosine-(Y)-phosphorylation regulated kinase 1B	N/A	-2.35	N/A	N/A	4.2.E-05	N/A
DYRK3	dual-specificity tyrosine-(Y)-phosphorylation regulated kinase 3	-3.57	3.20	N/A	1.1.E-04	1.4.E-07	N/A
EBP	emopamil binding protein (sterol isomerase)	N/A	-2.05	N/A	N/A	2.4.E-06	N/A
EBPL	emopamil binding protein-like	N/A	2.10	N/A	N/A	1.9.E-04	N/A
ECH1	enoyl CoA hydratase 1, peroxisomal	-4.56	4.23	N/A	4.7.E-04	5.8.E-05	N/A
ECHS1	enoyl CoA hydratase, short chain, 1, mitochondrial	-4.78	3.42	N/A	2.3.E-05	2.1.E-07	N/A
ECM1	extracellular matrix protein 1	-2.21	2.54	N/A	1.3.E-03	3.5.E-05	N/A
EEF1A1	eukaryotic translation elongation factor 1 alpha 1	-3.74	3.02	N/A	4.2.E-04	8.3.E-05	N/A
EEF1G	eukaryotic translation elongation factor 1 gamma	-2.88	2.46	N/A	2.1.E-03	1.8.E-03	N/A
EFTUD2	elongation factor Tu GTP binding domain containing 2	2.64	N/A	N/A	8.9.E-03	N/A	N/A
EGFLAM	EGF-like, fibronectin type III and laminin G domains	N/A	-2.12	N/A	N/A	1.5.E-03	N/A
EGLN1	egl nine homolog 1 (C. elegans)	N/A	2.27	N/A	N/A	3.0.E-05	N/A
EHD4	EH-domain containing 4	-2.81	2.41	N/A	5.0.E-05	1.8.E-06	N/A
EIF2B3	eukaryotic translation initiation factor 2B, subunit 3 gamma, 58kDa	N/A	2.16	N/A	N/A	3.1.E-04	N/A

EIF2C2	eukaryotic translation initiation factor 2C, 2	-2.07	2.01	N/A	4.0.E-03	1.0.E-03	N/A
EIF2S2	eukaryotic translation initiation factor 2, subunit 2 beta, 38kDa	-2.41	2.70	N/A	1.6.E-03	2.5.E-04	N/A
EIF3A	eukaryotic translation initiation factor 3, subunit A	N/A	-2.58	N/A	N/A	4.9.E-04	N/A
EIF3B	eukaryotic translation initiation factor 3, subunit B	-2.04	N/A	N/A	6.4.E-03	N/A	N/A
EIF3E	eukaryotic translation initiation factor 3, subunit E	N/A	2.02	N/A	N/A	3.3.E-03	N/A
EIF3F	eukaryotic translation initiation factor 3, subunit F	-2.33	2.91	N/A	1.4.E-03	4.0.E-05	N/A
EIF3G	eukaryotic translation initiation factor 3, subunit G	-2.83	3.44	N/A	2.1.E-04	3.0.E-06	N/A
EIF3J	eukaryotic translation initiation factor 3, subunit J	-2.08	N/A	N/A	2.2.E-03	N/A	N/A
EIF3M	eukaryotic translation initiation factor 3, subunit M	-2.15	2.24	N/A	4.6.E-03	1.3.E-03	N/A
EIF4A1	eukaryotic translation initiation factor 4A1	N/A	-2.09	N/A	N/A	1.7.E-06	N/A
EIF6	eukaryotic translation initiation factor 6	-3.37	2.71	N/A	3.1.E-04	1.5.E-04	N/A
ELF5	E74-like factor 5 (ets domain transcription factor)	-2.15	3.69	N/A	1.2.E-02	8.6.E-06	N/A
ELOVL1	ELOVL fatty acid elongase 1	-4.92	4.86	N/A	9.6.E-05	2.0.E-06	N/A
ELOVL6	ELOVL fatty acid elongase 6	3.40	-4.32	N/A	2.0.E-03	1.3.E-04	N/A
EMC10	chromosome 18 open reading frame, human C19orf63	-2.91	2.91	N/A	1.1.E-04	6.3.E-06	N/A
EMC7	chromosome 10 open reading frame, human C15orf24	N/A	2.10	N/A	N/A	1.1.E-04	N/A
EMD	emerin	-3.40	2.16	N/A	1.1.E-04	6.8.E-05	N/A
ENDOG	endonuclease G	N/A	-2.83	N/A	N/A	5.4.E-07	N/A
ENDOV	endonuclease V	N/A	2.24	N/A	N/A	2.4.E-04	N/A
ENPP3	ectonucleotide pyrophosphatase/phosphodiesterase 3	-6.42	11.25	N/A	3.4.E-04	3.5.E-07	N/A
ENTPD5	ectonucleoside triphosphate diphosphohydrolase 5	2.24	-3.02	N/A	7.1.E-03	3.0.E-04	N/A
EPCAM	epithelial cell adhesion molecule	-2.04	2.76	N/A	1.6.E-03	7.5.E-05	N/A
EPHA1	EPH receptor A1	N/A	N/A	-2.15	N/A	N/A	1.1.E-03
EPHA8	EPH receptor A8	-2.33	2.37	N/A	2.0.E-03	1.2.E-03	N/A
EPHB2	EPH receptor B2	N/A	-2.17	N/A	N/A	3.3.E-05	N/A

EPHB6	EPH receptor B6	N/A	-7.44	-5.07	N/A	1.0.E-07	6.8.E-06
EPHX1	epoxide hydrolase 1, microsomal (xenobiotic)	-3.13	3.24	N/A	2.6.E-04	1.5.E-05	N/A
EPHX2	epoxide hydrolase 2, cytoplasmic	N/A	2.06	N/A	N/A	3.7.E-04	N/A
EPN3	epsin 3	3.75	-2.95	N/A	2.3.E-03	5.2.E-04	N/A
EPRS	glutamyl-prolyl-tRNA synthetase	-2.03	N/A	N/A	1.0.E-03	N/A	N/A
ERLEC1	endoplasmic reticulum lectin 1	-2.07	2.55	N/A	1.4.E-03	1.3.E-05	N/A
ERP44	endoplasmic reticulum protein 44	-2.40	N/A	N/A	6.7.E-04	N/A	N/A
ERRFI1	ERBB receptor feedback inhibitor 1	-2.45	2.76	N/A	1.3.E-02	2.0.E-03	N/A
ESF1	ESF1, nucleolar pre-rRNA processing protein, homolog (S. cerevisiae)	2.10	-4.76	-2.27	1.3.E-03	5.6.E-06	6.5.E-05
ETFDH	electron-transferring-flavoprotein dehydrogenase	-2.28	2.02	N/A	9.4.E-04	8.7.E-04	N/A
ETNPPL	alanine-glyoxylate aminotransferase 2-like 1	-2.81	4.00	N/A	2.0.E-02	2.9.E-03	N/A
EXOC3	exocyst complex component 3	2.32	-2.43	N/A	1.2.E-03	2.5.E-04	N/A
EXOSC10	exosome component 10	N/A	-2.16	N/A	N/A	1.1.E-04	N/A
FABP4	fatty acid binding protein 4, adipocyte	N/A	N/A	3.63	N/A	N/A	1.2.E-02
FADS3	fatty acid desaturase 3	N/A	-2.70	N/A	N/A	3.2.E-06	N/A
FAM212B	chromosome 3 open reading frame, human C1orf183	N/A	-2.78	N/A	N/A	2.2.E-06	N/A
FBF1	Fas (TNFRSF6) binding factor 1	N/A	-3.56	N/A	N/A	4.7.E-06	N/A
FBP1	fructose-1,6-bisphosphatase 1	N/A	2.66	N/A	N/A	9.4.E-05	N/A
FBXO3	F-box protein 3	N/A	2.29	N/A	N/A	7.9.E-04	N/A
FBXO8	F-box protein 8	-2.18	2.48	N/A	1.4.E-03	7.7.E-05	N/A
FBXO9	F-box protein 9	N/A	-2.23	N/A	N/A	1.9.E-04	N/A
FDPS	farnesyl diphosphate synthase	2.04	-2.29	N/A	6.5.E-04	1.5.E-06	N/A
FDX1L	ferredoxin 1-like	N/A	-2.24	N/A	N/A	2.2.E-06	N/A
FEM1A	fem-1 homolog a (C. elegans)	-2.34	N/A	N/A	3.1.E-04	N/A	N/A
FEN1	flap structure-specific endonuclease 1	N/A	-2.35	N/A	N/A	4.5.E-04	N/A

FERMT3	fermitin family member 3	N/A	3.30	4.09	N/A	6.5.E-09	1.9.E-05
FGD5	FYVE, RhoGEF and PH domain containing 5	N/A	-2.10	N/A	N/A	1.0.E-04	N/A
FGF16	fibroblast growth factor 16	5.29	-2.78	N/A	2.4.E-03	3.4.E-03	N/A
FGFBP1	fibroblast growth factor binding protein 1	-2.58	2.35	N/A	6.5.E-04	1.6.E-03	N/A
FGFR1	fibroblast growth factor receptor 1	N/A	-2.07	N/A	N/A	9.9.E-04	N/A
FGFR1OP	FGFR1 oncogene partner	N/A	2.17	N/A	N/A	1.7.E-04	N/A
FHL1	four and a half LIM domains 1	N/A	-2.28	-2.45	N/A	6.8.E-03	4.0.E-02
FIBCD1	fibrinogen C domain containing 1	N/A	-2.24	N/A	N/A	2.3.E-03	N/A
FILIP1	filamin A interacting protein 1	2.78	-3.58	N/A	8.5.E-04	2.6.E-05	N/A
FILIP1L	filamin A interacting protein 1-like	2.50	-4.93	N/A	2.6.E-03	2.7.E-05	N/A
FITM2	fat storage-inducing transmembrane protein 2	-2.24	2.11	N/A	2.1.E-03	7.8.E-04	N/A
FKBP10	FK506 binding protein 10, 65 kDa	3.02	-4.88	N/A	7.3.E-04	4.0.E-05	N/A
FKBP14	FK506 binding protein 14, 22 kDa	2.22	-2.70	N/A	3.4.E-03	1.2.E-04	N/A
FKRP	fukutin related protein	N/A	2.21	N/A	N/A	5.8.E-03	N/A
FLVCR2	feline leukemia virus subgroup C cellular receptor family, member 2	N/A	-2.16	N/A	N/A	8.3.E-06	N/A
FMO5	flavin containing monooxygenase 5	-3.51	4.38	N/A	1.7.E-03	9.8.E-05	N/A
FNDC5	fibronectin type III domain containing 5	4.07	-10.07	-2.47	4.8.E-04	6.3.E-07	8.2.E-05
FOS	FBJ murine osteosarcoma viral oncogene homolog	-3.21	2.14	N/A	7.5.E-04	1.9.E-02	N/A
FOXE1	forkhead box E1 (thyroid transcription factor 2)	-2.06	2.15	N/A	2.8.E-03	7.7.E-04	N/A
FOXP1	forkhead box P1	2.26	N/A	N/A	3.3.E-04	N/A	N/A
FOXS1	forkhead box S1	N/A	-2.49	N/A	N/A	3.3.E-04	N/A
FUT2	fucosyltransferase 2 (secretor status included)	N/A	-2.11	N/A	N/A	2.8.E-07	N/A
FUT4	fucosyltransferase 4 (alpha (1,3) fucosyltransferase, myeloid-specific)	-6.01	9.62	N/A	2.0.E-03	3.6.E-06	N/A
FUT5	fucosyltransferase 5 (alpha (1,3) fucosyltransferase)	N/A	2.34	N/A	N/A	1.1.E-03	N/A

FXR2	fragile X mental retardation, autosomal homolog 2	N/A	-2.64	N/A	N/A	3.3.E-05	N/A
FXYD6	FXYD domain containing ion transport regulator 6	-2.10	N/A	N/A	4.3.E-03	N/A	N/A
GABARAPL1	GABA(A) receptor-associated protein like 1	N/A	2.31	N/A	N/A	3.3.E-04	N/A
GALNT12	UDP-N-acetyl-alpha-D-galactosamine:polypeptide N-acetylgalactosaminyltransferase 12 (GalNAc-T12)	-2.51	3.81	N/A	6.1.E-03	3.0.E-04	N/A
GALNTL4	UDP-N-acetyl-alpha-D-galactosamine:polypeptide N-acetylgalactosaminyltransferase-like 4	N/A	-2.19	N/A	N/A	3.5.E-05	N/A
GAPDH	glyceraldehyde-3-phosphate dehydrogenase	-2.64	2.83	N/A	1.4.E-03	2.1.E-05	N/A
GAPVD1	GTPase activating protein and VPS9 domains 1	-2.48	N/A	N/A	1.1.E-03	N/A	N/A
GATA6	GATA binding protein 6	2.95	-3.08	N/A	5.1.E-04	1.6.E-06	N/A
GATAD2B	GATA zinc finger domain containing 2B	N/A	N/A	2.28	N/A	N/A	1.1.E-03
GATC	glutamyl-tRNA(Gln) amidotransferase, subunit C homolog (bacterial)	-3.36	2.89	N/A	5.2.E-04	6.0.E-05	N/A
GATM	glycine amidinotransferase (L-arginine:glycine amidinotransferase)	-2.70	2.39	N/A	1.4.E-03	1.1.E-04	N/A
GATSL3	GATS protein-like 3	-2.35	2.22	N/A	6.4.E-04	1.4.E-04	N/A
GBA	glucosidase, beta, acid	N/A	2.48	N/A	N/A	6.7.E-04	N/A
GBE1	glucan (1,4-alpha-), branching enzyme 1	N/A	2.34	N/A	N/A	3.5.E-04	N/A
GBP7	guanylate binding protein 7	N/A	N/A	-2.12	N/A	N/A	1.9.E-02
GCH1	GTP cyclohydrolase 1	-3.80	2.70	N/A	2.2.E-03	3.4.E-03	N/A
GCNT3	glucosaminyl (N-acetyl) transferase 3, mucin type	-5.67	5.13	N/A	2.8.E-03	7.3.E-05	N/A
GDPD3	glycerophosphodiester phosphodiesterase domain containing 3	-2.54	3.08	N/A	1.7.E-03	1.5.E-06	N/A
GET4	golgi to ER traffic protein 4 homolog (S. cerevisiae)	N/A	2.10	N/A	N/A	1.7.E-04	N/A
GFOD2	glucose-fructose oxidoreductase domain containing 2	-2.49	N/A	N/A	3.5.E-02	N/A	N/A
GGH	gamma-glutamyl hydrolase (conjugase, folylpolygammaglutamyl hydrolase)	-2.33	3.17	N/A	9.2.E-03	1.8.E-03	N/A

GGNBP2	gametogenetin binding protein 2	N/A	-2.04	N/A	N/A	4.1.E-05	N/A
GGTA1	alpha-galactosyltransferase 1 (glycoprotein)	N/A	2.07	N/A	N/A	2.6.E-04	N/A
GHITM	growth hormone inducible transmembrane protein	-4.90	4.76	N/A	1.8.E-04	3.3.E-06	N/A
GIGYF1	GRB10 interacting GYF protein 1	2.47	-2.06	N/A	5.9.E-04	1.1.E-03	N/A
GJB1	gap junction protein, beta 1, 32kDa	N/A	-2.47	N/A	N/A	6.1.E-06	N/A
GJB6	gap junction protein, beta 6, 30kDa	-2.16	N/A	N/A	7.8.E-03	N/A	N/A
GLRX2	glutaredoxin 2	-2.36	N/A	N/A	2.3.E-03	N/A	N/A
GLRX5	glutaredoxin 5	-2.04	2.09	N/A	8.6.E-04	1.6.E-04	N/A
GLT8D2	glycosyltransferase 8 domain containing 2	N/A	-3.06	N/A	N/A	5.9.E-07	N/A
GLTPD1	glycolipid transfer protein domain containing 1	-2.77	2.78	N/A	2.6.E-04	1.8.E-05	N/A
GLTSCR2	glioma tumor suppressor candidate region gene 2	-2.78	2.29	N/A	1.2.E-04	2.3.E-05	N/A
GMPPB	GDP-mannose pyrophosphorylase B	-5.44	4.19	N/A	3.6.E-04	1.0.E-04	N/A
GNAI1	guanine nucleotide binding protein (G protein), alpha inhibiting activity polypeptide 1	-2.06	2.18	N/A	4.5.E-03	1.2.E-03	N/A
GNB1	guanine nucleotide binding protein (G protein), beta polypeptide 1	-2.43	N/A	N/A	7.9.E-04	N/A	N/A
GNB2	guanine nucleotide binding protein (G protein), beta polypeptide 2	N/A	-2.39	N/A	N/A	1.6.E-07	N/A
GNG5	guanine nucleotide binding protein (G protein), gamma 5	-2.17	2.72	N/A	3.1.E-04	2.6.E-06	N/A
GNG7	guanine nucleotide binding protein (G protein), gamma 7	3.14	-4.96	N/A	1.3.E-03	6.0.E-06	N/A
GNMT	glycine N-methyltransferase	2.95	-2.63	N/A	6.7.E-04	2.0.E-04	N/A
GNS	glucosamine (N-acetyl)-6-sulfatase	-12.71	13.20	N/A	2.6.E-04	1.0.E-06	N/A
GOLIM4	golgi integral membrane protein 4	N/A	2.12	N/A	N/A	4.3.E-04	N/A
GPATCH2	G patch domain containing 2	2.99	-3.07	N/A	8.9.E-04	1.7.E-04	N/A
GPR108	G protein-coupled receptor 108	-2.62	2.68	N/A	1.8.E-04	4.3.E-06	N/A
GPR172A	G protein-coupled receptor 172A	N/A	-2.54	N/A	N/A	5.9.E-06	N/A

GPR89	G protein-coupled receptor 89	N/A	2.05	N/A	N/A	2.7.E-06	N/A
GPRC5A	G protein-coupled receptor, family C, group 5, member A	2.05	-2.15	N/A	3.0.E-02	4.2.E-02	N/A
GPSM2	G-protein signaling modulator 2	-2.15	N/A	N/A	2.6.E-02	N/A	N/A
GPX3	glutathione peroxidase 3 (plasma)	-4.87	6.57	N/A	1.1.E-02	2.6.E-05	N/A
GPX4	glutathione peroxidase 4	-2.45	2.48	N/A	1.0.E-03	5.5.E-05	N/A
GRAMD4	GRAM domain containing 4	2.27	-2.37	N/A	8.1.E-04	5.7.E-05	N/A
GRAP	GRB2-related adaptor protein	N/A	-2.16	N/A	N/A	4.0.E-04	N/A
GRHL3	grainyhead-like 3 (Drosophila)	-3.79	3.11	N/A	9.3.E-04	2.0.E-05	N/A
GRINA	glutamate receptor, ionotropic, N-methyl D-aspartate-associated protein 1 (glutamate binding)	-2.34	2.52	N/A	7.6.E-04	1.1.E-05	N/A
GSDMD	gasdermin D	2.27	-2.91	N/A	2.2.E-04	3.9.E-05	N/A
GSTM3	glutathione S-transferase mu 3 (brain)	N/A	N/A	2.15	N/A	N/A	2.7.E-03
GTF2A2	general transcription factor IIA, 2, 12kDa	N/A	2.04	N/A	N/A	5.7.E-04	N/A
GTF2B	general transcription factor IIB	N/A	2.25	N/A	N/A	3.3.E-04	N/A
GTF2E2	general transcription factor IIE, polypeptide 2, beta 34kDa	-2.35	N/A	N/A	8.4.E-04	N/A	N/A
GTF2I	general transcription factor IIi	2.28	-2.38	N/A	1.1.E-03	4.3.E-05	N/A
GYLTL1B	glycosyltransferase-like 1B	N/A	-2.10	N/A	N/A	3.5.E-06	N/A
GZMM	granzyme M (lymphocyte met-ase 1)	4.86	-4.68	N/A	1.8.E-03	5.6.E-05	N/A
H1F0	H1 histone family, member 0	N/A	-2.64	N/A	N/A	1.5.E-05	N/A
H2B	histone H2B	2.29	-2.70	N/A	1.5.E-03	5.5.E-05	N/A
HACE1	HECT domain and ankyrin repeat containing E3 ubiquitin protein ligase 1	-2.34	2.25	N/A	1.5.E-03	8.1.E-04	N/A
HAGH	hydroxyacylglutathione hydrolase	N/A	2.24	N/A	N/A	2.8.E-06	N/A
HAGHL	hydroxyacylglutathione hydrolase-like	2.24	-3.40	N/A	1.3.E-03	3.4.E-06	N/A
HBZ	hemoglobin, zeta	N/A	-2.07	N/A	N/A	8.3.E-05	N/A
HCRT	hypocretin (orexin) neuropeptide precursor	N/A	N/A	-2.55	N/A	N/A	7.0.E-05

HDAC6	histone deacetylase 6	N/A	-2.03	N/A	N/A	1.2.E-06	N/A
HDDC2	HD domain containing 2	N/A	-2.42	N/A	N/A	4.2.E-06	N/A
HDHD2	haloacid dehalogenase-like hydrolase domain containing 2	N/A	2.53	N/A	N/A	1.4.E-04	N/A
HEATR6	HEAT repeat containing 6	N/A	-2.60	N/A	N/A	3.2.E-06	N/A
HEBP1	heme binding protein 1	-3.93	4.11	N/A	6.8.E-04	2.1.E-05	N/A
HECA	headcase homolog (Drosophila)	2.26	-3.31	N/A	1.3.E-03	6.3.E-07	N/A
HERC3	HECT and RLD domain containing E3 ubiquitin protein ligase 3	2.06	-2.27	N/A	8.9.E-04	4.7.E-05	N/A
HERPUD1	homocysteine-inducible, endoplasmic reticulum stress-inducible, ubiquitin-like domain member 1	-3.17	2.95	N/A	1.8.E-04	3.9.E-06	N/A
HGFAC	HGF activator	2.10	-2.15	N/A	1.1.E-02	4.5.E-03	N/A
HIBADH	3-hydroxyisobutyrate dehydrogenase	N/A	2.46	N/A	N/A	1.6.E-05	N/A
HIGD1B	HIG1 hypoxia inducible domain family, member 1B	2.27	-2.37	N/A	2.4.E-03	2.8.E-04	N/A
HIGD1D	HIG1 domain family, member 1D	-2.20	2.73	N/A	1.7.E-04	2.2.E-06	N/A
HIGD2A	HIG1 hypoxia inducible domain family, member 2A	N/A	2.18	N/A	N/A	2.4.E-06	N/A
HINT3	histidine triad nucleotide binding protein 3	-2.51	3.90	N/A	1.1.E-03	1.1.E-05	N/A
HIST1H1C	histone cluster 1, H1c	-4.31	2.32	N/A	2.0.E-03	4.5.E-02	N/A
HIST1H2AG	histone cluster 1, H2ag	N/A	-2.79	N/A	N/A	1.4.E-03	N/A
HIST2H2AC	histone cluster 2, H2ac	N/A	-2.11	N/A	N/A	3.9.E-03	N/A
HMG20A	high mobility group 20A	2.37	N/A	N/A	1.0.E-03	N/A	N/A
HMG20B	high mobility group 20B	2.49	-3.38	N/A	4.1.E-03	2.4.E-04	N/A
HMGB2	high mobility group box 2	2.77	-4.10	N/A	7.9.E-04	1.8.E-04	N/A
HMGCL	3-hydroxymethyl-3-methylglutaryl-CoA lyase	-2.08	N/A	N/A	3.6.E-03	N/A	N/A
HMGN1	high mobility group nucleosome binding domain 1	N/A	-2.68	N/A	N/A	6.2.E-05	N/A
HMOX2	heme oxygenase (decycling) 2	-2.39	2.20	N/A	5.0.E-04	3.9.E-05	N/A
HNRNPAB	heterogeneous nuclear ribonucleoprotein A/B	-2.51	N/A	N/A	5.9.E-04	N/A	N/A

HNRNPL	heterogeneous nuclear ribonucleoprotein L	2.06	N/A	N/A	7.9.E-04	N/A	N/A
HNRNPU	heterogeneous nuclear ribonucleoprotein U (scaffold attachment factor A)	2.05	-4.21	-2.05	2.7.E-04	6.4.E-08	3.7.E-04
HOXC13	homeobox C13	-2.30	3.10	N/A	1.1.E-02	8.7.E-06	N/A
HOXC6	homeobox C6	-2.02	2.59	N/A	6.7.E-03	1.7.E-04	N/A
HOXC8	homeobox C8	3.73	-4.65	N/A	4.0.E-04	6.5.E-06	N/A
HPRT1	hypoxanthine phosphoribosyltransferase 1	-3.93	3.08	N/A	7.0.E-04	3.0.E-04	N/A
HRASLS	HRAS-like suppressor	N/A	-2.60	N/A	N/A	1.7.E-06	N/A
HSD17B2	hydroxysteroid (17-beta) dehydrogenase 2	N/A	2.47	N/A	N/A	1.1.E-03	N/A
HSDL2	hydroxysteroid dehydrogenase like 2	N/A	2.02	N/A	N/A	2.6.E-03	N/A
HSPA14	heat shock 70kDa protein 14	N/A	2.04	N/A	N/A	1.9.E-03	N/A
HSPA4L	heat shock 70kDa protein 4-like	2.15	-2.13	N/A	7.5.E-04	9.3.E-05	N/A
HSPA9	heat shock 70kDa protein 9 (mortalin)	-2.71	N/A	N/A	1.7.E-04	N/A	N/A
HSPB8	heat shock 22kDa protein 8	-2.22	N/A	N/A	2.9.E-02	N/A	N/A
HSPBP1	HSPA (heat shock 70kDa) binding protein, cytoplasmic cochaperone 1	N/A	-2.09	N/A	N/A	3.7.E-05	N/A
HSPG2	heparan sulfate proteoglycan 2	2.33	-2.74	N/A	7.6.E-04	1.5.E-06	N/A
HTATSF1	HIV-1 Tat specific factor 1	2.90	-4.89	N/A	5.3.E-04	1.3.E-05	N/A
IARS2	isoleucyl-tRNA synthetase 2, mitochondrial	-2.11	2.14	N/A	4.1.E-03	5.8.E-04	N/A
ID2	inhibitor of DNA binding 2, dominant negative helix-loop-helix protein	-2.29	2.95	N/A	1.5.E-02	6.4.E-04	N/A
IDH1	isocitrate dehydrogenase 1 (NADP+), soluble	-2.81	3.07	N/A	2.0.E-03	8.5.E-05	N/A
IDS	iduronate 2-sulfatase	-2.41	2.01	N/A	5.9.E-04	5.2.E-04	N/A
IFI16	interferon, gamma-inducible protein 16	N/A	-2.39	N/A	N/A	8.9.E-04	N/A
IFI35	interferon-induced protein 35	N/A	-3.22	N/A	N/A	4.7.E-06	N/A
IFNGR1	interferon gamma receptor 1	-2.65	2.36	N/A	1.2.E-03	1.5.E-04	N/A

IFNGR2	interferon gamma receptor 2 (interferon gamma transducer 1)	-2.07	N/A	N/A	1.2.E-03	N/A	N/A
IGFBP4	insulin-like growth factor binding protein 4	N/A	-4.26	-4.53	N/A	2.5.E-07	6.9.E-06
IGFBP5	insulin-like growth factor binding protein 5	2.20	-2.01	N/A	2.4.E-03	4.5.E-03	N/A
IGFBP6	insulin-like growth factor binding protein 6	2.48	-2.49	N/A	2.9.E-03	6.9.E-03	N/A
IL10RB	interleukin 10 receptor, beta	-4.00	4.41	N/A	9.1.E-04	6.5.E-05	N/A
IL11RA	interleukin 11 receptor, alpha	N/A	-2.37	N/A	N/A	1.4.E-04	N/A
IL1RL1	interleukin 1 receptor-like 1	2.03	-3.39	N/A	3.0.E-03	2.6.E-05	N/A
IL22RA1	interleukin 22 receptor, alpha 1	-2.04	2.07	N/A	1.9.E-02	1.5.E-03	N/A
IL2RG	interleukin 2 receptor, gamma	2.15	-2.01	N/A	1.2.E-03	2.8.E-06	N/A
IL36A	interleukin 36, alpha	N/A	2.17	N/A	N/A	2.8.E-04	N/A
IL36G	interleukin 36, gamma	-5.32	6.56	N/A	2.7.E-03	6.1.E-06	N/A
IL36RN	interleukin 36 receptor antagonist	-4.97	7.02	N/A	9.1.E-04	3.8.E-07	N/A
ILF3	interleukin enhancer binding factor 3, 90kDa	2.27	-2.19	N/A	1.3.E-03	1.1.E-03	N/A
ILK	integrin-linked kinase	-4.82	3.99	N/A	2.8.E-04	1.8.E-05	N/A
INHBA	inhibin, beta A	2.53	-2.77	N/A	2.5.E-03	5.1.E-05	N/A
INHBC	inhibin, beta C	2.12	-2.14	N/A	2.3.E-03	5.6.E-05	N/A
INSIG1	insulin induced gene 1	N/A	2.20	N/A	N/A	3.5.E-03	N/A
INVS	inversin	-2.52	2.49	N/A	3.6.E-03	1.8.E-03	N/A
IPO5	importin 5	3.00	-8.95	-2.98	1.1.E-02	1.2.E-04	4.9.E-05
IQCC	IQ motif containing C	2.17	-3.59	N/A	4.4.E-04	4.5.E-07	N/A
IQCD	IQ motif containing D	N/A	-3.26	N/A	N/A	1.7.E-05	N/A
IQCE	IQ motif containing E	N/A	-2.16	N/A	N/A	1.6.E-04	N/A
IRF2	interferon regulatory factor 2	2.30	-2.03	N/A	1.2.E-03	3.6.E-04	N/A
IRF3	interferon regulatory factor 3	-2.20	N/A	N/A	2.5.E-04	N/A	N/A
ISCA1	iron-sulfur cluster assembly 1 homolog (S. cerevisiae)	-2.80	3.42	N/A	3.0.E-04	1.3.E-06	N/A

ISG15	ISG15 ubiquitin-like modifier	N/A	-2.42	-2.86	N/A	2.0.E-02	4.0.E-02
ISY1	ISY1 splicing factor homolog (S. cerevisiae)	N/A	-2.00	N/A	N/A	5.5.E-05	N/A
ITFG1	integrin alpha FG-GAP repeat containing 1	N/A	2.16	N/A	N/A	4.8.E-05	N/A
ITFG3	integrin alpha FG-GAP repeat containing 3	N/A	-3.73	N/A	N/A	7.7.E-05	N/A
ITGAV	integrin, alpha V (vitronectin receptor, alpha polypeptide, antigen CD51)	-2.64	4.49	N/A	5.3.E-04	7.8.E-05	N/A
ITGB1BP1	integrin beta 1 binding protein 1	N/A	2.12	N/A	N/A	1.1.E-06	N/A
ITM2B	integral membrane protein 2B	-2.19	N/A	N/A	1.6.E-03	N/A	N/A
ITPRIPL1	inositol 1,4,5-trisphosphate receptor interacting protein-like 1	N/A	-2.05	N/A	N/A	2.6.E-06	N/A
IVL	involucrin	-3.80	5.26	N/A	5.6.E-04	5.3.E-08	N/A
JAK1	Janus kinase 1	-9.51	10.12	N/A	1.3.E-03	7.3.E-05	N/A
JDP2	Jun dimerization protein 2	N/A	-2.04	N/A	N/A	1.9.E-03	N/A
JMJD7	jumonji domain containing 7	N/A	-2.93	N/A	N/A	3.1.E-08	N/A
JUN	jun proto-oncogene	N/A	-2.18	N/A	N/A	1.4.E-03	N/A
KANK2	KN motif and ankyrin repeat domains 2	N/A	-4.82	-4.79	N/A	1.1.E-07	7.8.E-06
KARS	lysyl-tRNA synthetase	-4.85	5.05	N/A	2.8.E-04	5.9.E-06	N/A
KAT7	K(lysine) acetyltransferase 7	-4.63	5.29	N/A	2.6.E-03	5.2.E-04	N/A
KAT8	K(lysine) acetyltransferase 8	-2.85	2.69	N/A	5.7.E-04	2.1.E-05	N/A
KCMF1	potassium channel modulatory factor 1	-2.70	2.75	N/A	5.3.E-04	1.1.E-05	N/A
KCNIP3	Kv channel interacting protein 3, calsenilin	-3.31	3.31	N/A	4.5.E-04	5.9.E-05	N/A
KCNJ9	potassium inwardly-rectifying channel, subfamily J, member 9	N/A	2.46	N/A	N/A	1.2.E-06	N/A
KCNK15	potassium channel, subfamily K, member 15	N/A	6.80	13.24	N/A	1.1.E-08	1.5.E-06
KCNK7	potassium channel, subfamily K, member 7	N/A	2.04	N/A	N/A	6.4.E-04	N/A
KCNQ3	potassium voltage-gated channel, KQT-like subfamily, member 3	N/A	-7.49	-3.82	N/A	1.6.E-09	1.0.E-05

KCTD1	potassium channel tetramerisation domain containing 1	-2.40	2.66	N/A	1.7.E-03	1.9.E-04	N/A
KCTD5	potassium channel tetramerisation domain containing 5	-2.05	2.18	N/A	1.8.E-03	9.7.E-05	N/A
KDM3A	lysine (K)-specific demethylase 3A	-2.90	3.74	N/A	2.2.E-03	2.3.E-04	N/A
KDM8	jumonji domain containing 5	N/A	N/A	2.03	N/A	N/A	3.6.E-05
KIF21A	kinesin family member 21A	-2.31	2.14	N/A	6.3.E-04	1.2.E-04	N/A
KIF2C	kinesin family member 2C	N/A	-2.09	N/A	N/A	1.5.E-04	N/A
KIFAP3	kinesin-associated protein 3	N/A	2.16	N/A	N/A	8.3.E-05	N/A
KLC3	kinesin light chain 3	N/A	2.22	N/A	N/A	1.3.E-04	N/A
KLF4	Kruppel-like factor 4 (gut)	-3.04	3.68	N/A	1.5.E-03	1.0.E-04	N/A
KLHL20	kelch-like 20 (Drosophila)	2.06	N/A	N/A	4.2.E-03	N/A	N/A
KRT14	keratin 14	-2.08	N/A	-2.28	1.1.E-03	N/A	1.6.E-04
KRT19	keratin 19	2.30	-2.30	N/A	3.4.E-04	9.5.E-08	N/A
KRT36	keratin 36	N/A	N/A	3.01	N/A	N/A	2.2.E-02
KRT6A	keratin 6A	-2.31	2.73	N/A	1.6.E-02	4.9.E-03	N/A
KRT85	keratin 85	N/A	-3.08	-2.34	N/A	4.2.E-07	1.5.E-04
KRT86	keratin 86	N/A	N/A	-2.44	N/A	N/A	9.3.E-04
KRTAP4-7	keratin associated protein 4-7	N/A	-2.04	N/A	N/A	4.6.E-05	N/A
KTN1	kinectin 1 (kinesin receptor)	2.73	-3.61	N/A	6.4.E-04	3.4.E-05	N/A
LACTB2	lactamase, beta 2	-2.44	2.68	N/A	4.8.E-03	1.0.E-03	N/A
LAMP3	lysosomal-associated membrane protein 3	-5.16	11.78	2.28	1.5.E-03	6.8.E-08	1.0.E-02
LANCL2	LanC lantibiotic synthetase component C-like 2 (bacterial)	2.60	-3.83	N/A	3.4.E-04	3.9.E-10	N/A
LANCL3	LanC lantibiotic synthetase component C-like 3 (bacterial)	N/A	2.16	N/A	N/A	1.2.E-02	N/A
LAPTM5	lysosomal protein transmembrane 5	N/A	-2.11	N/A	N/A	4.1.E-04	N/A
LARP4	La ribonucleoprotein domain family, member 4	N/A	-2.64	N/A	N/A	4.2.E-04	N/A
LAYN	layilin	N/A	-2.73	N/A	N/A	2.8.E-04	N/A

LBP	lipopolysaccharide binding protein	-2.84	2.53	N/A	4.7.E-02	1.2.E-03	N/A
LCAT	lecithin-cholesterol acyltransferase	N/A	-2.04	N/A	N/A	6.5.E-05	N/A
LCOR	ligand dependent nuclear receptor corepressor	2.07	-2.01	N/A	5.9.E-04	1.5.E-04	N/A
LCP1	lymphocyte cytosolic protein 1 (L-plastin)	N/A	-2.32	N/A	N/A	1.3.E-05	N/A
LENG1	leukocyte receptor cluster (LRC) member 1	N/A	-2.03	N/A	N/A	1.5.E-05	N/A
LEO1	Leo1, Paf1/RNA polymerase II complex component, homolog (S. cerevisiae)	N/A	-2.31	N/A	N/A	5.4.E-05	N/A
LEPRE1	leucine proline-enriched proteoglycan (leprecan) 1	N/A	-2.56	N/A	N/A	8.3.E-05	N/A
LFNG	LFNG O-fucosylpeptide 3-beta-N-acetylglucosaminyltransferase	N/A	-2.10	N/A	N/A	1.3.E-04	N/A
LGALS3	lectin, galactoside-binding, soluble, 3	-2.30	4.42	N/A	1.2.E-02	2.1.E-05	N/A
LGALSL	lectin, galactoside-binding-like	N/A	2.03	N/A	N/A	4.3.E-05	N/A
LGMN	legumain	N/A	2.33	N/A	N/A	5.2.E-05	N/A
LGR6	leucine-rich repeat containing G protein-coupled receptor 6	N/A	2.40	2.18	N/A	4.9.E-06	8.8.E-05
LIMK2	LIM domain kinase 2	N/A	2.21	N/A	N/A	6.9.E-06	N/A
LIMS2	LIM and senescent cell antigen-like domains 2	N/A	-2.33	N/A	N/A	4.5.E-04	N/A
LIN7A	lin-7 homolog A (C. elegans)	N/A	-3.07	-3.50	N/A	2.3.E-04	6.0.E-04
LIPH	lipase, member H	N/A	2.14	N/A	N/A	1.9.E-03	N/A
LMNB1	lamin B1	N/A	-2.26	N/A	N/A	7.4.E-04	N/A
LPAR6	lysophosphatidic acid receptor 6	-2.13	2.24	N/A	2.5.E-03	1.3.E-04	N/A
LPCAT4	lysophosphatidylcholine acyltransferase 4	N/A	2.63	N/A	N/A	1.4.E-05	N/A
LRCH4	leucine-rich repeats and calponin homology (CH) domain containing 4	N/A	-3.79	-2.21	N/A	1.1.E-06	5.7.E-04
LRFN2	leucine rich repeat and fibronectin type III domain containing 2	2.72	-15.84	-5.82	1.0.E-03	2.0.E-10	4.4.E-05
LRP3	low density lipoprotein receptor-related protein 3	N/A	-2.22	N/A	N/A	9.9.E-06	N/A
LRRC30	leucine rich repeat containing 30	N/A	-4.64	-2.65	N/A	6.1.E-09	2.3.E-05

LRRC3B	leucine rich repeat containing 3B	N/A	3.17	N/A	N/A	8.8.E-07	N/A
LRRC51	leucine-rich repeat-containing protein 51	-2.61	3.38	N/A	5.7.E-04	6.2.E-06	N/A
LRRC8E	leucine rich repeat containing 8 family, member E	-2.55	2.34	N/A	3.1.E-04	1.1.E-05	N/A
LSM12	LSM12 homolog (S. cerevisiae)	-2.21	2.17	N/A	4.9.E-03	2.1.E-03	N/A
LTB4R2	leukotriene B4 receptor 2	N/A	-4.19	-2.28	N/A	1.1.E-04	1.2.E-04
LTBP2	latent transforming growth factor beta binding protein 2	3.01	-4.87	N/A	5.4.E-04	7.3.E-06	N/A
LTC4S	leukotriene C4 synthase	N/A	-2.05	N/A	N/A	6.6.E-04	N/A
LUC7L2	LUC7-like 2 (S. cerevisiae)	-2.92	3.29	N/A	4.9.E-03	1.1.E-03	N/A
LUM	lumican	-2.08	2.71	N/A	1.2.E-03	1.1.E-05	N/A
LUZP1	leucine zipper protein 1	N/A	-2.26	N/A	N/A	3.1.E-04	N/A
LY6G6C	lymphocyte antigen 6 complex, locus G6C	N/A	2.71	N/A	N/A	7.7.E-05	N/A
LYNX1	Ly6/neurotoxin 1	N/A	2.05	N/A	N/A	5.1.E-06	N/A
LYPD5	LY6/PLAUR domain containing 5	-6.13	8.32	N/A	3.1.E-04	9.9.E-09	N/A
LYRM5	LYR motif containing 5	-2.87	4.10	N/A	6.9.E-04	4.6.E-06	N/A
M6PR	mannose-6-phosphate receptor (cation dependent)	-2.93	3.03	N/A	2.3.E-03	1.9.E-04	N/A
MACF1	microtubule-actin crosslinking factor 1	2.09	-2.73	N/A	1.1.E-02	2.6.E-04	N/A
MAFB	v-maf avian musculoaponeurotic fibrosarcoma oncogene homolog B	N/A	2.42	N/A	N/A	3.3.E-04	N/A
MAGED2	melanoma antigen family D, 2	N/A	2.04	N/A	N/A	1.9.E-02	N/A
MAL	mal, T-cell differentiation protein	-2.11	N/A	N/A	2.8.E-03	N/A	N/A
MANBAL	mannosidase, beta A, lysosomal-like	-2.60	3.13	N/A	6.4.E-04	2.9.E-05	N/A
MAOB	monoamine oxidase B	-2.67	4.09	N/A	1.9.E-03	1.9.E-04	N/A
MAP2K1	mitogen-activated protein kinase kinase 1	-3.09	2.81	N/A	1.1.E-04	8.1.E-08	N/A
MAP2K5	mitogen-activated protein kinase kinase 5	N/A	2.08	N/A	N/A	6.8.E-04	N/A
MAP3K4	mitogen-activated protein kinase kinase kinase 4	N/A	-2.20	N/A	N/A	2.3.E-05	N/A
MAPK12	mitogen-activated protein kinase 12	N/A	-2.05	N/A	N/A	2.4.E-04	N/A

MAPK6	mitogen-activated protein kinase 6	3.16	-5.06	N/A	6.0.E-04	4.3.E-06	N/A
MARVELD1	MARVEL domain containing 1	N/A	-2.05	N/A	N/A	8.6.E-03	N/A
MAT2A	methionine adenosyltransferase II, alpha	-2.70	N/A	N/A	7.8.E-03	N/A	N/A
MATR3	matrin 3	2.27	-3.06	N/A	4.8.E-04	1.1.E-05	N/A
MBD1	methyl-CpG binding domain protein 1	N/A	-2.36	N/A	N/A	3.5.E-05	N/A
MBNL2	muscleblind-like 2 (Drosophila)	-2.05	N/A	N/A	4.5.E-04	N/A	N/A
MBOAT7	membrane bound O-acyltransferase domain containing 7	-2.05	N/A	N/A	6.4.E-04	N/A	N/A
MCAM	melanoma cell adhesion molecule	N/A	-5.12	-5.53	N/A	5.6.E-07	4.9.E-06
MCEE	methylmalonyl CoA epimerase	N/A	2.16	N/A	N/A	3.1.E-03	N/A
MCM10	minichromosome maintenance complex component 10	2.40	-2.58	N/A	9.1.E-04	2.9.E-04	N/A
MCM3	minichromosome maintenance complex component 3	N/A	3.15	N/A	N/A	2.7.E-06	N/A
MDFIC	MyoD family inhibitor domain containing	N/A	2.01	N/A	N/A	1.6.E-04	N/A
MDM2	Mdm2, p53 E3 ubiquitin protein ligase homolog (mouse)	N/A	-3.39	-2.21	N/A	5.4.E-06	3.3.E-04
MDN1	MDN1, midasin homolog (yeast)	-2.00	N/A	N/A	1.2.E-02	N/A	N/A
MECOM	MDS1 and EVI1 complex locus	2.03	N/A	N/A	2.1.E-04	N/A	N/A
MECR	mitochondrial trans-2-enoyl-CoA reductase	2.65	-2.76	N/A	2.0.E-03	1.1.E-04	N/A
MED6	mediator complex subunit 6	-2.19	2.13	N/A	2.8.E-04	4.5.E-06	N/A
MEGF8	multiple EGF-like-domains 8	N/A	-2.24	N/A	N/A	1.7.E-05	N/A
MEOX1	mesenchyme homeobox 1	2.05	-2.72	N/A	2.2.E-02	4.1.E-03	N/A
MEPCE	methylphosphate capping enzyme	N/A	2.24	N/A	N/A	1.9.E-04	N/A
METRNL	meteorin, glial cell differentiation regulator-like	-2.19	2.27	N/A	1.4.E-03	2.3.E-05	N/A
METTL17	methyltransferase like 17	2.06	-2.06	N/A	9.4.E-04	1.5.E-04	N/A
METTL21A	methyltransferase like 21A	2.38	N/A	N/A	2.3.E-03	N/A	N/A
MFGE8	milk fat globule-EGF factor 8 protein	-4.64	6.47	N/A	6.7.E-04	4.2.E-07	N/A
MFN1	mitofusin 1	N/A	-2.34	N/A	N/A	5.8.E-05	N/A

MFSD12	major facilitator superfamily domain containing 12	-3.19	4.41	N/A	3.3.E-03	5.8.E-06	N/A
MFSD5	major facilitator superfamily domain containing 5	-6.39	7.01	N/A	4.5.E-04	3.2.E-05	N/A
MGLL	monoglyceride lipase	N/A	-2.20	N/A	N/A	2.1.E-04	N/A
MGST1	microsomal glutathione S-transferase 1	N/A	2.21	N/A	N/A	2.8.E-03	N/A
MICALL2	MICAL-like 2	N/A	-2.26	N/A	N/A	1.2.E-05	N/A
MIER1	mesoderm induction early response 1 homolog (Xenopus laevis)	2.00	-3.32	N/A	2.4.E-03	6.9.E-06	N/A
MIF4GD	MIF4G domain containing	-5.31	5.92	N/A	5.4.E-04	3.5.E-05	N/A
MKL1	megakaryoblastic leukemia (translocation) 1	N/A	-2.01	N/A	N/A	3.1.E-05	N/A
MKRN1	makorin ring finger protein 1	N/A	2.47	N/A	N/A	1.1.E-04	N/A
MLEC	malectin	2.35	-2.30	N/A	6.9.E-03	2.0.E-03	N/A
MNT	MAX binding protein	3.31	-6.18	N/A	1.0.E-02	1.1.E-04	N/A
MOB2	MOB kinase activator 2	-2.20	2.63	N/A	2.4.E-03	6.2.E-05	N/A
MOCS3	molybdenum cofactor synthesis 3	N/A	-4.56	-2.49	N/A	5.7.E-06	3.8.E-05
MOSPD3	motile sperm domain containing 3	-2.76	2.93	N/A	4.9.E-03	8.5.E-04	N/A
MOV10	Mov10, Moloney leukemia virus 10, homolog (mouse)	N/A	-2.11	N/A	N/A	3.0.E-05	N/A
MPHOSPH6	M-phase phosphoprotein 6	N/A	2.00	N/A	N/A	1.4.E-03	N/A
MPHOSPH8	M-phase phosphoprotein 8	2.42	-2.44	N/A	4.5.E-04	1.5.E-04	N/A
MPP1	membrane protein, palmitoylated 1, 55kDa	-2.08	N/A	N/A	9.5.E-03	N/A	N/A
MPP3	membrane protein, palmitoylated 3 (MAGUK p55 subfamily member 3)	N/A	-4.99	-2.91	N/A	5.8.E-10	3.9.E-06
MPV17	MpV17 mitochondrial inner membrane protein	-2.67	2.84	N/A	1.3.E-03	2.8.E-04	N/A
MPZL2	myelin protein zero-like 2	N/A	2.35	N/A	N/A	2.1.E-03	N/A
MRC2	mannose receptor, C type 2	N/A	-2.13	N/A	N/A	6.5.E-05	N/A
MRPL1	mitochondrial ribosomal protein L1	-2.06	N/A	N/A	2.0.E-05	N/A	N/A
MRPL11	mitochondrial ribosomal protein L11	N/A	2.02	N/A	N/A	6.4.E-06	N/A

MRPL24	mitochondrial ribosomal protein L24	N/A	2.16	N/A	N/A	1.2.E-04	N/A
MRPL28	mitochondrial ribosomal protein L28	-2.81	2.85	N/A	1.8.E-04	1.1.E-06	N/A
MRPL38	mitochondrial ribosomal protein L38	N/A	-2.88	-2.06	N/A	2.5.E-07	1.1.E-05
MRPL57	mitochondrial ribosomal protein 63	N/A	2.30	N/A	N/A	7.5.E-05	N/A
MRPS21	mitochondrial ribosomal protein S21	N/A	2.08	N/A	N/A	5.0.E-07	N/A
MRS2	MRS2 magnesium homeostasis factor homolog (S. cerevisiae)	2.43	-3.43	N/A	6.8.E-04	1.1.E-06	N/A
MSMO1	methylsterol monooxygenase 1	N/A	2.01	N/A	N/A	2.9.E-04	N/A
MSN	moesin	2.18	-2.05	N/A	5.3.E-04	1.9.E-04	N/A
MSTO1	misato homolog 1 (Drosophila)	N/A	-2.13	N/A	N/A	1.2.E-05	N/A
MTCH1	mitochondrial carrier 1	-2.60	N/A	N/A	5.5.E-04	N/A	N/A
MTDH	metadherin	-2.73	3.08	N/A	5.3.E-03	5.4.E-04	N/A
MTHFSD	methenyltetrahydrofolate synthetase domain containing	N/A	-2.08	N/A	N/A	2.0.E-05	N/A
MTMR2	myotubularin related protein 2	4.52	-6.94	N/A	1.1.E-03	2.8.E-05	N/A
MTMR6	myotubularin related protein 6	N/A	2.04	N/A	N/A	4.0.E-03	N/A
MTPN	myotrophin	N/A	-2.54	N/A	N/A	2.5.E-07	N/A
MTRR	5-methyltetrahydrofolate-homocysteine methyltransferase reductase	N/A	-2.59	N/A	N/A	6.6.E-05	N/A
MTSS1L	metastasis suppressor 1-like	N/A	N/A	2.91	N/A	N/A	8.0.E-06
MTTP	microsomal triglyceride transfer protein	N/A	-2.19	N/A	N/A	1.2.E-04	N/A
MTX1	metaxin 1	-2.98	2.86	N/A	5.0.E-04	1.3.E-04	N/A
MTX2	metaxin 2	N/A	2.85	N/A	N/A	3.1.E-06	N/A
MXRA8	matrix-remodelling associated 8	2.49	-2.03	N/A	2.6.E-03	6.1.E-03	N/A
MYADM	myeloid-associated differentiation marker	N/A	-2.52	N/A	N/A	8.9.E-04	N/A
MYL12B	myosin, light chain 12B, regulatory	N/A	2.14	N/A	N/A	1.6.E-04	N/A

MYL6B	myosin, light chain 6B, alkali, smooth muscle and non-muscle	2.02	N/A	N/A	2.3.E-03	N/A	N/A
MYLIP	myosin regulatory light chain interacting protein	-2.03	2.50	N/A	1.4.E-02	1.6.E-03	N/A
MYLK	myosin light chain kinase	N/A	-2.19	N/A	N/A	3.1.E-03	N/A
MYLK4	myosin light chain kinase family, member 4	2.49	-2.42	N/A	1.3.E-03	2.3.E-05	N/A
MYO6	myosin VI	3.37	-6.18	N/A	4.6.E-04	3.2.E-07	N/A
MZB1	marginal zone B and B1 cell-specific protein	N/A	-2.06	N/A	N/A	1.5.E-04	N/A
N4BP2L2	NEDD4 binding protein 2-like 2	N/A	-2.14	N/A	N/A	1.8.E-05	N/A
NAA10	N(alpha)-acetyltransferase 10, NatA catalytic subunit	N/A	2.11	N/A	N/A	2.4.E-04	N/A
NAA15	N(alpha)-acetyltransferase 15, NatA auxiliary subunit	-2.26	2.37	N/A	5.8.E-04	2.8.E-05	N/A
NAA30	N(alpha)-acetyltransferase 30, NatC catalytic subunit	N/A	2.09	N/A	N/A	8.3.E-03	N/A
NAB2	NGFI-A binding protein 2 (EGR1 binding protein 2)	2.19	-2.75	N/A	1.3.E-03	2.8.E-07	N/A
NADK	NAD kinase	-4.25	4.15	N/A	1.6.E-06	1.2.E-09	N/A
NAP1L1	nucleosome assembly protein 1-like 1	2.90	-4.55	N/A	1.8.E-04	1.2.E-05	N/A
NARG2	NMDA receptor regulated 2	2.48	-2.78	N/A	1.0.E-03	1.1.E-04	N/A
NASP	nuclear autoantigenic sperm protein (histone-binding)	N/A	-2.23	N/A	N/A	5.6.E-05	N/A
NBN	nibrin	3.09	-7.87	-2.55	3.5.E-03	5.6.E-05	8.0.E-05
NCAPG	non-SMC condensin I complex, subunit G	N/A	-2.52	N/A	N/A	1.8.E-03	N/A
NCLN	nicalin	N/A	2.05	N/A	N/A	6.4.E-06	N/A
ND6	NADH dehydrogenase subunit 6	5.84	-11.88	-2.04	9.4.E-05	3.5.E-06	6.1.E-03
NDFIP2	Nedd4 family interacting protein 2	-2.18	N/A	N/A	1.3.E-03	N/A	N/A
NDOR1	NADPH dependent diflavin oxidoreductase 1	N/A	-4.00	-2.24	N/A	6.8.E-06	8.0.E-06
NDUFA6	NADH dehydrogenase (ubiquinone) 1 alpha subcomplex, 6, 14kDa	N/A	-4.22	-2.60	N/A	2.3.E-08	2.0.E-05
NDUFA9	NADH dehydrogenase (ubiquinone) 1 alpha subcomplex, 9, 39kDa	-2.06	N/A	N/A	4.3.E-04	N/A	N/A

NDUFAB1	NADH dehydrogenase (ubiquinone) 1, alpha/beta subcomplex, 1, 8kDa	N/A	2.02	N/A	N/A	3.4.E-04	N/A
NDUFAF2	NADH dehydrogenase (ubiquinone) 1 alpha subcomplex, assembly factor 2	N/A	-2.21	N/A	N/A	1.4.E-04	N/A
NDUFS1	NADH dehydrogenase (ubiquinone) Fe-S protein 1, 75kDa (NADH-coenzyme Q reductase)	-3.20	3.07	N/A	2.9.E-05	1.9.E-08	N/A
NDUFS6	NADH dehydrogenase (ubiquinone) Fe-S protein 6, 13kDa (NADH-coenzyme Q reductase)	-2.22	N/A	N/A	2.4.E-03	N/A	N/A
NDUFV3	NADH dehydrogenase (ubiquinone) flavoprotein 3, 10kDa	-2.06	2.18	N/A	7.7.E-03	3.1.E-03	N/A
NECAB2	N-terminal EF-hand calcium binding protein 2	2.19	-2.89	N/A	7.2.E-04	9.4.E-07	N/A
NECAP2	NECAP endocytosis associated 2	-2.33	2.15	N/A	2.8.E-04	2.8.E-05	N/A
NES	nestin	N/A	-2.45	N/A	N/A	3.1.E-05	N/A
NFAT5	nuclear factor of activated T-cells 5, tonicity-responsive	2.08	-2.49	N/A	4.0.E-04	1.0.E-06	N/A
NFIL3	nuclear factor, interleukin 3 regulated	-2.62	2.96	N/A	2.4.E-03	1.7.E-04	N/A
NFU1	NFU1 iron-sulfur cluster scaffold homolog (S. cerevisiae)	N/A	2.59	N/A	N/A	5.9.E-04	N/A
NFXL1	nuclear transcription factor, X-box binding-like 1	2.53	-3.79	N/A	5.9.E-04	7.1.E-06	N/A
NGLY1	N-glycanase 1	-2.06	2.51	N/A	6.7.E-03	8.2.E-04	N/A
NIPA2	non imprinted in Prader-Willi/Angelman syndrome 2	-4.39	5.20	N/A	6.3.E-03	1.1.E-03	N/A
NIPSNAP1	nipsnap homolog 1 (C. elegans)	N/A	-2.23	N/A	N/A	1.7.E-05	N/A
NIPSNAP3A	nipsnap homolog 3A (C. elegans)	-2.51	2.73	N/A	1.0.E-02	1.3.E-03	N/A
NKX2-8	NK2 homeobox 8	2.31	-3.27	N/A	1.2.E-03	3.7.E-07	N/A
NME7	non-metastatic cells 7, protein expressed in (nucleoside-diphosphate kinase)	-2.12	2.36	N/A	2.0.E-02	3.6.E-03	N/A
NMT1	N-myristoyltransferase 1	-2.11	2.10	N/A	1.5.E-03	4.2.E-04	N/A
NOC2L	nucleolar complex associated 2 homolog (S. cerevisiae)	N/A	-3.08	N/A	N/A	9.4.E-07	N/A
NOL8	nucleolar protein 8	2.22	N/A	N/A	1.2.E-03	N/A	N/A
NOLC1	nucleolar and coiled-body phosphoprotein 1	N/A	-2.54	N/A	N/A	1.7.E-04	N/A

NONO	non-POU domain containing, octamer-binding	-2.49	2.44	N/A	4.7.E-03	1.6.E-03	N/A
NOP14	NOP14 nucleolar protein homolog (yeast)	2.20	-2.82	N/A	1.2.E-03	1.8.E-05	N/A
NOP56	NOP56 ribonucleoprotein homolog (yeast)	N/A	-2.20	N/A	N/A	1.3.E-04	N/A
NOV	nephroblastoma overexpressed gene	-2.31	4.89	2.11	8.6.E-03	2.0.E-04	7.0.E-03
NOXA1	NADPH oxidase activator 1	N/A	-3.12	N/A	N/A	7.5.E-05	N/A
NQO1	NAD(P)H dehydrogenase, quinone 1	N/A	2.16	N/A	N/A	4.3.E-03	N/A
NQO2	NAD(P)H dehydrogenase, quinone 2	2.04	-2.01	N/A	1.2.E-03	4.6.E-03	N/A
NRG1	neuregulin 1	2.22	N/A	N/A	3.1.E-04	N/A	N/A
NRM	nurim (nuclear envelope membrane protein)	-6.17	4.32	N/A	7.0.E-04	1.7.E-04	N/A
NRTN	neurturin	-2.07	2.32	N/A	7.7.E-04	2.0.E-05	N/A
NSMF	NMDA receptor synaptonuclear signaling and neuronal migration factor	-2.41	N/A	N/A	3.7.E-02	N/A	N/A
NSUN6	NOP2/Sun domain family, member 6	2.11	-2.12	N/A	5.0.E-04	1.2.E-05	N/A
NTN4	netrin 4	2.17	N/A	N/A	3.3.E-03	N/A	N/A
NTRK1	neurotrophic tyrosine kinase, receptor, type 1	N/A	-2.56	N/A	N/A	2.4.E-07	N/A
NTRK2	neurotrophic tyrosine kinase, receptor, type 2	4.09	N/A	3.17	1.4.E-03	N/A	4.3.E-02
NUCKS1	nuclear casein kinase and cyclin-dependent kinase substrate 1	2.39	-2.84	N/A	7.4.E-04	2.2.E-05	N/A
NUDT22	nudix (nucleoside diphosphate linked moiety X)-type motif 22	-2.19	2.27	N/A	9.4.E-04	1.1.E-04	N/A
NUDT7	nudix (nucleoside diphosphate linked moiety X)-type motif 7	N/A	-3.04	N/A	N/A	7.1.E-05	N/A
NUMBL	numb homolog (Drosophila)-like	N/A	-2.33	-2.10	N/A	4.0.E-05	1.4.E-03
NUP153	nucleoporin 153kDa	-2.89	2.07	N/A	1.0.E-03	1.0.E-03	N/A
NXPE4	neurexophilin and PC-esterase domain family, member 4	N/A	-3.37	-2.38	N/A	5.4.E-06	1.2.E-05
OAZ1	ornithine decarboxylase antizyme 1	-2.44	2.63	N/A	2.4.E-03	5.4.E-04	N/A
OBFC1	oligonucleotide/oligosaccharide-binding fold containing 1	-2.72	3.42	N/A	3.3.E-04	1.5.E-06	N/A

OGDH	oxoglutarate (alpha-ketoglutarate) dehydrogenase (lipoamide)	-3.06	3.10	N/A	2.2.E-05	9.8.E-08	N/A
ORAI1	ORAI calcium release-activated calcium modulator 1	N/A	-2.37	N/A	N/A	1.9.E-06	N/A
OSBPL2	oxysterol binding protein-like 2	-2.13	N/A	N/A	1.4.E-03	N/A	N/A
OSBPL9	oxysterol binding protein-like 9	2.31	-2.82	N/A	1.8.E-04	4.6.E-06	N/A
OSGIN2	oxidative stress induced growth inhibitor family member 2	2.22	-2.70	N/A	1.1.E-03	2.6.E-05	N/A
OSTM1	osteopetrosis associated transmembrane protein 1	-2.40	3.06	N/A	8.8.E-04	1.1.E-06	N/A
OTUB2	OTU domain, ubiquitin aldehyde binding 2	-2.37	3.35	N/A	5.4.E-03	6.5.E-06	N/A
OTUD5	OTU domain containing 5	2.18	-2.03	N/A	3.3.E-03	1.8.E-04	N/A
OTUD7B	OTU domain containing 7B	2.57	-2.49	N/A	4.1.E-03	2.3.E-03	N/A
OXSR1	oxidative-stress responsive 1	2.07	N/A	N/A	1.6.E-03	N/A	N/A
P4HB	prolyl 4-hydroxylase, beta polypeptide	-2.25	2.67	N/A	3.6.E-03	5.1.E-03	N/A
PAAF1	proteasomal ATPase-associated factor 1	-2.25	2.12	N/A	3.4.E-03	2.0.E-03	N/A
PABPC1	poly(A) binding protein, cytoplasmic 1	2.18	-2.21	N/A	1.8.E-04	1.4.E-06	N/A
PABPC1P10	poly(A) binding protein, cytoplasmic 1 pseudogene 10	3.84	-4.53	N/A	2.9.E-05	2.2.E-08	N/A
PABPN1	poly(A) binding protein, nuclear 1	2.55	-2.26	N/A	6.6.E-04	7.8.E-05	N/A
PACSIN1	protein kinase C and casein kinase substrate in neurons 1	N/A	2.85	N/A	N/A	5.5.E-04	N/A
PACSIN2	protein kinase C and casein kinase substrate in neurons 2	-3.11	2.48	N/A	3.0.E-03	3.9.E-03	N/A
PADI1	peptidyl arginine deiminase, type I	N/A	2.49	N/A	N/A	1.8.E-02	N/A
PAFAH1B2	platelet-activating factor acetylhydrolase 1b, catalytic subunit 2 (30kDa)	-2.12	2.78	N/A	8.3.E-03	4.0.E-04	N/A
PAK1	p21 protein (Cdc42/Rac)-activated kinase 1	2.08	N/A	N/A	1.8.E-04	N/A	N/A
PAM	peptidylglycine alpha-amidating monooxygenase	N/A	-2.19	N/A	N/A	1.1.E-05	N/A
PAPOLG	poly(A) polymerase gamma	N/A	2.70	N/A	N/A	6.6.E-05	N/A
PARK2	parkinson protein 2, E3 ubiquitin protein ligase (parkin)	2.13	-2.97	N/A	2.0.E-03	6.9.E-06	N/A
PARM1	prostate androgen-regulated mucin-like protein 1	2.50	-2.02	N/A	1.4.E-03	3.5.E-03	N/A

PARP1	poly (ADP-ribose) polymerase 1	-2.30	N/A	N/A	3.5.E-03	N/A	N/A
PAWR	PRKC, apoptosis, WT1, regulator	N/A	-2.17	N/A	N/A	6.9.E-04	N/A
PBXIP1	pre-B-cell leukemia homeobox interacting protein 1	N/A	-2.68	-2.67	N/A	9.9.E-06	7.4.E-06
PCBP2	poly(rC) binding protein 2	-3.14	3.26	N/A	1.0.E-03	2.7.E-05	N/A
PCMT1	protein-L-isoaspartate (D-aspartate) O-methyltransferase	-2.05	2.49	N/A	2.6.E-03	9.9.E-05	N/A
PDCD5	programmed cell death 5	N/A	N/A	-2.01	N/A	N/A	3.6.E-05
PDCD6IP	programmed cell death 6 interacting protein	-2.18	2.20	N/A	1.1.E-04	6.6.E-07	N/A
PDE6A	phosphodiesterase 6A, cGMP-specific, rod, alpha	N/A	2.83	N/A	N/A	1.9.E-03	N/A
PDE9A	phosphodiesterase 9A	N/A	-2.15	N/A	N/A	1.4.E-04	N/A
PDHA1	pyruvate dehydrogenase (lipoamide) alpha 1	2.22	-2.59	N/A	1.2.E-03	1.9.E-04	N/A
PDIA4	protein disulfide isomerase family A, member 4	2.41	-3.03	N/A	3.4.E-03	5.9.E-05	N/A
PDIA6	protein disulfide isomerase family A, member 6	N/A	2.20	N/A	N/A	1.5.E-04	N/A
PDK3	pyruvate dehydrogenase kinase, isozyme 3	-3.28	4.80	N/A	2.8.E-04	6.4.E-06	N/A
PDLIM1	PDZ and LIM domain 1	-2.33	2.19	N/A	2.7.E-03	4.9.E-04	N/A
PDLIM2	PDZ and LIM domain 2 (mystique)	N/A	2.38	N/A	N/A	1.3.E-04	N/A
PDXP	pyridoxal (pyridoxine, vitamin B6) phosphatase	-2.23	N/A	N/A	7.5.E-04	N/A	N/A
PDZD3	PDZ domain containing 3	-2.11	2.42	N/A	4.7.E-03	2.4.E-04	N/A
PDZK1IP1	PDZK1 interacting protein 1	2.57	N/A	2.67	1.8.E-03	N/A	3.1.E-05
PEPD	peptidase D	-2.19	2.42	N/A	3.6.E-03	1.3.E-04	N/A
PEX1	peroxisomal biogenesis factor 1	N/A	2.15	N/A	N/A	4.4.E-04	N/A
PEX26	peroxisomal biogenesis factor 26	2.06	-2.99	N/A	1.8.E-03	1.7.E-05	N/A
PEX7	peroxisomal biogenesis factor 7	2.87	-4.65	N/A	9.3.E-04	5.0.E-06	N/A
PFAS	phosphoribosylformylglycinamidine synthase	N/A	N/A	2.10	N/A	N/A	4.7.E-05
PFDN1	prefoldin subunit 1	-2.03	2.18	N/A	1.3.E-02	6.8.E-03	N/A
PFDN5	prefoldin subunit 5	N/A	-2.73	N/A	N/A	1.2.E-04	N/A

PFKP	phosphofructokinase, platelet	2.05	-3.13	N/A	2.5.E-03	1.0.E-04	N/A
PGK1	phosphoglycerate kinase 1	-4.67	4.57	N/A	7.1.E-04	1.7.E-04	N/A
PGM1	phosphoglucomutase 1	N/A	2.17	N/A	N/A	5.4.E-05	N/A
PGM2	phosphoglucomutase 2	2.28	-2.08	N/A	1.4.E-03	5.9.E-04	N/A
PGM3	phosphoglucomutase 3	N/A	2.09	N/A	N/A	1.3.E-03	N/A
PGM5	phosphoglucomutase 5	N/A	-2.03	N/A	N/A	8.9.E-03	N/A
PGRMC1	progesterone receptor membrane component 1	-3.55	3.50	N/A	1.0.E-03	5.1.E-05	N/A
PHAX	phosphorylated adaptor for RNA export	-2.10	2.28	N/A	5.8.E-03	1.3.E-03	N/A
PHF1	PHD finger protein 1	N/A	-2.15	N/A	N/A	5.1.E-05	N/A
PHF20L1	PHD finger protein 20-like 1	2.30	-2.38	N/A	1.5.E-03	9.2.E-05	N/A
PHIP	pleckstrin homology domain interacting protein	2.75	-2.30	N/A	2.0.E-03	2.9.E-03	N/A
PHOX2A	paired-like homeobox 2a	2.00	-2.07	N/A	2.2.E-03	2.4.E-05	N/A
PHYHIP	phytanoyl-CoA 2-hydroxylase interacting protein	-2.28	3.92	N/A	1.9.E-02	2.2.E-04	N/A
PI3	peptidase inhibitor 3, skin-derived (SKALP)	N/A	4.85	N/A	N/A	4.0.E-02	N/A
PIGK	phosphatidylinositol glycan anchor biosynthesis, class K	N/A	2.07	N/A	N/A	5.8.E-04	N/A
PIGM	phosphatidylinositol glycan anchor biosynthesis, class M	N/A	-2.08	N/A	N/A	6.1.E-05	N/A
PIGP	phosphatidylinositol glycan anchor biosynthesis, class P	-2.18	3.07	N/A	2.5.E-03	2.5.E-05	N/A
PIGS	phosphatidylinositol glycan anchor biosynthesis, class S	-2.91	2.68	N/A	2.6.E-03	6.7.E-04	N/A
PIN1	peptidylprolyl cis/trans isomerase, NIMA-interacting 1	-2.83	2.29	N/A	5.0.E-04	2.0.E-04	N/A
PIP	prolactin-induced protein	-3.21	3.85	N/A	5.7.E-03	1.9.E-04	N/A
PISD	phosphatidylserine decarboxylase	-2.01	2.57	N/A	1.6.E-03	9.3.E-05	N/A
PITHD1	PITH (C-terminal proteasome-interacting domain of thioredoxin-like) domain containing 1	-3.11	2.15	N/A	3.3.E-03	8.0.E-03	N/A
PITPNB	phosphatidylinositol transfer protein, beta	-2.24	N/A	N/A	1.0.E-03	N/A	N/A
PITX1	paired-like homeodomain 1	-2.73	3.82	N/A	2.7.E-04	2.4.E-06	N/A
PITX3	paired-like homeodomain 3	N/A	2.15	2.70	N/A	1.5.E-05	3.4.E-05

PJA2	praja ring finger 2	2.89	-3.28	N/A	2.7.E-04	3.5.E-06	N/A
PKIB	protein kinase (cAMP-dependent, catalytic) inhibitor beta	N/A	2.23	N/A	N/A	2.3.E-06	N/A
PLAGL1	pleiomorphic adenoma gene-like 1	N/A	2.05	N/A	N/A	1.3.E-02	N/A
PLD3	phospholipase D family, member 3	-2.76	3.17	N/A	1.1.E-04	1.2.E-06	N/A
PLD4	phospholipase D family, member 4	N/A	-2.02	N/A	N/A	6.7.E-05	N/A
PLEKHA1	pleckstrin homology domain containing, family A (phosphoinositide binding specific) member 1	-2.50	2.49	N/A	1.1.E-04	1.4.E-07	N/A
PLGRKT	plasminogen receptor, C-terminal lysine transmembrane protein	N/A	2.09	N/A	N/A	2.7.E-04	N/A
PLIN2	perilipin 2	-2.05	N/A	N/A	1.8.E-03	N/A	N/A
PLIN3	perilipin 3	N/A	-2.28	N/A	N/A	6.7.E-05	N/A
PLS3	plastin 3	-6.87	8.87	N/A	7.2.E-04	1.5.E-05	N/A
PLXND1	plexin D1	2.37	-3.41	N/A	5.5.E-04	2.8.E-05	N/A
PML	promyelocytic leukemia	N/A	-2.26	N/A	N/A	2.2.E-05	N/A
PNO1	partner of NOB1 homolog (S. cerevisiae)	N/A	2.16	N/A	N/A	2.1.E-03	N/A
PNP	purine nucleoside phosphorylase	-2.02	2.36	N/A	3.5.E-03	1.5.E-04	N/A
PNRC2	proline-rich nuclear receptor coactivator 2	3.14	-4.42	N/A	5.4.E-04	4.6.E-06	N/A
POC1A	POC1 centriolar protein homolog A (Chlamydomonas)	-2.23	2.08	N/A	7.0.E-04	1.8.E-05	N/A
POLH	polymerase (DNA directed), eta	2.13	-2.92	N/A	1.3.E-03	1.8.E-05	N/A
POLR2B	polymerase (RNA) II (DNA directed) polypeptide B, 140kDa	N/A	2.42	N/A	N/A	2.2.E-06	N/A
POLR2D	polymerase (RNA) II (DNA directed) polypeptide D	N/A	2.18	N/A	N/A	1.0.E-03	N/A
POLR2K	polymerase (RNA) II (DNA directed) polypeptide K, 7.0kDa	N/A	-2.87	N/A	N/A	9.5.E-06	N/A
POLR3C	polymerase (RNA) III (DNA directed) polypeptide C (62kD)	N/A	-2.06	N/A	N/A	1.3.E-03	N/A
POLR3K	polymerase (RNA) III (DNA directed) polypeptide K, 12.3 kDa	N/A	2.34	N/A	N/A	1.0.E-04	N/A
PON2	paraoxonase 2	-2.17	3.11	N/A	3.2.E-03	4.3.E-05	N/A

POP7	processing of precursor 7, ribonuclease P/MRP subunit (S. cerevisiae)	N/A	2.16	N/A	N/A	6.0.E-04	N/A
POSTN	periostin, osteoblast specific factor	N/A	-2.16	N/A	N/A	3.6.E-04	N/A
PPAN	peter pan homolog (Drosophila)	N/A	-2.03	N/A	N/A	4.6.E-04	N/A
PPAP2C	phosphatidic acid phosphatase type 2C	-3.49	3.47	N/A	5.3.E-04	6.8.E-05	N/A
PPCDC	phosphopantothenoylcysteine decarboxylase	-2.03	2.08	N/A	2.2.E-04	9.6.E-07	N/A
PPCS	phosphopantothenoylcysteine synthetase	N/A	2.04	N/A	N/A	3.1.E-04	N/A
PPIB	peptidylprolyl isomerase B (cyclophilin B)	-2.64	2.60	N/A	5.2.E-04	1.0.E-04	N/A
PPIF	peptidylprolyl isomerase F	-2.86	2.24	N/A	2.1.E-03	1.7.E-03	N/A
PPL	periplakin	-2.74	2.47	N/A	3.0.E-04	2.0.E-05	N/A
PPM1H	protein phosphatase, Mg2+/Mn2+ dependent, 1H	N/A	2.30	N/A	N/A	6.1.E-04	N/A
PPP1CA	protein phosphatase 1, catalytic subunit, alpha isozyme	-5.90	5.89	N/A	2.0.E-04	4.3.E-06	N/A
PPP1CB	protein phosphatase 1, catalytic subunit, beta isozyme	-2.61	3.24	N/A	1.8.E-03	1.3.E-04	N/A
PPP1CC	protein phosphatase 1, catalytic subunit, gamma isozyme	N/A	-2.11	N/A	N/A	2.1.E-03	N/A
PPP1R11	protein phosphatase 1, regulatory (inhibitor) subunit 11	-2.13	2.91	N/A	2.2.E-04	3.7.E-07	N/A
PPP1R12A	protein phosphatase 1, regulatory subunit 12A	2.52	-3.23	N/A	2.8.E-04	1.6.E-06	N/A
PPP1R37	protein phosphatase 1, regulatory subunit 37	N/A	-2.46	N/A	N/A	1.3.E-04	N/A
PPP1R8	protein phosphatase 1, regulatory subunit 8	-2.54	2.53	N/A	5.5.E-03	1.1.E-03	N/A
PPP2CB	protein phosphatase 2, catalytic subunit, beta isozyme	N/A	2.47	N/A	N/A	2.8.E-05	N/A
PPP2R2A	protein phosphatase 2, regulatory subunit B, alpha	-2.96	3.03	N/A	5.0.E-04	1.6.E-06	N/A
PPP2R4	protein phosphatase 2A activator, regulatory subunit 4	-2.48	2.48	N/A	4.3.E-04	8.3.E-05	N/A
PPP2R5D	protein phosphatase 2, regulatory subunit B', delta	N/A	-2.16	N/A	N/A	7.1.E-06	N/A
PPP4C	protein phosphatase 4, catalytic subunit	N/A	-2.54	-2.03	N/A	1.4.E-05	3.7.E-06
PPP4R2	protein phosphatase 4, regulatory subunit 2	2.12	-3.14	N/A	7.5.E-04	1.8.E-05	N/A
PRC1	protein regulator of cytokinesis 1	-2.57	N/A	N/A	1.3.E-02	N/A	N/A
PREP	prolyl endopeptidase	-2.37	3.31	N/A	3.0.E-04	2.7.E-07	N/A

PRKAR1B	protein kinase, cAMP-dependent, regulatory, type I, beta	2.32	-2.57	N/A	1.3.E-04	1.1.E-05	N/A
PRKAR2A	protein kinase, cAMP-dependent, regulatory, type II, alpha	N/A	-2.54	N/A	N/A	2.7.E-06	N/A
PRKCDBP	protein kinase C, delta binding protein	2.02	-2.03	N/A	1.3.E-03	5.6.E-04	N/A
PRKCSH	protein kinase C substrate 80K-H	-2.64	2.04	N/A	2.2.E-04	5.3.E-05	N/A
PRKG1	protein kinase, cGMP-dependent, type I	2.76	N/A	N/A	1.3.E-03	N/A	N/A
PRKX	protein kinase, X-linked	-2.14	2.49	N/A	4.3.E-04	2.3.E-06	N/A
PRND	prion protein 2 (dublet)	-3.66	N/A	-2.18	1.3.E-02	N/A	1.9.E-02
PRNP	prion protein	2.32	-4.69	-2.02	9.1.E-03	1.6.E-04	4.5.E-05
PRODH	proline dehydrogenase (oxidase) 1	2.76	-2.71	N/A	4.8.E-04	1.4.E-03	N/A
PRPF38A	PRP38 pre-mRNA processing factor 38 (yeast) domain containing A	N/A	-2.12	N/A	N/A	4.3.E-07	N/A
PRPF8	PRP8 pre-mRNA processing factor 8 homolog (S. cerevisiae)	-3.74	3.85	N/A	3.3.E-04	6.0.E-06	N/A
PRR14	proline rich 14	2.02	N/A	N/A	6.8.E-04	N/A	N/A
PRR3	proline rich 3	2.05	-2.82	N/A	2.0.E-03	1.1.E-06	N/A
PRSS23	protease, serine, 23	-2.65	2.12	N/A	3.7.E-03	3.7.E-03	N/A
PRSS27	protease, serine 27	N/A	2.10	N/A	N/A	1.3.E-02	N/A
PRSS53	protease, serine, 53	-2.94	N/A	N/A	4.3.E-02	N/A	N/A
PSEN1	presenilin 1	-2.05	2.02	N/A	1.0.E-03	4.4.E-05	N/A
PSIP1	PC4 and SFRS1 interacting protein 1	2.31	-2.74	N/A	2.0.E-03	4.8.E-04	N/A
PSMB8	proteasome (prosome, macropain) subunit, beta type, 8 (large multifunctional peptidase 7)	N/A	-2.58	N/A	N/A	3.8.E-05	N/A
PSMC3	proteasome (prosome, macropain) 26S subunit, ATPase, 3	-2.85	2.75	N/A	3.3.E-04	1.1.E-04	N/A
PSMD10	proteasome (prosome, macropain) 26S subunit, non-ATPase, 10	N/A	2.37	N/A	N/A	4.3.E-05	N/A
PSMG3	proteasome (prosome, macropain) assembly chaperone 3	-2.05	N/A	N/A	3.3.E-03	N/A	N/A
PTGES2	prostaglandin E synthase 2	-2.41	N/A	N/A	4.1.E-03	N/A	N/A

PTGES3	prostaglandin E synthase 3 (cytosolic)	N/A	-3.16	N/A	N/A	7.7.E-07	N/A
PTGFRN	prostaglandin F2 receptor inhibitor	N/A	-2.02	N/A	N/A	5.7.E-05	N/A
PTGR2	prostaglandin reductase 2	N/A	-2.23	N/A	N/A	1.2.E-04	N/A
PTPN11	protein tyrosine phosphatase, non-receptor type 11	3.26	-3.68	N/A	5.7.E-04	9.1.E-06	N/A
PTPN12	protein tyrosine phosphatase, non-receptor type 12	2.89	-5.43	N/A	3.1.E-04	1.4.E-05	N/A
PTPN6	protein tyrosine phosphatase, non-receptor type 6	-3.11	3.62	N/A	8.8.E-04	1.1.E-06	N/A
PVRL2	poliovirus receptor-related 2 (herpesvirus entry mediator B)	-2.71	2.98	N/A	1.1.E-04	3.3.E-09	N/A
PXK	PX domain containing serine/threonine kinase	N/A	2.11	N/A	N/A	1.7.E-03	N/A
QDPR	quinoid dihydropteridine reductase	-2.75	2.48	N/A	2.7.E-04	1.1.E-05	N/A
RAB11A	RAB11A, member RAS oncogene family	N/A	2.21	N/A	N/A	1.8.E-05	N/A
RAB14	RAB14, member RAS oncogene family	-2.50	2.26	N/A	2.2.E-04	2.6.E-06	N/A
RAB18	RAB18, member RAS oncogene family	-4.39	4.90	N/A	5.0.E-04	7.2.E-06	N/A
RAB19	RAB19, member RAS oncogene family	-3.04	7.11	2.34	1.0.E-02	2.1.E-06	8.3.E-03
RAB28	RAB28, member RAS oncogene family	-2.14	N/A	N/A	1.4.E-03	N/A	N/A
RAB7A	RAB7A, member RAS oncogene family	-2.52	2.64	N/A	1.2.E-03	1.6.E-04	N/A
RAB8A	RAB8A, member RAS oncogene family	-2.21	2.41	N/A	2.1.E-03	1.2.E-04	N/A
RAB9A	RAB9A, member RAS oncogene family	-3.35	2.85	N/A	2.1.E-04	5.7.E-06	N/A
RABGAP1	RAB GTPase activating protein 1	2.65	-2.90	N/A	4.8.E-04	5.1.E-05	N/A
RAD23B	RAD23 homolog B (S. cerevisiae)	2.41	-2.84	N/A	7.4.E-04	5.8.E-05	N/A
RAMP1	receptor (G protein-coupled) activity modifying protein 1	N/A	-2.17	N/A	N/A	1.3.E-03	N/A
RAN	RAN, member RAS oncogene family	-2.01	2.17	N/A	1.5.E-03	4.2.E-04	N/A
RASA1	RAS p21 protein activator (GTPase activating protein) 1	-2.18	3.11	N/A	4.4.E-03	1.0.E-04	N/A
RASSF1	Ras association (RalGDS/AF-6) domain family member 1	N/A	-2.44	N/A	N/A	1.7.E-05	N/A
RBBP7	retinoblastoma binding protein 7	-2.38	2.56	N/A	4.2.E-04	5.0.E-06	N/A
RBBP8	retinoblastoma binding protein 8	3.38	-4.51	N/A	2.1.E-04	6.3.E-07	N/A

RBM17	RNA binding motif protein 17	2.50	-2.27	N/A	1.3.E-03	3.3.E-04	N/A
RBM18	RNA binding motif protein 18	N/A	2.29	N/A	N/A	3.0.E-05	N/A
RBM25	RNA binding motif protein 25	2.35	-2.11	N/A	2.0.E-03	6.9.E-04	N/A
RCBTB2	regulator of chromosome condensation (RCC1) and BTB (POZ) domain containing protein 2	2.50	-2.75	N/A	1.2.E-03	1.1.E-04	N/A
RCN3	reticulocalbin 3, EF-hand calcium binding domain	2.16	-2.54	N/A	7.9.E-04	8.0.E-05	N/A
RDX	radixin	2.01	-2.12	N/A	2.1.E-04	1.1.E-05	N/A
REEP4	receptor accessory protein 4	-2.21	N/A	N/A	1.1.E-03	N/A	N/A
RER1	RER1 retention in endoplasmic reticulum 1 homolog (S. cerevisiae)	N/A	2.10	N/A	N/A	1.8.E-04	N/A
RFC3	replication factor C (activator 1) 3, 38kDa	-3.04	3.26	N/A	2.9.E-03	2.3.E-04	N/A
RFX7	regulatory factor X, 7	3.72	-4.35	N/A	1.6.E-04	1.0.E-06	N/A
RGP1	RGP1 retrograde golgi transport homolog (S. cerevisiae)	2.17	N/A	N/A	4.9.E-04	N/A	N/A
RGS2	regulator of G-protein signaling 2, 24kDa	N/A	2.07	N/A	N/A	4.5.E-03	N/A
RGS3	regulator of G-protein signaling 3	N/A	-2.46	N/A	N/A	5.4.E-05	N/A
RHBDL1	rhomboid, veinlet-like 1 (Drosophila)	N/A	N/A	2.00	N/A	N/A	3.2.E-04
RHEB	Ras homolog enriched in brain	-2.27	N/A	N/A	1.8.E-04	N/A	N/A
RHOA	ras homolog gene family, member A	-2.90	3.42	N/A	9.5.E-04	3.1.E-05	N/A
RHOU	ras homolog gene family, member U	2.38	-44.86	-18.86	1.2.E-03	1.1.E-09	3.8.E-07
RMND1	required for meiotic nuclear division 1 homolog (S. cerevisiae)	N/A	3.00	2.55	N/A	7.7.E-04	6.2.E-04
RNF103	ring finger protein 103	N/A	2.60	N/A	N/A	7.3.E-05	N/A
RNF121	ring finger protein 121	-2.86	3.28	N/A	2.8.E-04	3.3.E-06	N/A
RNF122	ring finger protein 122	N/A	-4.30	-4.33	N/A	9.1.E-05	3.4.E-04
RNF139	ring finger protein 139	-2.38	2.29	N/A	1.6.E-03	4.0.E-04	N/A
RNF14	ring finger protein 14	-2.03	2.02	N/A	9.2.E-03	4.4.E-03	N/A

RNF167	ring finger protein 167	-2.16	2.01	N/A	2.0.E-03	9.4.E-04	N/A
RNF170	ring finger protein 170	-2.32	3.16	N/A	3.6.E-03	1.2.E-04	N/A
RNF25	ring finger protein 25	N/A	-2.37	N/A	N/A	5.3.E-07	N/A
RNGTT	RNA guanylyltransferase and 5'-phosphatase	2.04	-2.20	N/A	6.8.E-04	4.6.E-05	N/A
RNPC3	RNA-binding region (RNP1, RRM) containing 3	2.39	-2.15	N/A	2.3.E-03	3.4.E-03	N/A
RNPEPL1	arginyl aminopeptidase (aminopeptidase B)-like 1	N/A	2.02	N/A	N/A	2.7.E-06	N/A
ROCK1	Rho-associated, coiled-coil containing protein kinase 1	2.00	N/A	N/A	2.3.E-03	N/A	N/A
ROM1	retinal outer segment membrane protein 1	2.01	-2.25	N/A	2.1.E-04	3.6.E-05	N/A
RPL12	ribosomal protein L12	N/A	-2.63	N/A	N/A	2.9.E-07	N/A
RPL13A	ribosomal protein L13a	-2.15	2.32	N/A	7.6.E-03	2.3.E-03	N/A
RPL15	ribosomal protein L15	-3.71	3.65	N/A	4.8.E-04	3.4.E-05	N/A
RPL17	ribosomal protein L17	N/A	-2.20	N/A	N/A	2.6.E-03	N/A
RPL21	ribosomal protein L21	N/A	-2.27	N/A	N/A	1.2.E-04	N/A
RPL28	ribosomal protein L28	N/A	-2.80	N/A	N/A	2.1.E-05	N/A
RPL29	ribosomal protein L29	2.00	N/A	N/A	1.9.E-03	N/A	N/A
RPL3	ribosomal protein L3	3.03	-6.66	-2.20	7.5.E-04	7.5.E-07	3.1.E-05
RPL34	ribosomal protein L34	N/A	N/A	-2.73	N/A	N/A	9.2.E-04
RPL37	ribosomal protein L37	2.99	-5.84	N/A	5.5.E-04	1.6.E-07	N/A
RPL6	ribosomal protein L6	2.02	N/A	N/A	2.0.E-03	N/A	N/A
RPL7L1	ribosomal protein L7-like 1	-3.98	4.14	N/A	6.7.E-04	1.3.E-04	N/A
RPLP0	ribosomal protein, large, P0	2.24	-3.25	N/A	1.1.E-04	3.0.E-07	N/A
RPLP2	ribosomal protein, large, P2	N/A	-3.15	-2.15	N/A	1.5.E-06	1.3.E-04
RPS12	ribosomal protein S12	2.42	-3.39	N/A	4.8.E-04	2.5.E-06	N/A
RPS14	ribosomal protein S14	-2.13	N/A	N/A	1.1.E-03	N/A	N/A
RPS19	ribosomal protein S19	N/A	-2.03	N/A	N/A	1.4.E-05	N/A

RPS2	ribosomal protein S2	-2.42	2.42	N/A	1.2.E-03	2.4.E-04	N/A
RPS24	ribosomal protein S24	-2.23	2.21	N/A	2.7.E-03	1.4.E-03	N/A
RPSA	ribosomal protein SA	-4.04	3.25	N/A	1.9.E-03	1.2.E-03	N/A
RRAD	Ras-related associated with diabetes	2.19	-2.35	N/A	1.4.E-02	3.0.E-03	N/A
RRAGA	Ras-related GTP binding A	N/A	2.31	N/A	N/A	2.5.E-04	N/A
RSRC1	arginine/serine-rich coiled-coil 1	-2.21	2.47	N/A	2.4.E-03	1.5.E-04	N/A
RSRC2	arginine/serine-rich coiled-coil 2	2.32	-2.42	N/A	3.0.E-03	4.7.E-04	N/A
RTEL1	regulator of telomere elongation helicase 1	N/A	-2.30	N/A	N/A	1.4.E-04	N/A
RTF1	Rtf1, Paf1/RNA polymerase II complex component, homolog (S. cerevisiae)	N/A	-2.25	N/A	N/A	3.8.E-03	N/A
RTN4	reticulon 4	-2.73	3.07	N/A	1.3.E-03	9.7.E-05	N/A
S100A14	S100 calcium binding protein A14	N/A	-2.61	N/A	N/A	1.3.E-05	N/A
S1PR2	sphingosine-1-phosphate receptor 2	N/A	-2.73	-2.75	N/A	4.2.E-05	2.0.E-05
SAFB	scaffold attachment factor B	N/A	-2.32	N/A	N/A	2.7.E-04	N/A
SAMD12	sterile alpha motif domain containing 12	-2.07	2.78	N/A	1.7.E-02	5.1.E-04	N/A
SAP18	Sin3A-associated protein, 18kDa	N/A	2.20	N/A	N/A	1.5.E-04	N/A
SARS	seryl-tRNA synthetase	-2.54	2.57	N/A	1.4.E-03	3.5.E-04	N/A
SART3	squamous cell carcinoma antigen recognized by T cells 3	-2.98	2.06	N/A	1.5.E-03	4.4.E-03	N/A
SC5DL	sterol-C5-desaturase (ERG3 delta-5-desaturase homolog, S. cerevisiae)-like	-2.87	4.33	N/A	8.2.E-03	7.0.E-04	N/A
SCAMP2	secretory carrier membrane protein 2	-3.22	2.67	N/A	7.6.E-05	8.2.E-06	N/A
SCAMP3	secretory carrier membrane protein 3	-4.53	4.99	N/A	2.0.E-03	2.5.E-04	N/A
SCARA3	scavenger receptor class A, member 3	2.10	N/A	N/A	1.2.E-03	N/A	N/A
SCGB1A1	secretoglobin, family 1A, member 1 (uteroglobin)	N/A	N/A	2.51	N/A	N/A	1.3.E-04
SCNN1A	sodium channel, non-voltage-gated 1 alpha subunit	-2.40	2.82	N/A	5.9.E-03	1.0.E-04	N/A
SCP2	sterol carrier protein 2	-2.01	N/A	N/A	1.0.E-02	N/A	N/A

SDCBP	syndecan binding protein (syntenin)	-2.08	2.54	N/A	5.5.E-03	7.0.E-05	N/A
SDCBP2	syndecan binding protein (syntenin) 2	-3.35	5.44	N/A	8.1.E-03	4.8.E-05	N/A
SDF2	stromal cell-derived factor 2	-2.64	2.79	N/A	8.9.E-04	1.1.E-04	N/A
SDHA	succinate dehydrogenase complex, subunit A, flavoprotein (Fp)	-7.32	7.18	N/A	2.7.E-06	2.1.E-10	N/A
SDHB	succinate dehydrogenase complex, subunit B, iron sulfur (Ip)	-3.18	3.57	N/A	4.6.E-04	9.9.E-06	N/A
SDHC	succinate dehydrogenase complex, subunit C, integral membrane protein, 15kDa	-3.42	2.78	N/A	3.9.E-05	1.3.E-07	N/A
SDR16C5	short chain dehydrogenase/reductase family 16C, member 5	N/A	2.46	N/A	N/A	4.0.E-05	N/A
SEC24A	SEC24 family, member A (S. cerevisiae)	-2.07	N/A	N/A	2.4.E-03	N/A	N/A
SEC62	SEC62 homolog (S. cerevisiae)	-2.39	3.04	N/A	1.1.E-03	6.0.E-06	N/A
SEC63	SEC63 homolog (S. cerevisiae)	N/A	2.14	N/A	N/A	1.6.E-03	N/A
SECISBP2	SECIS binding protein 2	-3.11	2.20	N/A	1.5.E-02	4.6.E-02	N/A
SEL1L3	sel-1 suppressor of lin-12-like 3 (C. elegans)	-2.16	N/A	N/A	2.9.E-03	N/A	N/A
SELENBP1	selenium binding protein 1	-5.90	5.34	N/A	1.1.E-04	1.6.E-05	N/A
SENP3	SUMO1/sentrin/SMT3 specific peptidase 3	N/A	-2.14	N/A	N/A	5.8.E-08	N/A
SEPW1	selenoprotein W, 1	-2.63	N/A	N/A	4.1.E-03	N/A	N/A
SERINC2	serine incorporator 2	N/A	2.19	N/A	N/A	7.7.E-07	N/A
SERPINA12	serpin peptidase inhibitor, clade A (alpha-1 antiproteinase, antitrypsin), member 12	2.04	N/A	2.29	2.0.E-03	N/A	7.0.E-05
SERPINB10	serpin peptidase inhibitor, clade B (ovalbumin), member 10	N/A	2.10	N/A	N/A	3.0.E-05	N/A
SERPINB2	serpin peptidase inhibitor, clade B (ovalbumin), member 2	-2.86	2.78	N/A	2.3.E-04	7.9.E-06	N/A
SERPINB4	serpin peptidase inhibitor, clade B (ovalbumin), member 4	-3.53	2.91	N/A	4.2.E-03	1.2.E-03	N/A
SERPINB5	serpin peptidase inhibitor, clade B (ovalbumin), member 5	2.02	N/A	N/A	5.0.E-03	N/A	N/A
SERPINB6	serpin peptidase inhibitor, clade B (ovalbumin), member 6	-2.08	2.65	N/A	5.9.E-04	6.5.E-06	N/A
SERPINE1	serpin peptidase inhibitor, clade E (nexin, plasminogen activator inhibitor type 1), member 1	N/A	-2.46	N/A	N/A	4.7.E-02	N/A

SETD5	SET domain containing 5	N/A	-2.12	N/A	N/A	5.7.E-04	N/A
SF3B1	splicing factor 3b, subunit 1, 155kDa	2.29	-2.68	N/A	1.4.E-03	2.9.E-04	N/A
SFN	stratifin	-2.05	2.28	N/A	1.4.E-03	1.1.E-03	N/A
SFPQ	splicing factor proline/glutamine-rich	3.56	-5.41	N/A	4.1.E-04	9.5.E-06	N/A
SFRS18	splicing factor, arginine/serine-rich 18	N/A	-3.77	-2.01	N/A	7.0.E-05	2.1.E-03
SFT2D1	SFT2 domain containing 1	-2.14	2.47	N/A	1.0.E-03	9.5.E-05	N/A
SGOL2	shugoshin-like 2 (S. pombe)	2.93	-3.12	N/A	4.5.E-04	6.7.E-05	N/A
SGPL1	sphingosine-1-phosphate lyase 1	-2.93	2.11	N/A	3.8.E-03	5.1.E-03	N/A
SH3D19	SH3 domain containing 19	2.38	N/A	N/A	1.1.E-03	N/A	N/A
SH3GL1	SH3-domain GRB2-like 1	-2.92	2.15	N/A	5.1.E-04	1.2.E-04	N/A
SHOC2	soc-2 suppressor of clear homolog (C. elegans)	N/A	-2.24	N/A	N/A	4.8.E-07	N/A
SHPRH	SNF2 histone linker PHD RING helicase	2.76	-2.67	N/A	6.1.E-04	1.3.E-04	N/A
SIGLEC10	sialic acid binding Ig-like lectin 10	N/A	2.09	N/A	N/A	1.5.E-04	N/A
SKP1	S-phase kinase-associated protein 1	N/A	2.05	N/A	N/A	9.1.E-06	N/A
SLA2	Src-like-adaptor 2	-4.11	10.89	2.65	2.8.E-04	5.0.E-12	8.0.E-04
SLC10A5	solute carrier family 10, member 5	2.72	N/A	N/A	2.0.E-04	N/A	N/A
SLC22A18	solute carrier family 22, member 18	-2.49	2.20	N/A	2.0.E-04	8.0.E-06	N/A
SLC22A5	solute carrier family 22 (organic cation/carnitine transporter), member 5	N/A	-3.68	N/A	N/A	1.9.E-08	N/A
SLC22A8	solute carrier family 22 (organic anion transporter), member 8	-3.50	6.75	N/A	2.7.E-03	5.3.E-07	N/A
SLC23A3	solute carrier family 23 (nucleobase transporters), member 3	N/A	-2.32	N/A	N/A	1.5.E-04	N/A
SLC25A11	solute carrier family 25 (mitochondrial carrier; oxoglutarate carrier), member 11	-2.48	2.38	N/A	6.0.E-04	3.3.E-05	N/A
SLC25A17	solute carrier family 25 (mitochondrial carrier; peroxisomal membrane protein, 34kDa), member 17	N/A	2.10	N/A	N/A	1.2.E-04	N/A

SLC25A5	solute carrier family 25 (mitochondrial carrier; adenine nucleotide translocator), member 5	-3.30	2.99	N/A	4.8.E-04	2.2.E-04	N/A
SLC25A6	solute carrier family 25 (mitochondrial carrier; adenine nucleotide translocator), member 6	-5.59	4.80	N/A	5.1.E-04	5.4.E-05	N/A
SLC26A3	solute carrier family 26, member 3	-6.38	10.80	N/A	3.4.E-03	6.4.E-05	N/A
SLC28A3	solute carrier family 28 (sodium-coupled nucleoside transporter), member 3	-6.71	10.20	N/A	1.3.E-03	9.9.E-06	N/A
SLC29A4	solute carrier family 29 (nucleoside transporters), member 4	N/A	2.45	2.32	N/A	7.6.E-07	1.6.E-03
SLC35A5	solute carrier family 35, member A5	-2.73	2.52	N/A	8.0.E-03	3.9.E-03	N/A
SLC35C1	solute carrier family 35, member C1	N/A	-2.09	N/A	N/A	2.4.E-06	N/A
SLC35F5	solute carrier family 35, member F5	-2.63	3.83	N/A	6.8.E-04	5.5.E-06	N/A
SLC39A11	solute carrier family 39 (metal ion transporter), member 11	N/A	-2.17	-2.92	N/A	8.0.E-06	9.9.E-05
SLC39A7	solute carrier family 39 (zinc transporter), member 7	N/A	-2.46	N/A	N/A	2.0.E-08	N/A
SLC5A12	solute carrier family 5 (sodium/glucose cotransporter), member 12	2.24	-2.54	N/A	1.8.E-03	1.2.E-04	N/A
SLC6A8	solute carrier family 6 (neurotransmitter transporter, creatine), member 8	-2.01	N/A	N/A	1.3.E-03	N/A	N/A
SLC7A6OS	solute carrier family 7, member 6 opposite strand	N/A	-2.05	N/A	N/A	3.2.E-03	N/A
SLC9A6	solute carrier family 9 (sodium/hydrogen exchanger), member 6	-4.08	4.08	N/A	7.9.E-04	1.6.E-05	N/A
SLCO2A1	solute carrier organic anion transporter family, member 2A1	N/A	N/A	-2.08	N/A	N/A	4.9.E-04
SLFNL1	schlafen-like 1	N/A	-2.85	-3.91	N/A	7.9.E-05	5.2.E-04
SLK	STE20-like kinase	N/A	2.08	N/A	N/A	3.5.E-05	N/A
SLU7	SLU7 splicing factor homolog (S. cerevisiae)	2.32	-2.02	N/A	7.3.E-03	6.7.E-03	N/A
SLURP1	secreted LY6/PLAUR domain containing 1	N/A	2.83	N/A	N/A	2.7.E-04	N/A
SMARCA4	SWI/SNF related, matrix associated, actin dependent regulator of chromatin, subfamily a, member 4	2.12	N/A	N/A	2.3.E-03	N/A	N/A

SMNDC1	survival motor neuron domain containing 1	2.20	-3.63	N/A	1.1.E-03	1.1.E-05	N/A
SMPD2	sphingomyelin phosphodiesterase 2, neutral membrane (neutral sphingomyelinase)	-2.52	5.24	2.08	8.8.E-03	1.8.E-06	1.4.E-02
SMPDL3B	sphingomyelin phosphodiesterase, acid-like 3B	-2.22	3.73	N/A	1.2.E-02	4.9.E-05	N/A
SMS	spermine synthase	-2.27	2.63	N/A	1.4.E-03	1.4.E-04	N/A
SNRNP40	small nuclear ribonucleoprotein 40kDa (U5)	N/A	-2.30	N/A	N/A	5.7.E-07	N/A
SNRNP48	small nuclear ribonucleoprotein 48kDa (U11/U12)	N/A	-2.12	N/A	N/A	1.7.E-06	N/A
SNRNP70	small nuclear ribonucleoprotein 70kDa (U1)	N/A	-2.37	N/A	N/A	6.1.E-06	N/A
SNRPF	small nuclear ribonucleoprotein polypeptide F	N/A	-4.12	-2.39	N/A	6.0.E-07	4.9.E-06
SNUPN	snurportin 1	3.42	-3.37	N/A	5.9.E-04	3.0.E-05	N/A
SNX2	sorting nexin 2	-2.03	2.34	N/A	1.3.E-02	1.4.E-03	N/A
SNX21	sorting nexin family member 21	2.15	-2.42	N/A	1.1.E-03	1.1.E-05	N/A
SNX3	sorting nexin 3	-2.61	3.34	N/A	1.6.E-03	2.7.E-05	N/A
SNX7	sorting nexin 7	-2.01	N/A	N/A	9.7.E-03	N/A	N/A
SOD2	superoxide dismutase 2, mitochondrial	-2.29	2.32	N/A	2.2.E-04	5.2.E-05	N/A
SOWAHD	sosondowah ankyrin repeat domain family member D	2.15	N/A	N/A	3.3.E-03	N/A	N/A
SOX18	SRY (sex determining region Y)-box 18	N/A	-2.31	N/A	N/A	6.6.E-05	N/A
SOX2	SRY (sex determining region Y)-box 2	N/A	-3.40	N/A	N/A	1.9.E-06	N/A
SOX4	SRY (sex determining region Y)-box 4	2.75	-2.52	N/A	7.4.E-04	8.6.E-04	N/A
SP140L	SP140 nuclear body protein-like	N/A	-2.10	N/A	N/A	4.4.E-05	N/A
SPAG9	sperm associated antigen 9	2.01	-2.20	N/A	8.0.E-04	9.3.E-05	N/A
SPC24	SPC24, NDC80 kinetochore complex component, homolog (S. cerevisiae)	2.28	-3.83	N/A	1.0.E-03	5.3.E-05	N/A
SPCS1	signal peptidase complex subunit 1 homolog (S. cerevisiae)	-2.24	3.37	N/A	2.0.E-03	3.1.E-05	N/A
SPCS3	signal peptidase complex subunit 3 homolog (S. cerevisiae)	-2.51	3.12	N/A	1.1.E-03	2.3.E-05	N/A
SPEG	SPEG complex locus	2.25	-3.20	N/A	4.8.E-04	1.8.E-05	N/A

SPG21	spastic paraplegia 21 (autosomal recessive, Mast syndrome)	-3.44	3.87	N/A	2.3.E-03	2.9.E-04	N/A
SPINK5	serine peptidase inhibitor, Kazal type 5	-3.40	5.64	N/A	2.2.E-03	3.3.E-06	N/A
SPNS1	spinster homolog 1 (Drosophila)	-2.23	N/A	N/A	1.2.E-03	N/A	N/A
SPON2	spondin 2, extracellular matrix protein	2.33	N/A	N/A	1.1.E-02	N/A	N/A
SPPL3	signal peptide peptidase like 3	-3.75	3.49	N/A	1.3.E-03	5.0.E-04	N/A
SPTLC1	serine palmitoyltransferase, long chain base subunit 1	-2.97	3.75	N/A	2.7.E-04	6.1.E-07	N/A
SPTSSB	serine palmitoyltransferase, small subunit B	-3.84	3.68	N/A	2.3.E-03	4.2.E-04	N/A
SQSTM1	sequestosome 1	-5.23	3.80	N/A	6.8.E-05	5.3.E-07	N/A
SREK1	splicing regulatory glutamine/lysine-rich protein 1	3.05	-2.08	N/A	7.5.E-04	1.4.E-03	N/A
SRSF7	serine/arginine-rich splicing factor 7	2.24	-2.68	N/A	1.6.E-02	7.1.E-04	N/A
SSPO	SCO-spondin homolog (Bos taurus)	N/A	N/A	2.09	N/A	N/A	8.0.E-05
ST14	suppression of tumorigenicity 14 (colon carcinoma)	-3.43	4.40	N/A	7.1.E-04	9.8.E-06	N/A
ST3GAL1	ST3 beta-galactoside alpha-2,3-sialyltransferase 1	2.41	-2.05	N/A	1.1.E-03	5.2.E-05	N/A
ST6GALNAC2	ST6 (alpha-N-acetyl-neuraminyl-2,3-beta-galactosyl-1,3)-N-acetylgalactosaminide alpha-2,6-sialyltransferase 2	-3.68	4.01	N/A	7.6.E-05	1.9.E-07	N/A
ST6GALNAC4	ST6 (alpha-N-acetyl-neuraminyl-2,3-beta-galactosyl-1,3)-N-acetylgalactosaminide alpha-2,6-sialyltransferase 4	N/A	-2.36	N/A	N/A	2.9.E-06	N/A
STAB1	stabilin 1	2.00	-3.38	N/A	1.0.E-03	1.2.E-05	N/A
STAM	signal transducing adaptor molecule (SH3 domain and ITAM motif) 1	-2.41	2.50	N/A	5.0.E-04	4.3.E-06	N/A
STARD7	StAR-related lipid transfer (START) domain containing 7	2.01	-2.18	N/A	3.8.E-03	2.2.E-04	N/A
STK16	serine/threonine kinase 16	N/A	N/A	-2.20	N/A	N/A	2.7.E-05
STK19	serine/threonine kinase 19	2.71	-4.35	N/A	1.1.E-03	2.2.E-05	N/A
STK24	serine/threonine kinase 24	-2.13	N/A	N/A	1.6.E-04	N/A	N/A
STMN4	stathmin-like 4	N/A	-13.68	-11.19	N/A	8.8.E-08	2.4.E-06
STRBP	spermatid perinuclear RNA binding protein	2.00	-2.32	N/A	6.3.E-04	3.9.E-06	N/A

STRN3	striatin, calmodulin binding protein 3	2.63	-3.03	N/A	2.2.E-03	1.5.E-04	N/A
STUB1	STIP1 homology and U-box containing protein 1, E3 ubiquitin protein ligase	N/A	2.46	N/A	N/A	7.1.E-06	N/A
STX11	syntaxin 11	-2.51	2.74	N/A	7.4.E-04	9.5.E-06	N/A
STXBP3	syntaxin binding protein 3	N/A	2.15	N/A	N/A	3.5.E-05	N/A
STXBP6	syntaxin binding protein 6 (amisyn)	N/A	2.18	2.21	N/A	2.6.E-04	1.5.E-04
SUPT4H1	suppressor of Ty 4 homolog 1 (S. cerevisiae)	-3.42	3.23	N/A	4.1.E-04	1.2.E-05	N/A
SUPT6H	suppressor of Ty 6 homolog (S. cerevisiae)	-3.00	4.43	N/A	2.2.E-03	6.4.E-07	N/A
SYAP1	synapse associated protein 1	N/A	-2.14	N/A	N/A	6.6.E-05	N/A
SYF2	SYF2 homolog, RNA splicing factor (S. cerevisiae)	-3.35	2.98	N/A	2.5.E-03	1.4.E-03	N/A
SYNE2	spectrin repeat containing, nuclear envelope 2	2.87	-2.97	N/A	5.6.E-04	6.4.E-05	N/A
SYNJ1	synaptojanin 1	2.38	-3.67	N/A	1.2.E-03	5.4.E-07	N/A
SYP	synaptophysin	2.04	N/A	2.05	2.5.E-03	N/A	3.3.E-04
SYPL1	synaptophysin-like 1	N/A	2.18	N/A	N/A	2.6.E-05	N/A
TACSTD2	tumor-associated calcium signal transducer 2	-2.20	2.77	N/A	1.1.E-03	8.3.E-06	N/A
TAF11	TAF11 RNA polymerase II, TATA box binding protein (TBP)-associated factor, 28kDa	-2.07	2.38	N/A	1.2.E-03	6.9.E-05	N/A
TAF13	TAF13 RNA polymerase II, TATA box binding protein (TBP)-associated factor, 18kDa	-3.95	4.44	N/A	1.2.E-04	1.6.E-08	N/A
TAF1D	TATA box binding protein (TBP)-associated factor, RNA polymerase I, D, 41kDa	N/A	-2.15	N/A	N/A	5.5.E-06	N/A
TAGLN3	transgelin 3	N/A	-2.07	N/A	N/A	3.8.E-05	N/A
TALDO1	transaldolase 1	-2.65	2.36	N/A	1.1.E-03	2.0.E-04	N/A
TANGO6	transport and golgi organization 6 homolog (Drosophila)	N/A	-2.75	N/A	N/A	1.4.E-06	N/A
TAOK3	TAO kinase 3	3.61	-4.52	N/A	3.2.E-04	1.0.E-05	N/A
TAPBP	TAP binding protein (tapasin)	-2.64	2.07	N/A	1.1.E-03	6.7.E-04	N/A
TBC1D14	TBC1 domain family, member 14	-4.97	3.82	N/A	2.0.E-03	8.8.E-04	N/A

TBC1D16	TBC1 domain family, member 16	2.44	N/A	N/A	6.4.E-04	N/A	N/A
TBC1D2	TBC1 domain family, member 2	-2.30	2.22	N/A	1.8.E-03	5.5.E-04	N/A
TBC1D7	TBC1 domain family, member 7	-2.51	2.30	N/A	5.8.E-04	2.3.E-05	N/A
TCEA1	transcription elongation factor A (SII), 1	N/A	-2.40	N/A	N/A	1.3.E-04	N/A
TCEAL4	transcription elongation factor A (SII)-like 4	N/A	N/A	-2.09	N/A	N/A	2.1.E-04
TCEB3	transcription elongation factor B (SIII), polypeptide 3 (110kDa, elongin A)	2.19	-2.75	N/A	5.0.E-04	3.4.E-06	N/A
TCHP	trichoplein, keratin filament binding	2.00	-2.32	N/A	1.6.E-03	7.9.E-04	N/A
TFDP2	transcription factor Dp-2 (E2F dimerization partner 2)	2.06	N/A	N/A	8.3.E-03	N/A	N/A
TFPI	tissue factor pathway inhibitor (lipoprotein-associated coagulation inhibitor)	N/A	-2.22	N/A	N/A	2.7.E-03	N/A
TGM1	transglutaminase 1 (K polypeptide epidermal type I, protein-glutamine-gamma-glutamyltransferase)	-2.78	3.08	N/A	3.4.E-03	2.0.E-05	N/A
THAP11	THAP domain containing 11	-4.44	3.60	N/A	5.2.E-04	6.6.E-05	N/A
THBS3	thrombospondin 3	2.04	-2.20	N/A	8.5.E-04	2.4.E-04	N/A
THOC2	THO complex 2	3.47	-3.89	N/A	6.3.E-04	2.7.E-05	N/A
THRAP3	thyroid hormone receptor associated protein 3	6.32	-6.81	N/A	7.1.E-04	9.8.E-05	N/A
TIA1	TIA1 cytotoxic granule-associated RNA binding protein	2.08	N/A	N/A	5.1.E-04	N/A	N/A
TIFAB	TRAF-interacting protein with forkhead-associated domain, family member B	2.32	-2.94	N/A	1.1.E-03	4.4.E-07	N/A
TIMM8A	translocase of inner mitochondrial membrane 8 homolog A (yeast)	-2.48	2.30	N/A	1.5.E-02	2.0.E-02	N/A
TIMP1	TIMP metallopeptidase inhibitor 1	N/A	-2.15	N/A	N/A	4.8.E-03	N/A
TJP1	tight junction protein 1	-3.62	2.89	N/A	5.5.E-04	2.6.E-04	N/A
TJP2	tight junction protein 2 (zona occludens 2)	-2.07	N/A	N/A	2.5.E-03	N/A	N/A
TLR2	toll-like receptor 2	2.63	-6.96	-2.65	9.0.E-04	1.1.E-06	6.3.E-05
TM2D1	TM2 domain containing 1	N/A	2.13	N/A	N/A	4.8.E-06	N/A

TM2D3	TM2 domain containing 3	-3.51	3.84	N/A	2.3.E-04	7.5.E-06	N/A
TM4SF1	transmembrane 4 L six family member 1	-4.07	3.59	N/A	2.6.E-04	2.1.E-06	N/A
TM7SF2	transmembrane 7 superfamily member 2	N/A	2.14	N/A	N/A	2.0.E-04	N/A
TMC8	transmembrane channel-like 8	N/A	-2.20	N/A	N/A	2.6.E-06	N/A
TMCC2	transmembrane and coiled-coil domain family 2	2.69	-3.32	N/A	3.4.E-03	1.1.E-04	N/A
TMED4	transmembrane emp24 protein transport domain containing 4	-2.70	3.13	N/A	6.0.E-04	4.1.E-05	N/A
TMEM101	transmembrane protein 101	-5.58	5.92	N/A	5.0.E-04	2.0.E-05	N/A
TMEM106B	transmembrane protein 106B	N/A	2.14	N/A	N/A	1.0.E-05	N/A
TMEM107	transmembrane protein 107	N/A	-3.08	-2.05	N/A	1.8.E-08	4.6.E-05
TMEM129	transmembrane protein 129	N/A	2.25	N/A	N/A	2.3.E-05	N/A
TMEM134	transmembrane protein 134	-2.56	2.93	N/A	1.1.E-03	4.8.E-05	N/A
TMEM14C	transmembrane protein 14C	N/A	2.07	N/A	N/A	1.1.E-03	N/A
TMEM158	transmembrane protein 158	2.02	-2.35	N/A	3.3.E-03	3.0.E-03	N/A
TMEM159	transmembrane protein 159	-2.18	2.78	N/A	2.5.E-03	2.9.E-05	N/A
TMEM170A	transmembrane protein 170A	N/A	2.39	N/A	N/A	1.2.E-04	N/A
TMEM171	transmembrane protein 171	N/A	2.34	N/A	N/A	1.9.E-04	N/A
TMEM184C	transmembrane protein 184C	-3.03	2.63	N/A	1.4.E-03	9.6.E-04	N/A
TMEM199	transmembrane protein 199	-2.03	2.44	N/A	1.6.E-03	1.4.E-04	N/A
TMEM22	transmembrane protein 22	-3.02	4.87	N/A	3.3.E-03	7.0.E-05	N/A
TMEM40	transmembrane protein 40	-3.73	4.15	N/A	2.6.E-04	1.3.E-06	N/A
TMEM42	transmembrane protein 42	N/A	2.96	N/A	N/A	8.6.E-06	N/A
TMEM43	transmembrane protein 43	-4.69	2.99	N/A	2.1.E-04	2.3.E-05	N/A
TMEM45B	transmembrane protein 45B	N/A	2.65	N/A	N/A	1.7.E-05	N/A
TMEM54	transmembrane protein 54	-2.66	2.66	N/A	6.8.E-04	9.7.E-05	N/A
TMEM59	transmembrane protein 59	-2.05	2.15	N/A	1.7.E-04	2.0.E-07	N/A

TMEM60	transmembrane protein 60	-2.35	2.86	N/A	5.3.E-04	4.6.E-06	N/A
TMEM62	transmembrane protein 62	-3.19	3.84	N/A	5.5.E-03	5.3.E-04	N/A
TMEM63B	transmembrane protein 63B	2.21	-2.64	N/A	5.6.E-04	1.3.E-05	N/A
TMEM69	transmembrane protein 69	N/A	2.07	N/A	N/A	2.5.E-05	N/A
TMEM88	transmembrane protein 88	N/A	-2.14	N/A	N/A	2.6.E-04	N/A
TMPRSS11BNL	TMPRSS11B N terminal-like	-2.93	3.70	N/A	1.4.E-03	1.0.E-05	N/A
TMTC3	transmembrane and tetratricopeptide repeat containing 3	2.45	-2.02	N/A	1.1.E-03	4.4.E-06	N/A
TMUB2	transmembrane and ubiquitin-like domain containing 2	2.28	-2.69	N/A	1.8.E-04	2.8.E-06	N/A
TMX1	thioredoxin-related transmembrane protein 1	-2.28	2.30	N/A	7.9.E-04	4.9.E-05	N/A
TMX2	thioredoxin-related transmembrane protein 2	-2.83	2.37	N/A	1.3.E-04	7.9.E-06	N/A
TNFRSF10D	tumor necrosis factor receptor superfamily, member 10d, decoy with truncated death domain	N/A	-3.14	N/A	N/A	1.6.E-05	N/A
TNPO3	transportin 3	2.15	N/A	N/A	5.0.E-04	N/A	N/A
TNXB	tenascin XB	N/A	-2.01	N/A	N/A	4.2.E-03	N/A
TOB1	transducer of ERBB2, 1	N/A	2.46	N/A	N/A	2.7.E-06	N/A
TOM1L1	target of myb1 (chicken)-like 1	N/A	2.35	N/A	N/A	2.2.E-04	N/A
TOMM20	translocase of outer mitochondrial membrane 20 homolog (yeast)	N/A	2.37	N/A	N/A	2.8.E-04	N/A
TOPORS	topoisomerase I binding, arginine/serine-rich, E3 ubiquitin protein ligase	N/A	-2.16	N/A	N/A	1.1.E-04	N/A
TOR1A	torsin family 1, member A (torsin A)	-2.12	N/A	N/A	2.6.E-04	N/A	N/A
TOX2	TOX high mobility group box family member 2	2.09	-4.28	-2.05	1.3.E-03	8.8.E-08	3.6.E-05
TPRG1	tumor protein p63 regulated 1	-3.72	4.45	N/A	2.5.E-03	2.9.E-05	N/A
TPRG1L	tumor protein p63 regulated 1-like	-2.23	2.49	N/A	9.0.E-05	1.8.E-07	N/A
TRAF4	TNF receptor-associated factor 4	N/A	-2.01	N/A	N/A	2.0.E-05	N/A
TRAPPC4	trafficking protein particle complex 4	N/A	2.56	N/A	N/A	1.6.E-07	N/A

TRDMT1	tRNA aspartic acid methyltransferase 1	2.10	-2.06	N/A	1.3.E-03	2.3.E-04	N/A
TREM1	triggering receptor expressed on myeloid cells 1	N/A	-2.93	N/A	N/A	1.9.E-05	N/A
TREML1	triggering receptor expressed on myeloid cells-like 1	N/A	-4.13	-3.88	N/A	8.9.E-08	2.3.E-05
TRERF1	transcriptional regulating factor 1	3.98	-4.67	N/A	4.0.E-04	1.2.E-05	N/A
TRIM29	tripartite motif containing 29	-2.20	2.25	N/A	7.7.E-04	8.8.E-05	N/A
TRIM41	tripartite motif containing 41	-2.61	3.31	N/A	4.1.E-03	3.9.E-04	N/A
TRIM50	tripartite motif containing 50	2.33	-2.85	N/A	2.5.E-03	4.9.E-05	N/A
TRIM64	tripartite motif containing 64	2.81	-3.23	N/A	1.6.E-03	1.5.E-05	N/A
TRIP12	thyroid hormone receptor interactor 12	2.19	-2.64	N/A	3.1.E-04	1.3.E-06	N/A
TRIP6	thyroid hormone receptor interactor 6	N/A	-2.54	N/A	N/A	8.2.E-09	N/A
TRMT11	tRNA methyltransferase 11 homolog (S. cerevisiae)	N/A	-2.86	N/A	N/A	1.9.E-06	N/A
TRMU	tRNA 5-methylaminomethyl-2-thiouridylate methyltransferase	-2.72	2.42	N/A	1.5.E-03	6.6.E-04	N/A
TRPM7	transient receptor potential cation channel, subfamily M, member 7	N/A	-2.32	N/A	N/A	6.3.E-06	N/A
TRPV2	transient receptor potential cation channel, subfamily V, member 2	N/A	-2.60	-2.05	N/A	2.2.E-06	1.8.E-03
TSEN34	tRNA splicing endonuclease 34 homolog (S. cerevisiae)	-2.13	N/A	N/A	3.8.E-03	N/A	N/A
TSKS	testis-specific serine kinase substrate	-3.00	N/A	N/A	2.4.E-03	N/A	N/A
TSPAN3	tetraspanin 3	-3.30	3.98	N/A	1.2.E-03	4.6.E-05	N/A
TSPAN31	tetraspanin 31	N/A	2.61	N/A	N/A	7.7.E-06	N/A
TSPAN32	tetraspanin 32	N/A	-2.17	N/A	N/A	3.5.E-04	N/A
TSPAN5	tetraspanin 5	N/A	-2.22	N/A	N/A	6.8.E-05	N/A
TSPAN8	tetraspanin 8	3.31	-2.12	N/A	6.7.E-03	4.6.E-03	N/A
TSPO	translocator protein (18kDa)	-3.05	3.20	N/A	4.8.E-04	2.6.E-05	N/A
TSPYL1	TSPY-like 1	-2.51	2.59	N/A	2.8.E-03	2.4.E-04	N/A

TSPYL2	TSPY-like 2	2.42	-2.39	N/A	2.1.E-03	2.7.E-04	N/A
TSPYL4	TSPY-like 4	N/A	-2.86	N/A	N/A	9.6.E-06	N/A
TSSC4	tumor suppressing subtransferable candidate 4	N/A	-2.02	N/A	N/A	3.4.E-04	N/A
TTC1	tetratricopeptide repeat domain 1	-2.25	2.72	N/A	6.9.E-04	4.5.E-05	N/A
TTC14	tetratricopeptide repeat domain 14	2.52	-3.95	N/A	4.8.E-04	6.2.E-07	N/A
TTC37	tetratricopeptide repeat domain 37	2.50	-2.78	N/A	5.6.E-04	2.7.E-04	N/A
TTC38	tetratricopeptide repeat domain 38	-2.08	2.33	N/A	2.2.E-04	2.1.E-05	N/A
TTF1	transcription termination factor, RNA polymerase I	2.38	-2.01	N/A	1.2.E-03	9.7.E-04	N/A
TUBA1A	tubulin, alpha 1a	-4.77	3.79	N/A	1.2.E-04	1.6.E-05	N/A
TUBA1B	tubulin, alpha 1b	-4.34	4.73	N/A	5.8.E-04	3.9.E-05	N/A
TUBA3E	tubulin, alpha 3e	-2.50	N/A	N/A	1.2.E-03	N/A	N/A
TUBB4B	tubulin, beta 2C	-3.65	3.31	N/A	5.6.E-04	1.1.E-04	N/A
TUFM	Tu translation elongation factor, mitochondrial	-2.44	2.08	N/A	4.5.E-04	2.7.E-04	N/A
TWF1	twinfilin, actin-binding protein, homolog 1 (Drosophila)	N/A	-2.34	N/A	N/A	5.9.E-04	N/A
TWIST2	twist homolog 2 (Drosophila)	N/A	-2.17	N/A	N/A	1.4.E-03	N/A
TXNDC5	thioredoxin domain containing 5 (endoplasmic reticulum)	-2.43	2.66	N/A	2.2.E-04	4.5.E-06	N/A
TXNIP	thioredoxin interacting protein	2.18	N/A	N/A	1.1.E-02	N/A	N/A
TXNL1	thioredoxin-like 1	-2.56	N/A	N/A	7.6.E-04	N/A	N/A
TYK2	tyrosine kinase 2	N/A	-3.73	-3.91	N/A	7.7.E-06	3.1.E-05
TYRO3	TYRO3 protein tyrosine kinase	-3.00	3.18	N/A	4.0.E-03	1.6.E-03	N/A
U2AF1	U2 small nuclear RNA auxiliary factor 1	N/A	-2.05	N/A	N/A	9.5.E-07	N/A
UBA1	ubiquitin-like modifier activating enzyme 1	-2.94	3.44	N/A	4.3.E-04	2.8.E-06	N/A
UBA5	ubiquitin-like modifier activating enzyme 5	N/A	2.73	N/A	N/A	3.1.E-04	N/A
UBAC1	UBA domain containing 1	-2.88	N/A	N/A	1.7.E-04	N/A	N/A
UBE2B	ubiquitin-conjugating enzyme E2B	-2.13	2.23	N/A	7.3.E-04	5.0.E-06	N/A

UBE2D1	ubiquitin-conjugating enzyme E2D 1	-2.54	2.84	N/A	1.2.E-02	1.7.E-03	N/A
UBE2D2	ubiquitin-conjugating enzyme E2D 2	-2.15	2.94	N/A	7.0.E-04	1.8.E-05	N/A
UBE2D3	ubiquitin-conjugating enzyme E2D 3	-2.05	2.11	N/A	1.0.E-04	1.8.E-07	N/A
UBE2D4	ubiquitin-conjugating enzyme E2D 4 (putative)	-2.44	3.18	N/A	1.4.E-03	7.0.E-06	N/A
UBE2E1	ubiquitin-conjugating enzyme E2E 1	-4.09	4.92	N/A	6.8.E-03	6.1.E-04	N/A
UBE2E2	ubiquitin-conjugating enzyme E2E 2	N/A	2.68	N/A	N/A	5.1.E-06	N/A
UBE2E3	ubiquitin-conjugating enzyme E2E 3	2.64	-5.32	-2.01	1.4.E-03	1.7.E-06	2.0.E-05
UBE2G1	ubiquitin-conjugating enzyme E2G 1	-2.84	2.88	N/A	6.8.E-04	7.0.E-05	N/A
UBE2J1	ubiquitin-conjugating enzyme E2, J1, U	-2.60	2.27	N/A	9.4.E-04	6.1.E-04	N/A
UBE2J2	ubiquitin-conjugating enzyme E2, J2	-2.33	N/A	N/A	3.8.E-05	N/A	N/A
UBE2L6	ubiquitin-conjugating enzyme E2L 6	-2.16	N/A	N/A	5.4.E-03	N/A	N/A
UBE2M	ubiquitin-conjugating enzyme E2M	-2.29	2.46	N/A	5.9.E-04	5.7.E-05	N/A
UBE2W	ubiquitin-conjugating enzyme E2W (putative)	-2.11	2.43	N/A	7.2.E-04	8.9.E-05	N/A
UBE3A	ubiquitin protein ligase E3A	N/A	2.36	N/A	N/A	8.4.E-06	N/A
UBL3	ubiquitin-like 3	-2.14	2.46	N/A	4.9.E-03	3.7.E-04	N/A
UBL7	ubiquitin-like 7 (bone marrow stromal cell-derived)	-4.69	5.09	N/A	1.7.E-04	1.5.E-06	N/A
UBR1	ubiquitin protein ligase E3 component n-recognin 1	2.31	-2.20	N/A	3.9.E-04	2.0.E-05	N/A
UBTF	upstream binding transcription factor, RNA polymerase I	3.36	-5.31	N/A	1.5.E-03	1.7.E-04	N/A
UBXN8	UBX domain protein 8	N/A	2.01	N/A	N/A	7.3.E-05	N/A
UCHL3	ubiquitin carboxyl-terminal esterase L3 (ubiquitin thiolesterase)	-2.90	2.50	N/A	2.6.E-04	7.3.E-06	N/A
UCK2	uridine-cytidine kinase 2	2.74	N/A	N/A	2.4.E-03	N/A	N/A
UEVLD	UEV and lactate/malate dehyrogenase domains	N/A	2.05	N/A	N/A	3.8.E-03	N/A
UGDH	UDP-glucose 6-dehydrogenase	-2.06	N/A	N/A	1.9.E-03	N/A	N/A
UGT1A6	UDP glucuronosyltransferase 1 family, polypeptide A6	-2.15	N/A	N/A	1.1.E-03	N/A	N/A
UHRF1	ubiquitin-like with PHD and ring finger domains 1	2.02	-2.88	N/A	2.0.E-03	1.5.E-03	N/A

UHRF1BP1L	UHRF1 binding protein 1-like	N/A	-2.94	N/A	N/A	8.8.E-04	N/A
UNG	uracil-DNA glycosylase	N/A	2.03	N/A	N/A	3.5.E-04	N/A
UPF2	UPF2 regulator of nonsense transcripts homolog (yeast)	2.70	-2.79	N/A	5.6.E-04	6.4.E-05	N/A
UPK1B	uroplakin 1B	2.31	-2.82	N/A	1.2.E-03	2.6.E-05	N/A
UPP1	uridine phosphorylase 1	N/A	2.15	N/A	N/A	3.0.E-04	N/A
UQCRC1	ubiquinol-cytochrome c reductase core protein I	-4.71	3.57	N/A	1.8.E-04	1.4.E-05	N/A
UQCRQ	ubiquinol-cytochrome c reductase, complex III subunit VII, 9.5kDa	N/A	-2.25	N/A	N/A	1.9.E-05	N/A
UROS	uroporphyrinogen III synthase	N/A	-2.18	N/A	N/A	3.4.E-05	N/A
USF1	upstream transcription factor 1	2.42	-4.19	N/A	9.7.E-04	5.9.E-06	N/A
USP15	ubiquitin specific peptidase 15	-2.51	2.56	N/A	7.5.E-04	1.6.E-04	N/A
USP34	ubiquitin specific peptidase 34	-2.20	2.36	N/A	2.2.E-03	2.2.E-04	N/A
USP42	ubiquitin specific peptidase 42	2.58	-2.89	N/A	2.4.E-03	2.5.E-04	N/A
USP50	ubiquitin specific peptidase 50	4.02	N/A	2.89	8.5.E-04	N/A	1.4.E-03
USP8	ubiquitin specific peptidase 8	-2.48	3.01	N/A	2.8.E-04	1.0.E-06	N/A
VARS	valyl-tRNA synthetase	-2.00	N/A	N/A	5.5.E-03	N/A	N/A
VARS2	valyl-tRNA synthetase 2, mitochondrial (putative)	N/A	-3.15	-3.03	N/A	8.4.E-08	6.3.E-05
VCP	valosin containing protein	-2.81	N/A	N/A	1.4.E-04	N/A	N/A
VEGFB	vascular endothelial growth factor B	N/A	-2.33	N/A	N/A	4.7.E-04	N/A
VIMP	VCP-interacting membrane protein	-2.08	N/A	N/A	4.9.E-03	N/A	N/A
VKORC1	vitamin K epoxide reductase complex, subunit 1	-2.73	3.05	N/A	3.6.E-04	2.2.E-05	N/A
VPS16	vacuolar protein sorting 16 homolog (S. cerevisiae)	2.13	-2.69	N/A	6.7.E-04	7.5.E-06	N/A
VPS18	vacuolar protein sorting 18 homolog (S. cerevisiae)	2.22	-4.36	N/A	2.2.E-04	2.0.E-08	N/A
VPS25	vacuolar protein sorting 25 homolog (S. cerevisiae)	-2.61	2.31	N/A	6.7.E-05	2.3.E-06	N/A
VPS26B	vacuolar protein sorting 26 homolog B (S. pombe)	N/A	2.20	N/A	N/A	6.4.E-04	N/A
VRTN	vertebrae development homolog (pig)	3.06	-4.08	N/A	1.2.E-03	2.9.E-07	N/A

VSIG10L	V-set and immunoglobulin domain containing 10 like	-4.82	6.40	N/A	6.7.E-04	3.9.E-06	N/A
WBP1	WW domain binding protein 1	-2.73	3.17	N/A	8.3.E-04	1.6.E-05	N/A
WDFY2	WD repeat and FYVE domain containing 2	-2.01	2.06	N/A	2.2.E-03	9.7.E-04	N/A
WDR24	WD repeat domain 24	N/A	-2.23	N/A	N/A	6.0.E-06	N/A
WDR34	WD repeat domain 34	N/A	-2.48	N/A	N/A	1.9.E-04	N/A
WDR37	WD repeat domain 37	N/A	-2.57	N/A	N/A	1.5.E-05	N/A
WDR45	WD repeat domain 45	-2.31	2.65	N/A	1.0.E-03	1.5.E-05	N/A
WDR46	WD repeat domain 46	N/A	-2.19	N/A	N/A	1.2.E-04	N/A
WDR74	WD repeat domain 74	-2.53	N/A	N/A	2.4.E-04	N/A	N/A
WDR81	WD repeat domain 81	N/A	-2.54	N/A	N/A	1.8.E-05	N/A
WDR92	WD repeat domain 92	-2.81	2.61	N/A	1.2.E-02	7.9.E-03	N/A
WHSC1L1	Wolf-Hirschhorn syndrome candidate 1-like 1	N/A	-2.11	N/A	N/A	5.1.E-03	N/A
WNT4	wingless-type MMTV integration site family, member 4	3.77	-6.75	N/A	1.6.E-03	1.3.E-05	N/A
WSB1	WD repeat and SOCS box containing 1	2.82	-4.04	N/A	8.5.E-04	8.4.E-06	N/A
XBP1	X-box binding protein 1	N/A	2.26	N/A	N/A	1.1.E-04	N/A
XPC	xeroderma pigmentosum, complementation group C	N/A	-2.02	N/A	N/A	4.6.E-05	N/A
XRCC6BP1	XRCC6 binding protein 1	-2.45	2.74	N/A	4.3.E-03	3.6.E-04	N/A
YAE1D1	Yae1 domain containing 1	2.11	-2.30	N/A	3.0.E-04	2.1.E-06	N/A
YARS	tyrosyl-tRNA synthetase	-2.28	N/A	N/A	1.4.E-04	N/A	N/A
YARS2	tyrosyl-tRNA synthetase 2, mitochondrial	2.61	-8.66	-3.32	2.6.E-03	3.3.E-06	1.3.E-05
YES1	v-yes-1 Yamaguchi sarcoma viral oncogene homolog 1	-2.65	2.52	N/A	8.7.E-04	1.4.E-04	N/A
YIF1B	Yip1 interacting factor homolog B (S. cerevisiae)	N/A	-3.55	-2.09	N/A	2.1.E-06	6.8.E-04
YIPF5	Yip1 domain family, member 5	N/A	2.00	N/A	N/A	1.6.E-03	N/A
YME1L1	YME1-like 1 (S. cerevisiae)	-2.42	3.14	N/A	6.4.E-04	2.0.E-05	N/A
YWHAZ	tyrosine 3-monooxygenase/tryptophan 5-monooxygenase activation protein, zeta polypeptide	N/A	-3.18	N/A	N/A	2.8.E-06	N/A

ZBTB20	zinc finger and BTB domain containing 20	2.40	-2.01	N/A	9.3.E-04	1.4.E-03	N/A
ZBTB7C	zinc finger and BTB domain containing 7C	-2.18	3.01	N/A	2.3.E-04	1.5.E-06	N/A
ZBTB8A	zinc finger and BTB domain containing 8A	3.69	-3.33	N/A	7.4.E-04	2.7.E-04	N/A
ZC3H10	zinc finger CCCH-type containing 10	N/A	-2.02	N/A	N/A	1.2.E-03	N/A
ZCCHC12	zinc finger, CCHC domain containing 12	N/A	-2.16	N/A	N/A	7.9.E-04	N/A
ZCCHC6	zinc finger, CCHC domain containing 6	2.04	-2.30	N/A	4.7.E-03	6.6.E-04	N/A
ZCCHC9	zinc finger, CCHC domain containing 9	2.11	N/A	N/A	2.0.E-03	N/A	N/A
ZDHHC4	zinc finger, DHHC-type containing 4	-4.15	4.41	N/A	4.3.E-04	1.7.E-05	N/A
ZFAND6	zinc finger, AN1-type domain 6	-3.26	3.76	N/A	3.0.E-04	7.8.E-06	N/A
ZFYVE26	zinc finger, FYVE domain containing 26	2.55	-2.78	N/A	1.2.E-02	1.2.E-03	N/A
ZMIZ2	zinc finger, MIZ-type containing 2	N/A	2.02	N/A	N/A	8.7.E-05	N/A
ZNF133	zinc finger protein 133	N/A	-5.13	-5.02	N/A	5.4.E-08	3.8.E-07
ZNF143	zinc finger protein 143	2.00	-3.14	N/A	5.5.E-04	1.2.E-06	N/A
ZNF217	zinc finger protein 217	N/A	-2.20	N/A	N/A	1.1.E-03	N/A
ZNF323	zinc finger protein 323	N/A	-2.62	N/A	N/A	1.6.E-05	N/A
ZNF335	zinc finger protein 335	N/A	-2.64	N/A	N/A	1.2.E-06	N/A
ZNF33B	zinc finger protein 33B	4.32	-8.21	N/A	2.2.E-03	4.4.E-05	N/A
ZNF354A	zinc finger protein 354A	N/A	-2.25	N/A	N/A	4.7.E-06	N/A
ZNF407	zinc finger protein 407	-2.22	2.05	N/A	8.8.E-04	5.4.E-04	N/A
ZNF414	zinc finger protein 414	-2.59	2.32	N/A	5.8.E-03	8.8.E-04	N/A
ZNF518A	zinc finger protein 518A	4.92	-5.67	N/A	1.1.E-04	1.9.E-06	N/A
ZNF639	zinc finger protein 639	-2.38	2.09	N/A	9.4.E-04	8.6.E-05	N/A
ZNF644	zinc finger protein 644	2.00	N/A	N/A	1.3.E-03	N/A	N/A
ZNF706	zinc finger protein 706	N/A	2.22	N/A	N/A	3.1.E-04	N/A
ZNF774	zinc finger protein 774	N/A	2.00	N/A	N/A	8.0.E-06	N/A

[1]M *vs*. E; Middle versus Early stages, L *vs*. M; Late versus Middle stages, L *vs*. E; Late versus Early stages

Supplementary Table S4. Common differentially expressed genes (fold change ≥ 2, FDR corrected $P < 0.05$) identified in comparisons of the Early and Middle, Middle and Late, and Early and Late stages.

Gene Symbol	Fold Change			Gene Name	Accession No.
	Middle *vs.* Early	Late *vs.* Middle	Late *vs.* Early		
ND6	5.84	-11.9	-2.04	NADH dehydrogenase subunit 6	
FNDC5	4.07	-10.1	-2.47	fibronectin type III domain containing 5	NM_001105421
DHTKD1	3.68	-15.8	-4.29	dehydrogenase E1 and transketolase domain containing 1	NM_001205838
NBN	3.09	-7.87	-2.55	nibrin	BC102801
RPL3	3.03	-6.66	-2.20	ribosomal protein L3	AB098822
IPO5	3.00	-8.95	-2.98	importin 5	NM_001037817
LRFN2	2.72	-15.8	-5.82	leucine rich repeat and fibronectin type III domain containing 2	NM_001192595
UBE2E3	2.64	-5.32	-2.01	ubiquitin-conjugating enzyme E2E 3	NM_001079783
TLR2	2.63	-6.96	-2.65	toll-like receptor 2	NM_174197
YARS2	2.61	-8.66	-3.32	tyrosyl-tRNA synthetase 2, mitochondrial	NM_001098088
RHOU	2.38	-44.9	-18.9	ras homolog gene family, member U	NM_001098147
PRNP	2.32	-4.69	-2.02	prion protein	NM_001271626
CHRM3	2.19	-5.32	-2.42	cholinergic receptor, muscarinic 3	NM_174270
ESF1	2.10	-4.76	-2.27	ESF1, nucleolar pre-rRNA processing protein, homolog (S. cerevisiae)	NM_001192772

TOX2	2.09	-4.28	-2.05	TOX high mobility group box family member 2	NM_001206688
HNRNPU	2.05	-4.21	-2.05	heterogeneous nuclear ribonucleoprotein U (scaffold attachment factor A)	NM_001076920
CYP46A1	-2.21	8.92	4.03	cytochrome P450, family 46, subfamily A, polypeptide 1	NM_001076810
NOV	-2.31	4.89	2.11	nephroblastoma overexpressed gene	NM_001102382
SMPD2	-2.52	5.24	2.08	sphingomyelin phosphodiesterase 2, neutral membrane (neutral sphingomyelinase)	NM_001075383
RAB19	-3.04	7.11	2.34	RAB19, member RAS oncogene family	NM_001035040
SLA2	-4.11	10.9	2.65	Src-like-adaptor 2	NM_001035294
LAMP3	-5.16	11.8	2.28	lysosomal-associated membrane protein 3	NM_001102135

Supplementary Figure S1

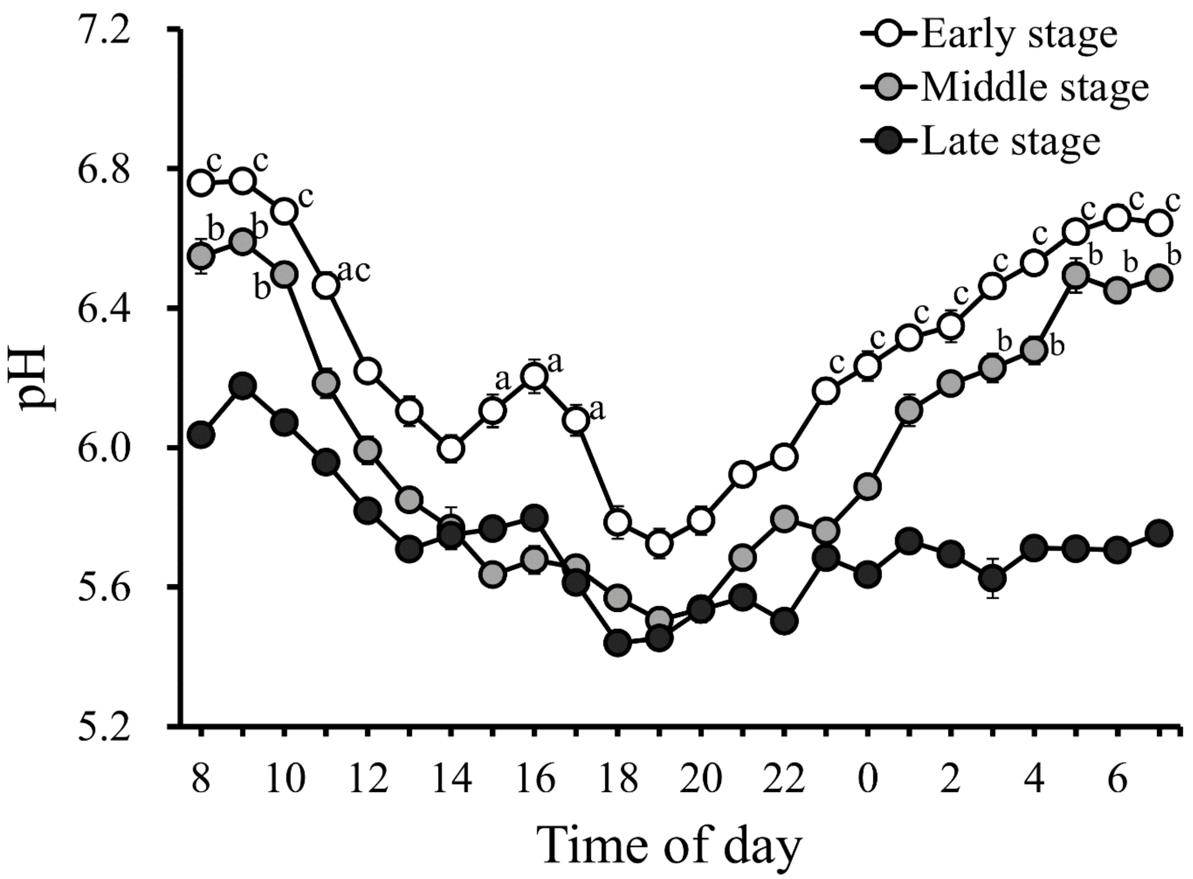

Supplementary Figure S2

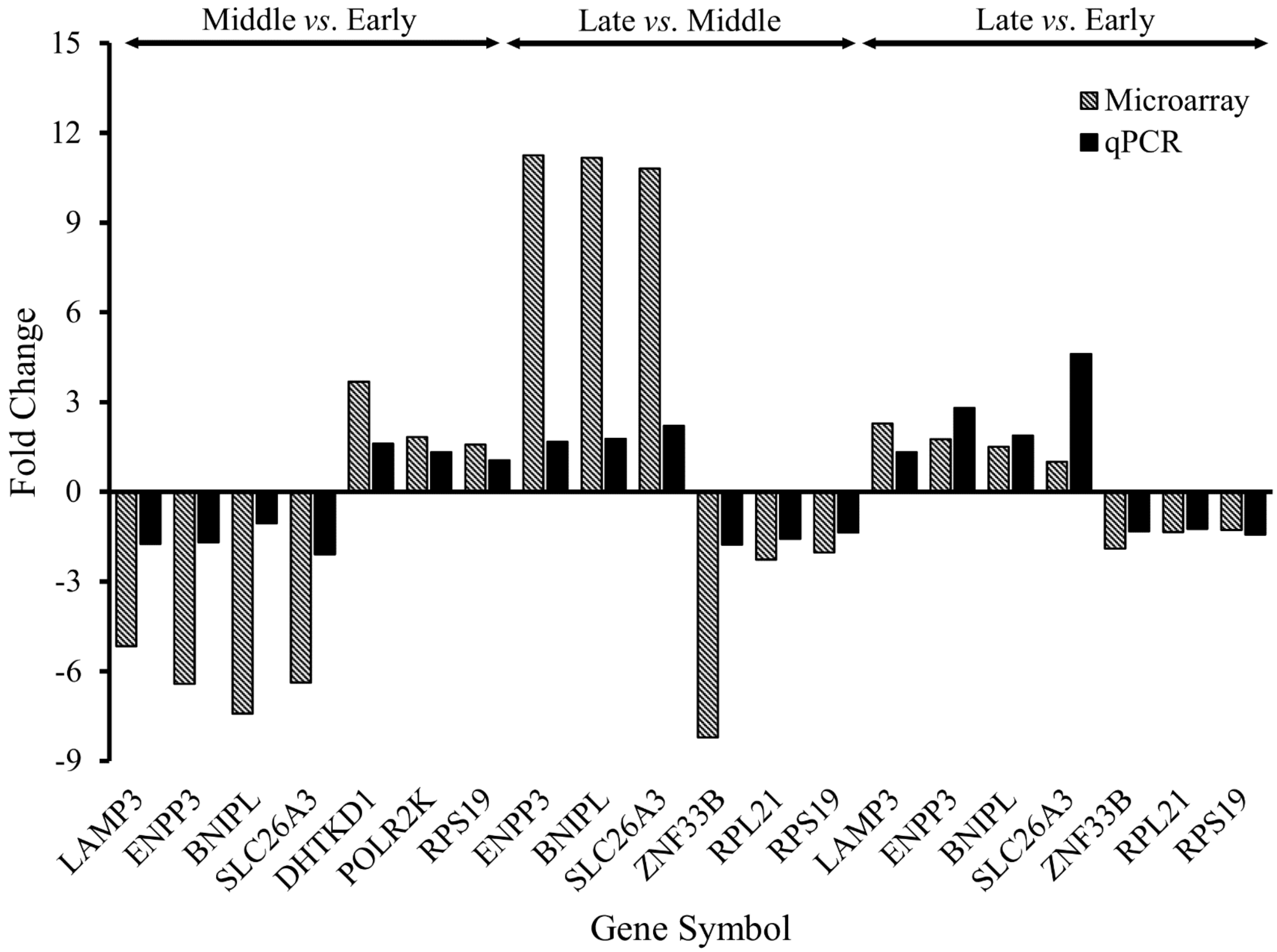

Supplement: Supplementary file 1 — Supplementary Information. [file 41598_2020_63471_MOESM1_ESM.pdf]
